# *Methylacidimicrobium thermophilum* AP8, a Novel Methane- and Hydrogen-Oxidizing Bacterium Isolated From Volcanic Soil on Pantelleria Island, Italy

**DOI:** 10.3389/fmicb.2021.637762

**Published:** 2021-02-12

**Authors:** Nunzia Picone, Pieter Blom, Anna J. Wallenius, Carmen Hogendoorn, Rob Mesman, Geert Cremers, Antonina L. Gagliano, Walter D’Alessandro, Paola Quatrini, Mike S. M. Jetten, Arjan Pol, Huub J. M. Op den Camp

**Affiliations:** ^1^Department of Microbiology, Institute for Water and Wetland Research, Radboud University, Nijmegen, Netherlands; ^2^Istituto Nazionale di Geofisica e Vulcanologia (INGV), Palermo , Italy; ^3^Department of Biological, Chemical and Pharmaceutical Sciences and Technologies (STEBICEF), University of Palermo, Palermo, Italy

**Keywords:** *Verrucomicrobia*, acidophilic, methanotroph, hydrogenase, *Methylacidimicrobium thermophilum* AP8

## Abstract

The Favara Grande is a geothermal area located on Pantelleria Island, Italy. The area is characterized high temperatures in the top layer of the soil (60°C), low pH (3–5) and hydrothermal gas emissions mainly composed of carbon dioxide (CO_2_), methane (CH_4_), and hydrogen (H_2_). These geothermal features may provide a suitable niche for the growth of chemolithotrophic thermoacidophiles, including the lanthanide-dependent methanotrophs of the phylum Verrucomicrobia. In this study, we started enrichment cultures inoculated with soil of the Favara Grande at 50 and 60°C with CH_4_ as energy source and medium containing sufficient lanthanides at pH 3 and 5. From these cultures, a verrucomicrobial methanotroph could be isolated *via* serial dilution and floating filters techniques. The genome of strain AP8 was sequenced and based on phylogenetic analysis we propose to name this new species *Methylacidimicrobium thermophilum* AP8. The transcriptome data at μ_max_ (0.051 ± 0.001 h^−1^, doubling time ~14 h) of the new strain showed a high expression of the *pmoCAB*2 operon encoding the membrane-bound methane monooxygenase and of the gene *xoxF*1, encoding the lanthanide-dependent methanol dehydrogenase. A second *pmoCAB* operon and *xoxF*2 gene were not expressed. The physiology of strain AP8 was further investigated and revealed an optimal growth in a pH range of 3–5 at 50°C, representing the first thermophilic strain of the genus *Methylacidimicrobium*. Moreover, strain AP8 had a K_S(app)_ for methane of 8 ± 1 μM. Beside methane, a type 1b [NiFe] hydrogenase enabled hydrogen oxidation at oxygen concentrations up to 1%. Taken together, our results expand the knowledge on the characteristics and adaptations of verrucomicrobial methanotrophs in hydrothermal environments and add a new thermophilic strain to the genus *Methylacidimicrobium*.

## Introduction

Methanotrophs are a diverse group of microorganisms that utilize methane (CH_4_) as energy source. By oxidizing CH_4_, they act as the carbon sink in various environments and limit the emission of this greenhouse gas to the atmosphere (Conrad, 2009). In marine anoxic sediments, this is mediated by consortia of sulfate-reducing bacteria and methanotrophic archaea (Strous and Jetten, 2004), while in oxic ecosystems, several groups of methane-oxidizing bacteria have been identified (Chistoserdova and Kalyuzhnaya, 2018). In the last decade, a new group of aerobic methanotrophs, belonging to the phylum Verrucomicrobia, has been discovered (Op den Camp et al., 2009). These verrucomicrobial methanotrophs are acidophiles so far only isolated from volcanic environments (Dunfield et al., 2007; Pol et al., 2007; Islam et al., 2008; Sharp et al., 2014; van Teeseling et al., 2014; Erikstad et al., 2019). They belong to the genera *Methylacidiphilum*, characterized by thermophilic strains and *Methylacidimicrobium*, represented by mesophilic bacteria. The verrucomicrobial methanotrophs are autotrophs, as carbon is assimilated from carbon dioxide (CO_2_) using the Calvin-Benson-Bassham (CBB) cycle (Khadem et al., 2011; van Teeseling et al., 2014). CH_4_ can be used as energy source and it is oxidized to methanol (CH_3_OH) using a copper-dependent enzyme called particulate methane monooxygenase (pMMO). The CH_3_OH in turn is oxidized to formaldehyde (CH_2_O) and formate (HCOOH), and finally to CO_2_ (Picone and Op den Camp, 2019).

Before the discovery of verrucomicrobial methanotrophs, the conversion of methanol was believed to be carried out by a calcium-dependent pyrroloquinoline quinone (PQQ) methanol dehydrogenase (Ca-MDH) encoded by the genes *mxaFI*. The genomes of verrucomicrobial methanotrophs lack *mxaFI* and accessory genes, but do possess a homologous gene called *xoxF*, that encodes an Ln-MDH with lanthanides instead of calcium in the active site (Pol et al., 2014). Lanthanides are a series of chemical elements that belong to the group of rare earth elements (REEs) and they are divided in light (La^3+^–Eu^3+^, atomic number 57–63) and heavy lanthanides (Gb^3+^–Lu^3+^, atomic number 64–71). In contrast to their name, REEs are fairly abundant in the Earth’s crust, especially in acidic geothermal soils from which all verrucomicrobial methanotrophs have been isolated to date (Pol et al., 2014). For a long time, REEs were thought to be biologically inactive due to their low solubility and thus low availability toward biological systems. However, in terms of size and ligand preference, lanthanides are similar to calcium ions. They are also stronger Lewis acids than calcium, which may provide them superior catalytic properties (Pol et al., 2014; Bogart et al., 2015; Prejanò et al., 2017; Lumpe et al., 2018). XoxF from *Methylacidiphilum fumariolicum* SolV was found to have higher affinity for methanol than the calcium-dependent MxaFI proteins (Pol et al., 2014), suggesting smaller methanol losses and higher turn-over efficiency (Krause et al., 2017). Consequently, many species with both types of MDHs have been shown to prefer the use of XoxF in the presence of lanthanides (Semrau et al., 2018). Furthermore, XoxF showed different levels of enzymatic activity depending on the type of REEs supplied, with a net preference for light lanthanides (Fitriyanto et al., 2011; Gu et al., 2016; Vu et al., 2016; Masuda et al., 2018).

Despite the progress in the characterization of different XoxF MDHs, the mechanism of lanthanides uptake is still not resolved. It was shown that in the methylotrophic bacterium *Methylorubrum extorquens* PA1, a TonB-dependent receptor and an ABC transporter are required for growth on methanol in the presence of REEs (Ochsner et al., 2019; Roszczenko-Jasińska et al., 2019). Furthermore, since the gene encoding the lanthanide binding protein Lanmodulin was located next to the TonB-dependent receptor gene, it was hypothesized that Lanmodulin would be associated with this receptor, bind REEs, and transport them to the periplasm (Cotruvo et al., 2018). However, genes encoding such a protein are not present in the genomes of verrucomicrobial methanotrophs.

Apart from metabolizing CH_4_, verrucomicrobial methanotrophs can also use hydrogen (H_2_) for growth. The thermophilic *M. fumariolicum* SolV genome encodes two [NiFe] hydrogenases, belonging to the groups 1d (O_2_ sensitive) and 1 h/5 (O_2_ insensitive; Mohammadi et al., 2017a), with the latter being able to oxidize H_2_ at subatmospheric concentrations (Schmitz et al., 2020). Furthermore, *Methylacidiphilum* sp. RTK17.1 was shown to constitutively oxidize H_2_ and this strain encoded two [NiFe]-hydrogenases belonging to groups 1d and 3b (Carere et al., 2017). The mesophilic *Methylacidimicrobium* species only encode a type 1b [NiFe] hydrogenase, which is classified as O_2_ sensitive and involved in anaerobic respiration (Greening et al., 2016). However, the strict oxygen sensitivity of this protein was recently challenged by a study which showed that *Methylacidimicrobium tartarophylax* 4AC could consume H_2_ in the presence of up to 40 μM of O_2_ (Mohammadi et al., 2019).

Hydrogenases are enzymes characterized based on their tolerance to O_2_ and can be found in different environments, including geothermal areas (Greening et al., 2016). Hydrothermal fluxes with high content of hydrogen gas are often present in volcanic ecosystems, where microorganisms can use it as energy source. H_2_ was also detected in the gas emitting from the geothermal site of Favara Grande, located on Pantelleria Island, Italy. The Favara Grande is the main exhalative site on the volcanic island, and characterized by high temperatures (60°C at the surface and 100°C at 50 cm depth), acidic pH (3–5), and gas emissions mainly consisting of CO_2_, CH_4_, and H_2_ (D’Alessandro et al., 2009; Gagliano et al., 2016). Based on its geochemical features, Pantelleria Island could be a hotspot for the growth of (verrucomicrobial) hydrogenotrophs and methanotrophs. Previous *pmoA* PCR libraries, performed with newly designed PCR primers, had detected uncharacterized verrucomicrobial methanotrophs in Pantelleria soil (Gagliano et al., 2014). However, follow-up 16S rRNA gene analysis of site FAV1 and FAV2 in the Favara Grande did not show the presence of these methanotrophs, but the methanotrophic community appeared to be mainly consisting of Proteobacteria (Gagliano et al., 2016). The aim of this study was to mine uncultivated methanotrophic microorganisms and to enrich them using soil samples from the Favara Grande site.

Here, we report the isolation and characterization of a novel Verrucomicrobia species, named *Methylacidimicrobium thermophilum* AP8. Based on its genome analysis and physiological characterization, we show that strain AP8 is a chemolithoautotrophic and thermoacidophilic microorganism with a faster growth rate than other members of this genus. Its affinity toward methane was in the same range as reported for other methanotrophs and the new isolate was able to consume H_2_ gas at low pH values under microaerobic conditions.

### Materials and Methods

### Sampling and Enrichments

Soil samples were collected in June 2017 from Favara Grande, a geothermal area on the volcanic island of Pantelleria (site FAV1, N 23°21'80'', E 40°73'17''). The top layer of a soil core was used as inoculum in air-tight serum bottles containing 10 ml of Pantelleria medium (see below) adjusted to pH 3 and 5. The headspace of the bottles was composed of 87% N_2_, 10% CO_2_, 1.5% air, and 1.5% CH_4_. Cultures were incubated at 50 and 60°C and shaking at 100 rpm.

### Isolation Procedure and Batch Cultivation

Pure cultures of *M. thermophilum* AP8 were obtained from enrichment cultures originating from the soil top layer of FAV1, using dilution-to-extinction and floating filter techniques, as described previously (De Bruyn et al., 1990). Briefly, soil samples (15 ml) were put into 50 ml sterile plastic tubes and 15 ml of Pantelleria mineral medium (see below) was added. After shaking for 2 min and settling for 3 min, 1 ml of the liquid phase was used for serial dilutions (20-fold dilution steps) in 60 ml serum bottles. Bottles were incubated at 50 and 60°C and at pH 3 and 5 in Pantelleria mineral medium with shaking at 100 rpm. Highest dilution showing CH_4_ consumption were transferred three times, serially diluted to 10^8^ and the dilutions were transferred onto floating membrane filters and incubated as described before (de Bruyn et al., 1990; Pol et al., 2007). Single colonies that appeared on the membranes were transferred to liquid medium. Cultures were grown in Pantelleria medium, containing 1 mM Na_2_SO_4_, 2 mM K_2_SO_4_, 0.5–5 mM (NH_4_)_2_SO_4_, 1 mM NaH_2_PO_4_.2H_2_O, 2 mM MgSO_2_.7H_2_O, 2 mM CaCl_2_.2H_2_O, 5 μM nitrilotriacetic acid (NTA), 0.5 μM CeCl_3_ or Nd_2_O_3_, 0.5 μM Na_2_SeO_3_, and a trace element solution containing 10 μM FeSO_4_, 10 μM MnCl_2_, 15 μM CuSO_4_, 5 μM NiCl_2_, 0.5 μM ZnSO_4_, 0.5 μM Na_2_MoO_4_, and 0.5 μM CoCl_2_. The trace elements solutions, CaCl_2_.2H_2_O, NTA, CeCl_3_/Nd_2_O_3_, and Na_2_SeO_3_, were autoclaved separately and added afterward, to avoid precipitation. The pH was adjusted with 1 M H_2_SO_4_ or 0.1 M NaOH using a 691 pH meter (Metrohm). Bottles were incubated at 50°C with 100 or 250 rpm shaking. To reach maximum growth rate, the headspace contained 49–64% N_2_, 10–20% CH_4_, 5–10% CO_2_, and 21% O_2_.

### Chemostat Cultivation

A bioreactor (Applikon Biotechnology, Delft, Netherlands) containing 10 L of Pantelleria medium was inoculated with 300 ml of *M. thermophilum* AP8 batch culture at OD_600_ 0.3 and operated at 50°C with stirring at 1000 rpm. To achieve maximum growth rate, CH_4_/CO_2_ (95:5) was sparged through a sterile filter with a flow rate of 169 ml/min. The dO_2_ was regulated at 5% O_2_ and growth of the culture was recorded by measuring the OD_600_ with Spectronic 200 spectrophotometer (Thermo Science) in 1 ml microcuvettes. After achieving maximum growth rate, the stirring was decreased to 500 rpm and CH_4_/CO_2_ was limited to a flow rate of 11.6 ml/min. The chemostat was supplied with Pantelleria medium [with 20 mM (NH_4_)_2_SO_4_] at a dilution rate of 0.005 h^−1^ (50 ml.h^−1^). The pH was kept stable with controlled supply of 0.5 M NaOH.

### pH and Temperature Optima

Cultures of *M. thermophilum* AP8 were incubated in Pantelleria medium from pH 1.5 to 5.5. For pH 4 and above, medium was buffered with 50 mM 2-(N-morpholino)ethanesulfonic acid (MES), as described previously (van Teeseling et al., 2014). Bacterial growth was determined by an increase in density and measured as the OD_600_ in standard microcuvettes in a Cary 60 photospectrometer (Agilent Technologies, Santa Clara, CA, United States), or in 96-well plate in the SpectraMax 190 plate reader (Molecular Devices, San Jose, CA, United States). During and after experiments, pH was measured using the Hanna HI 5221 pH meter.

For temperature optimum experiments, cultures were incubated in Pantelleria medium at pH 4 in shaking incubators at the following temperatures: 30, 37, 45, 50, 55, and 60°C. Bacterial growth was determined by an increase in density, measured as OD_600_ in 96-well plate in the SpectraMax 190 plate reader (Molecular Devices). All experiments were conducted in triplicate.

### Kinetics of Methane Oxidation

Exponentially growing *M. thermophilum* AP8 cells (OD_600_ 0.2–0.5) were transferred to a capped 120 ml serum bottle. Methane was injected into the bottles to reach a concentration of 0–80 μM CH_4_. To ensure active methane oxidation, the bottles were incubated at 50°C with 350 rpm shaking for 20 min prior to the start of measuring. Consumption of methane was measured over a 90 min period by injecting 100 μl of headspace gas with a glass syringe into a HP 5890 gas chromatograph (Agilent) equipped with a Porapak Q column and a flame ionization detector. The experiment was conducted in triplicate. The obtained methane consumption rates were normalized against the cell density measured as OD_600_. Origin Software (OriginLab Corporation) was used to fit a Michaelis–Menten curve and calculate the K_s_ and V_max_ values according to the equation V = V_max_ ∙ [S}/(K_s_+[S]), in which V was measured as methane consumption rate and [S] represents the methane concentration.

### Hydrogen Consumption

*Methylacidimicrobium thermophilum* AP8 cells grown under CH_4_ limitation were inoculated into sterile capped 120 ml serum bottles. Bottles were made anaerobic by using a mixture of Ar/CO_2_ (90%/10%, v/v) and supplied with 5% H_2_ and a varying concentration of O_2_ (0–5%). Starting OD_600_ was 0.1 in 20 ml of medium. The bottles were incubated at 50°C with 350 rpm shaking. The H_2_ consumption was measured by injecting 50 μl of headspace gas with a glass syringe into a HP 5890 gas chromatograph (Agilent) equipped with a Porapak Q column and a thermal conductivity detector. The respective H_2_ consumption rates were determined by converting the peak areas into percentage of H_2_ using a calibration curve. The growth on H_2_ was detected by measuring OD_600_ every 24 h with Spectronic 200 spectrophotometer (ThermoFisher) in 1 ml microcuvettes. The experiment was conducted in triplicate.

### DNA Extraction and Sequencing

gDNA of *M. thermophilum* AP8 was isolated using the DNeasy Blood&Tissue kit according to manufacturer’s instructions (Qiagen, Venlo, Netherlands). A combination of Illumina and Nanopore sequencing was used to obtain the complete genome.

Libraries for Illumina sequencing were prepared using the Nextera XT kit (Illumina), followed by enzymatic tagmentation, incorporation of indexed adapters, and amplification of the library. The amplified library was purified with AMPure XP beads, after which quality and size distribution were checked with the Agilent 2,100 Bioanalyser and High sensitivity DNA kit. After fluorimetric quantitation using Qubit dsDNA HS Assay Kit (ThermoScientific), the library was normalized and diluted to a final concentration of 4 nM. Pooled libraries were denatured and diluted using Denature and Dilute Libraries Guide (Illumina) and loaded into cartridge of the Illumina Miseq sequence machine. Paired-end sequencing of 2 × 300 bp was used.

Nanopore MinION sequencing was performed by shearing the DNA using Covaris g-TUBE (Covaris). Sheared fragments were end-repaired using NEBNext FFPE DNA Repair Mix (New England Biolabs) and purified using AMPure XP beads. Barcodes were ligated using Blunt/TA Ligase Master Mix (NEB) and cleaned using AMPure XP beads. Adapters were ligated using NEBNext Quick Ligation Module (NEB). The library was purified with AMPure XP beads and quantified using Qubit dsDNA HS Assay Kit (ThermoScientific).

### Genome Assembly and Annotation

A total of 6,769,626 sequenced Illumina reads were trimmed (quality limit = 0.05, ambiguous limit = 2 and minimum number of nucleotides in reads = 150) using CLC Genomics Workbench 11.0 (Qiagen, Aarhus, Denmark), resulting in 6,237,920 trimmed reads. After base calling and quality assessment with ONT Albacore Sequencing Pipeline Software 2.1.10 (Nanopore, Oxford, England), 141,891 Nanopore reads passed the check (N50 = 7,532 bp). The Nanopore reads were assembled with Canu (genomeSize = 2.2 m, stopOnReadQuality = false), resulting in one circular contig of 2,298,237 bp. The assembly was optimized with Racon (default settings; Vaser et al., 2017), followed by two iterations of Pilon (default settings; Walker et al., 2014) using the trimmed Illumina reads. The complete genome of 2,300,970 bp was then uploaded to the MicroScope annotation platform (Vallenet et al., 2006, 2009) and the genes annotated by using a combination of protein-BLAST, UniProtKB/TrEMBL(Consortium, 2018) and SWISS-PROT (Bairoch and Apweiler, 1996) databases. The hydrogenase classification web tool hydDB was used to classify catalytic subunits of putative hydrogenases (Søndergaard et al., 2016).

### Phylogenetic Analysis

Nucleotide (16S rRNA) and protein (PmoA, XoxF, and HynB) sequences of strain AP8 were used in Blast searches against Genbank databases. Representative homologous sequences were downloaded and aligned by the ClustalW or MUSCLE tool available in MEGA7 (Kumar et al., 2016). MEGA7 was also used to infer the evolutionary history of the representative genes and proteins using the Neighbor-Joining method.

### RNA Extraction and Sequencing

A sample (3 ml) of bacterial culture, grown at maximum growth rate and OD_600_ 3.5, was centrifuged for 10 min at 4,400 rpm, supernatant removed, and the pellets frozen at −80°C. RNA was extracted from thawed cells using RiboPure Bacteria Kit according to manufacturer instructions (ThermoFisher). The total RNA concentration was measured with Qubit Fluorometer RNA HS assay kit (ThermoFisher). The total RNA was purified with MICROBexpress Kit (ThermoFisher) to remove non-coding RNA and the quality of the rRNA-depleted RNA was checked using the Agilent 2,100 Bioanalyzer (Agilent). A cDNA library was prepared from the rRNA-depleted samples using Ion Total RNA-seq Kit v2 according to the manufacturer protocol (Thermofisher). Briefly, RNA was first fragmented, and the fragments were purified with magnetic beads. The fragments were hybridized and ligated with random primers for cDNA production by reverse transcription and the cDNA was purified with magnetic beads. Then, cDNA was amplified with PCR into a barcoded library and the quality of the PCR products checked with Agilent 2,100 Bioanalyzer using the High Sensitivity DNA Kit (Agilent). The concentration of cDNA was measured using a Qubit Fluorometer dsDNA HS Assay (ThermoFisher). Samples were diluted and loaded into a cartridge of the Illumina Miseq sequencing machine. The sequence reads were analyzed using CLC Genomic Workbench software (version 12, Qiagen). Reads shorter than 100 bp were trimmed and non-coding rRNA sequences were excluded to obtain the expression values of mRNA sequences presented as reads per kilobase per million reads (RPKM; Mortazavi et al., 2008).

### Electron Microscopy

Biomass (4 ml) was harvested from a bioreactor and concentrated by centrifugation (800 *g*, 4 min). The pellet was resuspended in 15 μl supernatant and subsequently, 0.6 μl samples were high-pressure frozen in a HPM-100 (Leica Microsystems) using gold-plated platelets (2 mm inner diameter and 100 μm sample thickness). Care was taken to keep the cells at their optimal temperature of 50°C until the very moment of freezing. Samples were freeze-substituted in an AFS2 (Leica Microsystems) using anhydrous acetone (Seccosolv, Merck Millipore, Darmstadt, Germany) containing 0.2% Uranyl Acetate (Merck, Darmstadt, Germany). The substitution started at −90°C for 48 h, followed by a +2°C/h slope to −70°C, where the sample remained for 12 h. Afterwards, the temperature was raised with 2°C/h to −50°C, where the sample remained for 12 h. Next, the sample was washed twice with anhydrous acetone at −50°C and stepwise infiltrated with Lowicryl HM20 (Electron Microscopy Sciences, Hatfield, PA, United States) in anhydrous acetone at −50°C. After the four changes of 100% HM20, the samples were polymerized by UV irradiation at −50°C for 96 h and +2°C/h to 0°C followed by 24 h at 0°C. Ultrathin sections (ca. 50 nm) were cut using an ultramicrotome (Ultracut, Reichert-Jung, Vienna, Austria) and applied to 100 mesh copper grids (Stork-Veco, Eerbeek, Netherlands) containing a carbon-coated formvar film. Micrographs were recorded in a JEOL Jem-1400 Flash transmission electron microscope operating at 120 kV.

## Results

### Isolation of a Novel Verrucomicrobial Methanotroph From Pantelleria Island

The Favara Grande is a geothermal area located in Pantelleria Island, Italy, characterized by pH values a low as 3 and temperatures up to 60°C in the top layer of the soil. Hydrothermal gases are mainly composed of CO_2_, CH_4_, and H_2_ which allow the establishment of a chemolithoautotrophic microbial community that includes methane-oxidizing microorganisms (Gagliano et al., 2016). In an attempt to enrich methanotrophic key players, volcanic soil from Pantelleria was used as inoculum in batch cultures with CH_4_ as energy source. These bottles were incubated at 50 and 60°C in defined mineral medium containing sufficient REEs at pH 3 and 5. The concentration of CH_4_ in the headspace for all enrichment cultures was measured weekly. Active cultures were then diluted into fresh medium and one of them, grown at 50°C and pH 3, retained activity after dilution. 16S rRNA gene analysis performed on gDNA extracted from this enrichment showed the presence of a bacterial species having 97.2% identity to *Methylacidimicrobium cyclopopanthes* 3C, a methanotrophic species from the phylum Verrucomicrobia (van Teeseling et al., 2014). From this bottle, a pure culture was obtained *via* serial dilution to extinction and floating filter techniques (De Bruyn et al., 1990; Pol et al., 2007).

### The Genome of the Isolated Methanotroph

**Figure 1:**
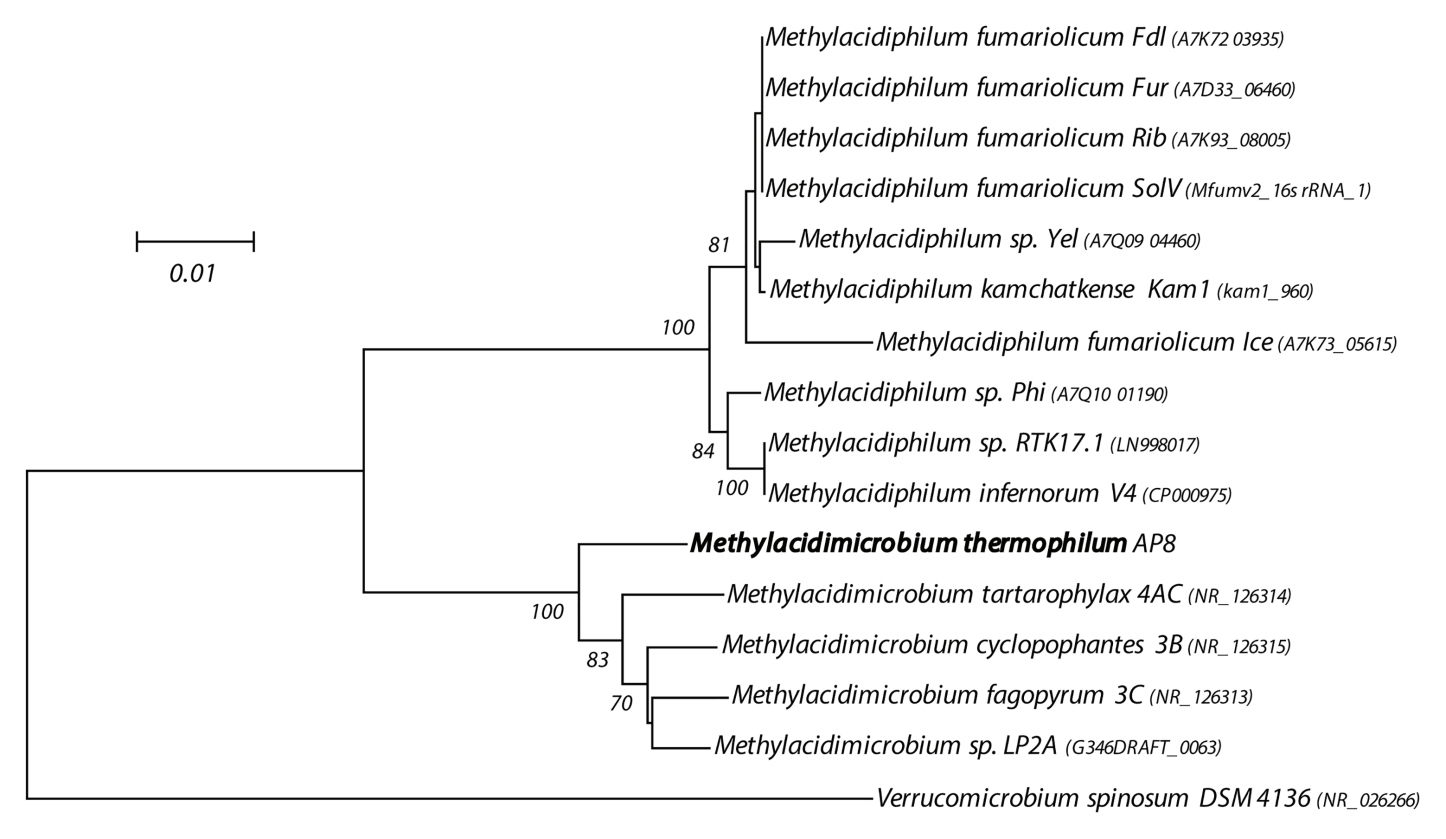
16S rRNA gene-based phylogenetic tree of methanotrophic Verrucomicrobia. The evolutionary history was inferred using the Neighbor-Joining method. The optimal tree with the sum of branch length = 0.21708868 is shown. The percentage of replicate trees (>60%) in which the associated taxa clustered together in the bootstrap test (500 replicates) are shown next to the branches. The tree is drawn to scale, with branch lengths in the same units as those of the evolutionary distances used to infer the phylogenetic tree. The evolutionary distances were computed using the Maximum Composite Likelihood method and are in the units of the number of base substitutions per site. The analysis involved 16 nucleotide sequences. All positions containing gaps and missing data were eliminated. There were a total of 1,393 positions in the final dataset. Evolutionary analyses were conducted in MEGA7 (Kumar et al., 2016).

The gDNA of the novel isolate was sequenced with a combination of Illumina and Nanopore sequencing. Assembly using all reads resulted in a single circular chromosome of 2.3 Mb with no plasmids, 2,361 CDSs, and a GC-content of 64.3% (Supplementary Figure S1). Average CDS length was 915.75 and protein coding density 91.2%. The genome contained a single rRNA operon. 16S rRNA gene sequence phylogenic analysis (Figure 1) revealed a clear separation from sequences of the other *Methylacidimicrobium* species. This is also supported by the phylogenetic analysis of the key marker enzymes PmoA, XoxF, and HynB (Supplementary Figures S2–S4) Furthermore, ANI (Supplementary Table S1) and 16S rRNA identity value fell well below the threshold for species delimitation (Mende et al., 2013; Kim et al., 2014), indicating that a novel species was isolated, for which we propose the name *M. thermophilum* AP8.

### *Methylacidimicrobium thermophilum* AP8 Shows Potential for CH_4_ and H_2_ Oxidation

**Figure 2:**
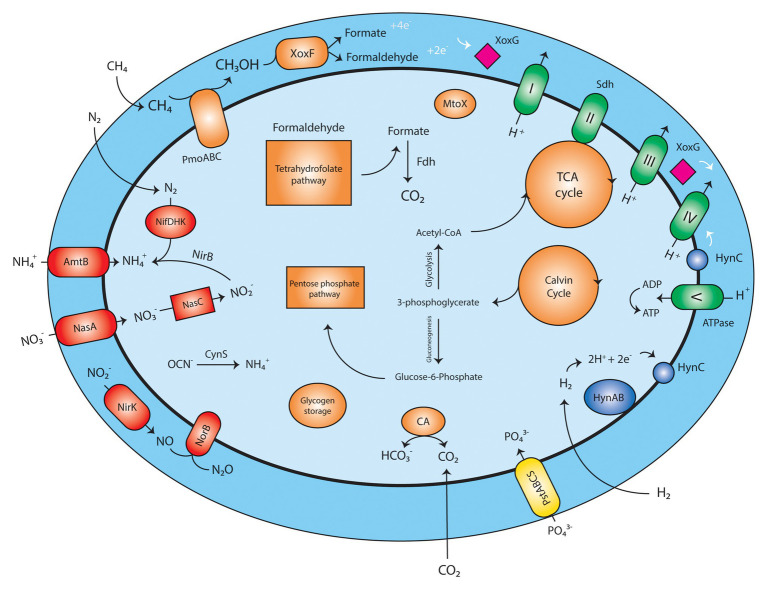
Schematic reconstruction of *Methylobacterium thermophilum* AP8 metabolism. Red, nitrogen metabolism; Orange, carbon metabolism; Green, respiratory chain; Yellow, phosphate transporter; Blue: hydrogenase; Pink, cytochrome; PmoABC, particulate methane monooxygenase; XoxF, methanol dehydrogenase; XoxG, cytochrome C_L_; Fdh, formate dehydrogenase; MtoX, methanethiol oxidase; CA, carbonic anhydrase; Sdh, succinate dehydrogenase; PstABCS, phosphate transport system; HynABC, small, large, and cytochrome b subunit of the group 1b [NiFe] hydrogenase; NifDHK, nitrogenase; AmtB, NH3/NH4+ transporter; NasA, nitrate transporter; NasC, assimilatory nitrate reductase; NirK, nitrite reductase; NorB, nitric oxide oxidoreductase; CynS, cyanate hydratase. A list of all annotated genes can be found in Supplementary Table S2.

Analysis of the *M. thermophilum* AP8 genome predicted that this bacterium would be able to use CH_4_ as energy source (Figure 2; Supplementary Table S2). Two complete operons encoding the particulate methane monooxygenase (pMMO) could be identified (*pmoCAB*1 MTHMO_v1_0893-0895 and *pmoCAB*2 MTHMO_v1_0896-0898) together with a single separate *pmoD* gene (MTHMO_v1_0906; Supplementary Figure S5). The methanol generated by the pMMO would subsequently be converted by the lanthanide dependent MDH (XoxF-type) to formaldehyde and/or formate. Also, for the XoxF protein, two copies of the encoding gene were identified, *xoxF1* (MTHMO_v1_1700) and *xoxF2* (MTHMO_v1_1756; Supplementary Figure S5). The two proteins shared 49% identity. The mechanism of REE transport into microbial cells is not completely clarified; however, *M. thermophilum* AP8 encoded several genes that might be involved in lanthanide uptake. The gene *cirA* (MTHMO_v1_1697) encodes for a homologous of the TonB dependent transporter of *Methylobacterium extorquens* PA1 (Ochsner et al., 2019). The lanthanide binding protein Lanmodulin, instead, was not found in the genome (Cotruvo et al., 2018). Formaldehyde dehydrogenase was also not identified, whereas a gene cluster for formate dehydrogenases (MTHMO_v1_0868, MTHMO_v1_1065-1068) was predicted.

The electrons generated during methane oxidation can be carried to the respiratory chain by a cytochrome C_L_ encoded by *xoxG* (MTHMO_v1_1702).

The presence of a carbonic anhydrase (MTHMO_v1_0262) and of enzymes for the Calvin cycle (Supplementary Table S2), indicated that strain AP8 carried the potential to fix CO_2_. Furthermore, a methanethiol oxidase (MTHMO_v1_1990) was detected in the genome; this enzyme converts methanethiol to H_2_O_2_, formaldehyde, and H_2_S. Toxic formaldehyde can be metabolized by XoxF or by the tetrahydrofolate-dependent formaldehyde oxidation pathway, where the gene *folD* (MTHMO_v1_0721), encoding a methylenetetrahydrofolate dehydrogenase/cyclohydrolase generates formate, that can be converted to CO_2_ by formate dehydrogenase (Vorholt, 2002). Genes for the TCA cycle, pentose phosphate pathway, glycolysis, and gluconeogenesis were also identified (Supplementary Table S2). Glycogen storage could be carried out by a glycogen synthase (MTHMO_v1_0055, 2120).

Beside CH_4_, H_2_ seemed to be a possible substrate for *M. thermophilum* AP8, as a type 1b [NiFe] hydrogenase (MTHMO_v1_1378-1380) was detected in the genome (Supplementary Figure S4). This type of hydrogenase was considered functional and exclusively supporting chemolithoautotrophic growth under anaerobic conditions (Søndergaard et al., 2016), but this paradigm was already overturned by our previous report on H_2_ oxidation by *M. tartarophylax* 4AC under microoxic conditions (Mohammadi et al., 2019).

Similar to other verrucomicrobial methanotrophs (Khadem et al., 2010), *M. thermophilum* AP8 is potentially able to fix N_2_ through a nitrogenase enzyme (MTHMO_v1_1014-1019). NH_4_^+^ and NO_3_^−^ can also be transported inside the cells by NH_4_^+^ (Amtb, MTHMO_v1_0690, 0691) and NO_3_^−^ transporters (NasA, MTHMO_v1_1025, 1031). Finally, cyanate can be converted to NH_4_^+^ by a cyanate hydratase (CynS, MTHMO_v1_1705). So N_2_ gas, NH_4_^+^, NO_3_^−^, and cyanate could serve as nitrogen source and ammonium can be incorporated into biomass *via* the glutamate dehydrogenase (GdhA, MTHMO_v1_2125) and/or glutamine synthetase/glutamate synthase (GlnA, GltS, MTHMO_v1_1185, 1987) pathways. Nitrate (NO_3_^−^) would be converted *via* assimilatory nitrate reductase (NasC, MTHMO_v1_1029) into nitrite and further on to NH_4_^+^ *via* nitrite reductase (NirB, MTHMO_v1_1028).

Under oxygen-limitation, nitrite can be transformed to nitric oxide (NO) by a copper-containing nitrite reductase (NirK, MTHMO_v1_0885). NO would then be converted to nitrous oxide (N_2_O) by nitric oxide reductase (NorCB, MTHMO_v1_1794, 1795) as also described by (Mohammadi et al., 2017b).

NH_4_^+^ can accidentally be oxidized by pMMO to hydroxylamine (NH_2_OH). NH_2_OH needs to be rapidly metabolized due to its toxicity and potential inhibition of XoxF (Versantvoort et al., 2020). However, contrary to other Verrucomicrobia species (Mohammadi et al., 2017b), no hydroxylamine dehydrogenase could be detected, and thus other mechanisms might be employed to detoxify NH_2_OH.

### Physiological Characteristics of Strain AP8

*Methylacidimicrobium* species discovered in acidic mud volcanoes are known to be mesophilic and to have a pH range for growth between 0.5 and 6 (Sharp et al., 2014; van Teeseling et al., 2014). Growth of *M. thermophilum* AP8 was observed in presence of NH_3_ and NO_3_^−^ as nitrogen source, but not with NO_2_^−^.

**Figure 3:**
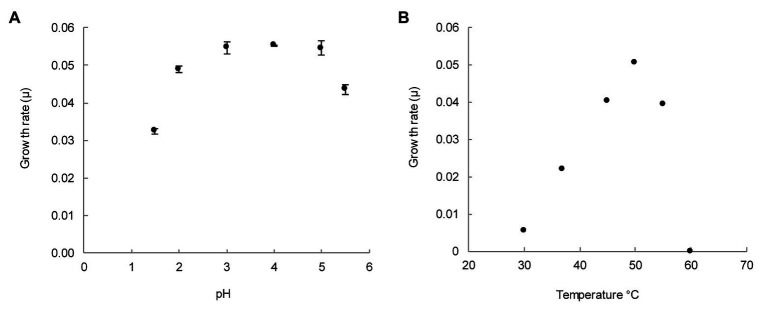
pH and temperature range for the growth of *M. thermophilum* AP8. **(A)** Growth rate per hour (μ) for six different pH values. Growth rates were determined for exponentially growing cultures for which pH remained constant. The error is expressed as the standard deviation over three biological replicates. **(B)** Growth rate per hour (μ) for temperatures from 30 to 60°C. Maximum growth rate (μ_max_) was observed at 50°C (μ = 0.05 h^−1^). The error is expressed as the SD over three biological replicates. Where not visible, error bars are smaller than the symbols.

For determination of pH optimum, batch incubations with medium ranging from pH 1.5 to 5.5 were tested. As shown in Figure 3A, the growth rate reached its maximum between pH 3 and 5. Nevertheless, growth could still be observed at pH 1.5 and 5.5.

Growth was minimal at 30°C and maximal at 50°C (Figure 3B). No growth was observed at 60°C. These experiments showed that *M. thermophilum* AP8 grew optimally at 50°C at a pH ranging from 3 to 5, and it exhibited a maximum growth rate (μ_max_) of 0.051 ± 0.001 h^−1^. This corresponded to a doubling time of approximately 14 h.

**Figure 4:**
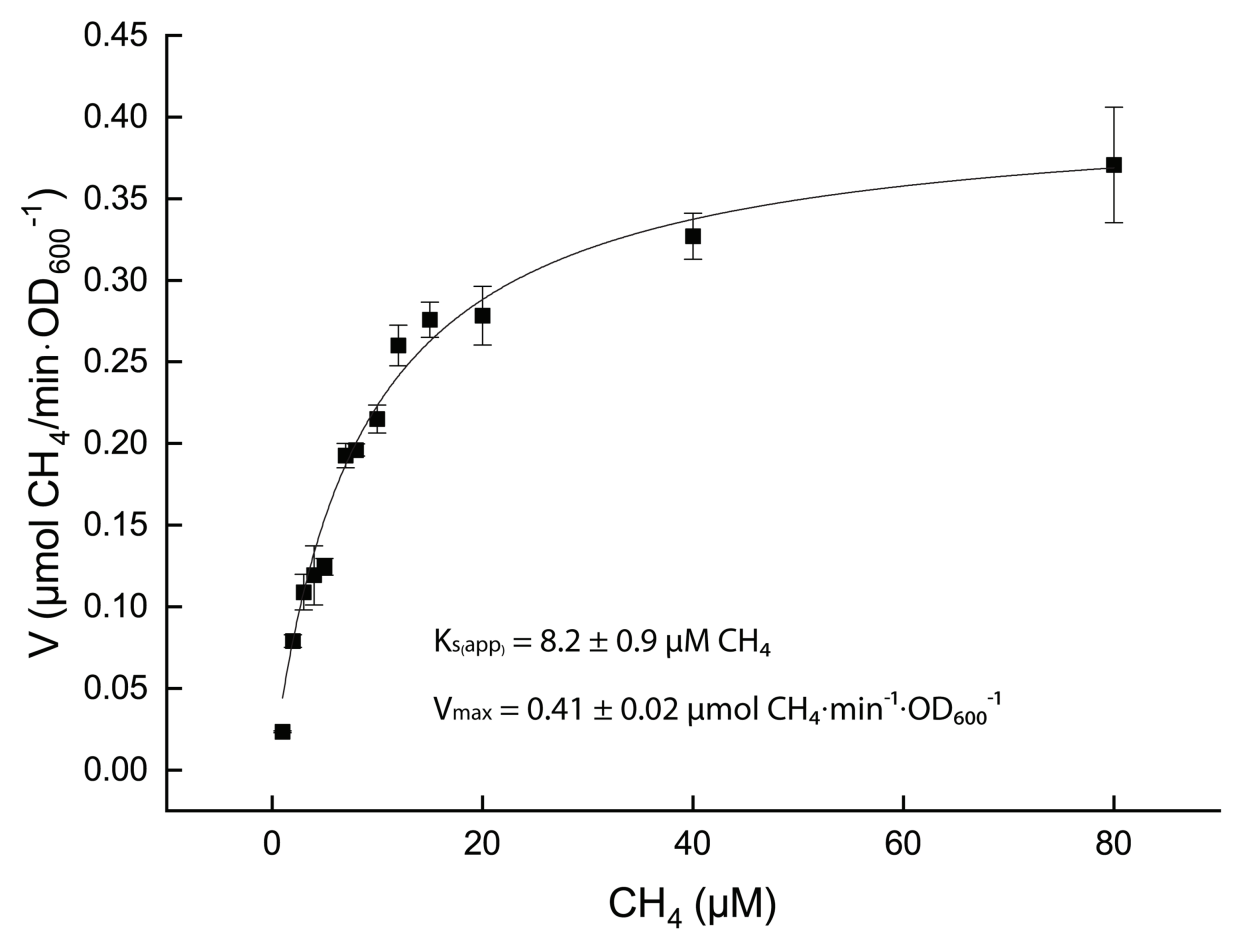
Kinetics of methane oxidation. Black squares represent the average over three biological replicates and the SD in expressed as error bars. The black curve is the result of the best fitting curve for a Michaelis-Menten kinetics (R^2^ = 0.999).

Once optimal growth conditions were established, cells at μ_max_ were used to measure the affinity constant for CH_4_ (K_s(app)_). Cultures were incubated in bottles containing 0–80 μM CH_4_ and the consumption of methane was monitored over time (methane solubility in aqueous solution at 50°C and 1 bar: 0.0012 m/Kg (Duan et al., 1992)). The CH_4_ consumption rates, standardized by the cell density expressed as OD_600_, were plotted against the CH_4_ concentrations. These values were fitted to a Michaelis-Menten model, which resulted in a K_s(app)_ of 8.2 ± 0.9 μM CH_4_ (Figure 4).

### The Cell Morphology of *Methylacidimicrobium thermophilum* AP8

**Figure 5:**
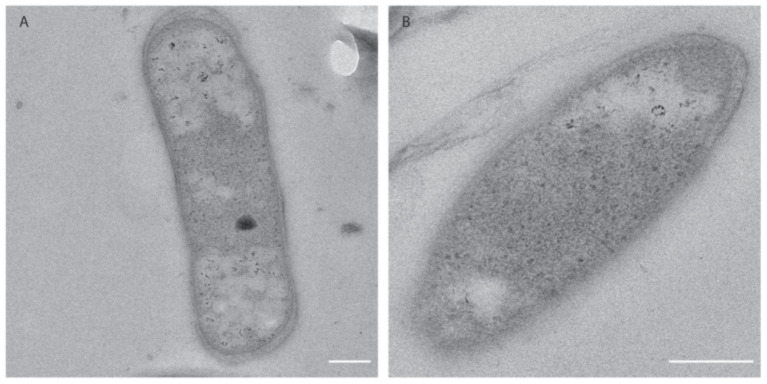
Electron micrograms of *M. thermophilum* AP8 cells. **(A)** Rod shaped cells contained ribosomes (black dots), electron-dense particles (black circle), and electron light particles (white particles). Scale bar: 200 nm. Magnification: 20,000×. **(B)** Cell of *M. thermophilum* AP8 at higher magnification. Scale bar: 200 nm. Magnification: 40,000×. Membrane stacks and electron dense particles are not visible.

To further characterize *M. thermophilum* AP8, the cell morphology was investigated *via* electron microscopy (Figure 5). The cells appeared to be rod-shaped and no S-layer could be observed. An outer membrane and an inner membrane were visible, classifying this bacterium as Gram negative. The cytoplasm of the cells did not show the presence of membrane stacks like in proteobacterial methanotrophs but contained ribosomes and both electron light (EL) and electron dense (ED) particles. The ED particles were occasionally found in the cells, whereas EL particles were found in most cells and were frequently located at the poles (Figures 5A,B).

### *pmoCAB2* and *xoxF1* Are Expressed at Maximum Growth Rate

**Figure 6:**
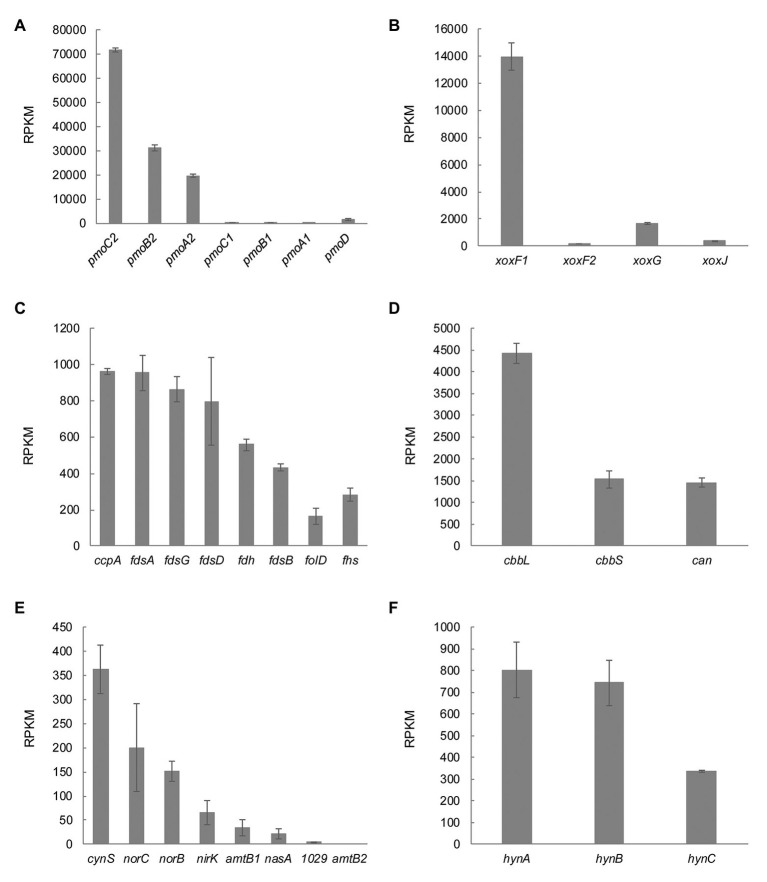
Transcriptomic profile of genes involved in carbon, nitrogen and hydrogen metabolism. **(A)** The particulate methane monooxygenase is encoded by two different operons in *M. thermophilum* AP8. At maximum growth rate, *pmoCAB2* is expressed. **(B)** Expression of the lanthanide dependent methanol dehydrogenase genes *xoxF1* and *xoxF2* and of the genes encoding the cytochrome *c* (*xoxG*) and the periplasmic substrate binding protein (*xoxJ*). **(C)** *M. thermophilum* AP8 encodes for different formate dehydrogenases and for genes involved in the tetrahydrofolate pathway (*folD,* Methylenetetrahydrofolate dehydrogenase/Methenyltetrahydrofolate cyclohydrolase; *fhs,* Formate-tetrahydrofolate ligase). **(D)** *cbbL* and *cbbS* correspond to genes encoding the large and small subunit of the Ribulose-1,5-bisphosphate carboxylase oxygenase (RuBisCO); *can*, carbonic anhydrase. **(E)** *cynS*, cyanate hydratase; *norC*, nitric oxide reductase subunit C; *norB*, nitric oxide reductase subunit B; *nirK*, nitrite reductase; *amtb1* and *amtb2*, ammonium transporters; *nasA*, nitrate transporter; 1029, MTHMO_v1_1029, assimilatory nitrate reductase/nitrite reductase. **(F)** Expression of the three subunits of the type 1b [NiFe] hydrogenase. A list of all genes with relative RPKM values can be found in Supplementary Table S4.

Genome analysis showed presence of multiple copies of genes involved in methane metabolism (see above). To study their differential expression in *M. thermophilum* AP8, mRNA extracted from cells grown at μ_max_ in the chemostat was sequenced and analyzed. The data showed that the oxidation of methane was carried out by the pMMO encoded by operon *pmoCAB2* (MTHMO_v1_0896-0898). The second operon, *pmoCAB1* (MTHMO_v1_0893-0895), was hardly expressed (Figure 6A). Similarly, XoxF1 (MTHMO_v1_1700) was the main enzyme responsible for methanol oxidation, since the *xoxF2* (MTHMO_v1_1756) expression was 85-fold lower (Figure 6B). The cytochrome encoded by *xoxG* (MTHMO_v1_1702) had a higher expression value compared to *xoxJ* (MTHMO_v1_1701; Figure 6B). Furthermore, the transcriptome was analyzed for genes that might be involved in lanthanide uptake, according to the experiments performed in *M. extorquens* PA1 (Ochsner et al., 2019). Most of these genes could not be found in the genome of strain AP8, but MTHMO_v1_2099, encoding for an ABC type transporter, and the TonB dependent receptor *cirA* (MTHMO_v1_1697), exhibited the highest expression values among the other putative lanthanide transporters (Supplementary Table S3).

The formate produced during methanol oxidation needs to be converted to CO_2_, which can be assimilated in the CBB cycle by a type I RuBisCO. Formate dehydrogenases (MTHMO_v1_1065-1068, 0868), genes involved in the tetrahydrofolate pathway (*folD*, MTHMO_v1_0721 and *fhs*, MTHMO_v1_2088), as well as carbonic anhydrase (*can*, MTHMO_v1_0262) and the ribulose-1,5-bisphosphate carboxylase oxygenase (RuBisCO) encoding genes (*cbbL* and *cbbS*, MTHMO_v1_1810, 1811) were expressed (Figures 6C,D).

The nitrogen metabolism was also analyzed in detail and it showed that cyanate hydratase (*cynS*, MTHMO_v1_1705) was constitutively expressed even if cyanate was not supplied in the medium. The genes responsible for NO (*norCB*, MTHMO_v1_1794, 1795) and NO_2_^−^ detoxification (*nirK*, MTHMO_v1_0885) had expression levels between 200 and 65 RPKM. Furthermore, the NH_4_^+^ and NO_3_^−^ transporters (*amtB*, MTHMO_v1_0690, 0691 and *nasA* MTHMO_v1_1025) did not have high levels of expression (<50 RPKM) and the assimilatory nitrate reductase/nitrite reductase (MTHMO_v1_1031) was basically not expressed (Figure 6E). Moreover, the genes encoding a nitrogenase showed expression below 76 RPKM (Supplementary Table S4). These results indicate that, in the tested growth conditions, strain AP8 might detoxify NO_2_^−^ and NO *via* NirK and NorCB, but not reduce NO_2_^−^ since ammonia is already largely available to the cells.

Under high ammonia concentrations, NH_4_^+^ incorporation into biomass usually happens *via* the glutamate dehydrogenase (GDH) rather than *via* glutamine synthetase/glutamate synthase (GS-GOGAT) pathway (Tyler, 1978; Bellion and Bolbot, 1983). Surprisingly, the transcriptome data of strain AP8 showed that GS-GOGAT had higher expression values than GDH (1,249 and 275 RPKM vs. 150 RPKM).

The *M. thermophilum* AP8 genome also contained genes encoding a type 1b [NiFe] hydrogenase. Despite H_2_ not being supplied to the cell, the genes *hynABC* (MTHMO_v1_1378-1380) and their maturation factors had expression values up to 800 RPKM (Figure 6F; Supplementary Table S5). To investigate if this type of hydrogenase was actively metabolizing hydrogen in *M. thermophilum* AP8, the H_2_ metabolism was studied in more details.

### Type 1b [NiFe] Hydrogenase Supports H_2_ Consumption in Microaerobic Conditions

**Figure 7:**
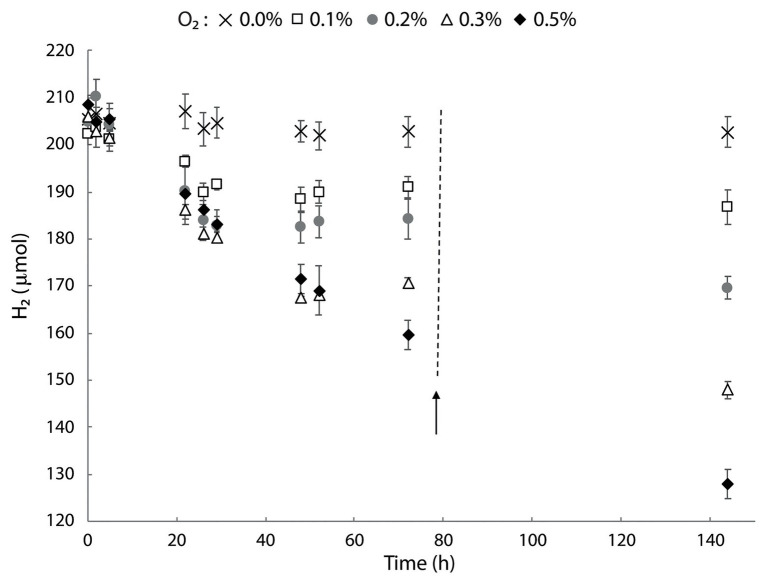
Hydrogen consumption in *M. thermophilum* AP8 with different O_2_ concentrations. Bottles were incubated with 0–0.5% O_2_ and the activity followed for 144 h. The black arrow indicates the addition of O_2_ to the initial concentration. Error bars represent the standard deviation over the average from three biological replicates.

The genome and transcriptome of *M. thermophilum* AP8 revealed the presence of a type 1b [NiFe] hydrogenase that was classified as a membrane bound, O_2_-sensitive, and H_2_-uptake hydrogenase (Søndergaard et al., 2016). We studied the activity of this enzyme using cells of *M. thermophilum* AP8 pre-grown with CH_4_ as energy source and with 21% O_2_. These cells were subsequently transferred in bottles containing 200 μmol of H_2._ In a preliminary experiment with oxygen concentration ranging from 0.2 to 5%, we observed hardly any H_2_ consumption above 1% O_2_ (Supplementary Figure S6). In a second experiment, we could show that hydrogen oxidation was observed within 2 h in bottles containing 0.1–0.5% O_2_. No consumption was detected in absence of O_2_ (Figure 7). Decrease of H_2_ continued until O_2_ became limiting and it restarted after additional O_2_ was supplied. During these experiments, a slight increase in biomass could be observed (data not shown).

These results showed that the type 1b [NiFe] hydrogenase encoded by *M. thermophilum* AP8 was not strictly O_2_ sensitive but it required very low concentrations of O_2_ (<1%) in order to actively oxidize H_2_.

## Discussion

This study reports the isolation and characterization of a novel bacterial strain, tentatively named *M. thermophilum* AP8. Strain AP8 was discovered in a soil sample from the geothermal area Favara Grande on Pantelleria Island. This area is characterized by low pH and high temperatures (Gagliano et al., 2016). Phylogenetic analysis showed that this bacterium was part of the phylum Verrucomicrobia that includes mesophilic and thermoacidophilic methanotrophs found in volcanic ecosystems (Dunfield et al., 2007; Pol et al., 2007; Islam et al., 2008; Sharp et al., 2014; van Teeseling et al., 2014).

The genus *Methylacidimicrobium* comprises mesophilic strains that grow optimally between 30 and 44°C and are extremely acid-tolerant, showing growth at pH as low as 0.5 (Sharp et al., 2014; van Teeseling et al., 2014). *Methylacidimicrobium thermophilum* AP8 was not able to grow at pH below 1.5 but it exhibited a higher temperature optimum (50°C) and a faster growth rate (0.05 h^−1^ vs. 0.01–0.04 h^−1^) compared to other *Methylacidimicrobium* species. In this regard, *M. thermophilum* AP8 represents the first thermophilic strain of the genus *Methylacidimicrobium*.

The morphology of this bacterium was similar to the other verrucomicrobial methanotrophs. Cells were Gram negative, rod-shaped and their cytoplasm contained ribosomes, putative glycogen storage and polyphosphate particles (Khadem et al., 2012b; van Teeseling et al., 2014). Comparably to *M. tartarophylax* 4AC and *M. cyclopophantes* 3B, membrane stacks were not detected, which questions the location of the pMMO enzyme. The only exception is represented by *M. fagopyrum* 3C, whose membrane stacks are visible in the cytoplasm (van Teeseling et al., 2014).

Analysis of the genome revealed the presence of two copies of the operon encoding pMMO, but only the *pmoCAB2* genes showed high expression levels. In *Methylocystis* sp. SC2 the different pMMO’s had different affinities for CH_4_ [<1 μM (PmoCAB2) vs. 9 μM (PmoCAB1)], suggesting that differential expression of *pmoCAB* might depend on the concentration of methane (Baani and Liesack, 2008). Experiments performed in *M. fumariolicum* SolV, instead, have demonstrated that two of the three different *pmoCAB* operons were subjected to oxygen-dependent regulation (Khadem et al., 2012a).

*Methylobacterium thermophilum* AP8 showed an affinity constant for CH_4_ (K_S(app)_) of 8 ± 1 μM, which is close to that reported for the thermophilic *M. fumariolicum* SolV (6 μM; Pol et al., 2007). These values are similar to those found for proteobacterial methanotrophs (K_s(app)_ 1–12 μM; Joergensen and Degn, 1987; Bender and Conrad, 1992; Knief and Dunfield, 2005; Baani and Liesack, 2008). Interestingly, the newly isolated atmospheric methane oxidizer *Methylocapsa gorgona* MG08 was shown to have K_S(app)_ of 5 μM and still be able to grow on atmospheric methane concentrations (Tveit et al., 2019).

The genome of *M. thermophilum* AP8 encoded two copies of MDH and transcriptome analysis showed that XoxF1 had 85-fold higher expression compared to XoxF2. Biochemical data demonstrated that the type of lanthanide available may influence the activity of (different) XoxF-type MDHs. XoxF from *Methylomonas* sp. LW13 and *Bradyrhizobium* sp. MAFF211645 functioned with lanthanides from La^3+^ to Nd^3+^ (atomic number 57–60), whereas in *Methylotenera mobilis,* activity was detected with REEs until atomic number 64 (La^3+^–Gd^3+^; Fitriyanto et al., 2011; Huang et al., 2018). Strain AP8 was cultivated with either Nd^3+^ or Ce^3+^ which are both considered light lanthanides. It remains to be tested if the addition of a heavier lanthanide would lead to the expression of XoxF2.

To date, the mechanism behind REEs incorporation in bacterial cells is not completely clarified. The protein Lanmodulin was described to be able to bind lanthanides in the methylotrophic bacterium *M. extorquens* AM1 (Cotruvo et al., 2018) but no homolog could be detected in strain AP8. In *M. extorquens* PA1, proteomics analysis described genes involved in growth with REE (Ochsner et al., 2019) but when *M. thermophilum* AP8 was analyzed for the presence and expression of these genes (Supplementary Table S3), most of them could not be found. Among the retrieved homologues, a TonB dependent receptor (*cirA*, MTHMO_v1_1697) and a component of the ABC transport system (MTHMO_v1_2099) showed expression of 392 and 587 RPMK, respectively. These genes are highly conserved in verrucomicrobial methanotrophs and probably responsible for REEs uptake. MTHMO_v1_2099 was located next to the gene MTHMO_v1_2097, a putative copper binding protein (248 RPKM), and MTHMO_v1_2098 that encoded a transport permease protein (157 RPKM; Supplementary Table S4). These observations indicate that the mechanism of lanthanides uptake in verrucomicrobial methanotrophs is different compared to proteobacterial methylotrophs.

The lanthanide-dependent MDH XoxF can produce formaldehyde and/or formate depending on the bacterial strain (Pol et al., 2014; Good et al., 2018). In the *M. fumariolicum* SolV, purified XoxF produced formate in a four-electron process (Pol et al., 2014). Strain AP8 does not encode a formaldehyde dehydrogenase (EC 1.2.1.46), but genes responsible for the tetrahydrofolate (H_4_F)-dependent formaldehyde oxidation were detected and their expression found to be >160 RPKM (Figure 6C; Supplementary Table S4). This pathway is likely to be involved in the conversion of toxic formaldehyde to prevent unspecific reactions with proteins and nucleic acids (Vorholt, 2002). Formaldehyde is also produced during the reaction catalyzed by the enzyme methanethiol oxidase (MTHMO_v1_1990), which appeared to be continuously expressed at a level of 230 RPKM (Supplementary Table S4). Interestingly, a homolog of this gene has been found in humans and its mutation linked to oral halitosis (Pol et al., 2018).

*Methylobacterium thermophilum* AP8 showed the presence of a type 1b [NiFe] hydrogenase. These hydrogenases are usually encoded by anaerobic microorganisms, they are O_2_ sensitive and found in hypoxic soil, like permafrost (Greening et al., 2016). In strain AP8, H_2_ oxidation was observed when headspace O_2_ concentrations were below 1%, which corresponds to ~9 μM of dissolved oxygen at 50°C. Similar results were obtained in *M. tartarophylax* 4 AC, which also only encoded a type 1b [NiFe] hydrogenase and oxidized H_2_ at higher rate when O_2_ was lower than 10 μM, but it could show activity until below 40 μM O_2_ (Mohammadi et al., 2019). Transcriptome analysis revealed that the [NiFe] hydrogenase was expressed even when no H_2_ was supplied to the culture. A constitutive expression of the enzyme hydrogenase was also observed in the thermophilic bacterium *M. fumariolicum* SolV when grown on methane. Strain SolV does not encode a type 1b but instead more oxygen tolerant groups 1d and 1 h/5 hydrogenases, that appear to be regulated by O_2_ (Mohammadi et al., 2017a). The same is the case for *Methylacidiphilum* sp. RTK17.1 for which a 3.5-fold upregulation of the group 1d [NiFe] hydrogenase was reported in response to oxygen limitation (Carere et al., 2017). These data suggest that verrucomicrobial methanotrophs in volcanic environments could use hydrogen as an alternative energy source and they possess different types of hydrogenases, regulated by the spectrum of oxygen concentrations (Carere et al., 2019; Schmitz et al., 2020).

### Conclusion

In conclusion, this study reports the discovery of a novel species of methanotrophic Verrucomicrobium, *M. thermophilum* AP8. This bacterium could grow as methanotroph optimally at 50°C in a pH range of 3–5, representing the first thermophilic member of the genus *Methylacidimicrobium*. The properties of strain AP8 indicates that this bacterium occupies a distinct environmental niche compared to other *Methylacidimicrobium* species. The ability of oxidizing methane and hydrogen makes *M. thermophilum* AP8 particularly adapted to volcanic environments, where oscillation in oxygen concentrations and substrate availability might occur. The presence of a type 1b [NiFe] hydrogenase allowed active H_2_ consumption when O_2_ was below 10 μM. This observation supports previous studies that report the capacity of mixotrophic and/or “Knallgas” growth of verrucomicrobial methanotrophs. Furthermore, it shows that type 1b [NiFe] hydrogenases are not only involved in anaerobic respiration but also support H_2_ consumption under microaerobic conditions.

### Description of *Methylacidimicrobium thermophilum* sp. nov.

*Methylacidimicrobium thermophilum* (ther. mo. phil’ um; Gr. n. *thermus* heat; L. adj. *Philum* loving; L. n. *thermophilum* heat-loving) was isolated from volcanic soil of the Favara Grande area on Pantelleria Island, Italy. Cells are rod-shaped, Gram-negative, with glycogen and phosphate particles present in many cells. Growth occurred between pH 1.5–5.5 and 30–55°C. Optimum was registered at pH from 3 to 5 and 50°C. Doubling time at maximum growth rate was about 14 h. K_S(app)_ for methane was calculated at 8 ± 1 μM CH_4_. Hydrogen oxidation was observed below 10 μM O_2_.

## Data Availability Statement

The datasets presented in this study can be found in online repositories. The names of the repository/repositories and accession number(s) can be found below: https://www.ncbi.nlm.nih.gov/genbank/, PRJEB37308 https://www.ncbi.nlm.nih.gov/genbank/, LR797830.

## Author Contributions

NP, AP, MJ, and HC designed the projects and experiments. NP, AP, CH, AG, WD’A, PQ, and HC sampled the geothermal soils. NP and CH performed the enrichment and isolation experiments. NP, PB, and AW conducted the physiology experiments. RM performed the electron microscopic analyses. GC and PB sequenced the genome and transcriptome, reconstructed the genome, and analyzed the gene expression. NP, PB, AP, and HC carried out the data analysis. NP, PB, and HC wrote the manuscript. All authors contributed to revision of the manuscript, and read and approved the submitted version.

### Conflict of Interest

The authors declare that the research was conducted in the absence of any commercial or financial relationships that could be construed as a potential conflict of interest.
