# Supplementary material for: Methylacidimicrobium thermophilum AP8, a Novel Methane- and Hydrogen-Oxidizing Bacterium Isolated From Volcanic Soil on Pantelleria Island, Italy
Source: Front Microbiol. 2021 Feb 12;12:637762. doi: 10.3389/fmicb.2021.637762 (PMC7907005; doi:10.3389/fmicb.2021.637762)
Supplement: Supplementary file 1 [file Data_Sheet_1.pdf]

Supplementary Material

Methylacidimicrobium thermophilum AP8, a novel methane- and hydrogen-oxidizing bacterium isolated from volcanic soil on Pantelleria Island, Italy

Nunzia Picone[1], Pieter Blom[1], Anna J. Wallenius[1], Carmen Hogendoorn[1], Rob Mesman[1], Geert Cremers[1], Antonina L. Gagliano[2], Walter D'Alessandro[2], Paola Quatrini[3], Mike S. M. Jetten[1], Arjan Pol[1] and Huub J. M. Op den Camp[1*]

[1]Department of Microbiology, Institute for Water and Wetland Research, Radboud University, Nijmegen, the Netherlands

[2]Istituto Nazionale di Geofisica e Vulcanologia (INGV), Palermo, Italy

[3]Department of Biological, Chemical and Pharmaceutical Sciences and Technologies (STEBICEF), University of Palermo, Palermo, Italy

*** Correspondence: h.opdencamp@science.ru.nl**

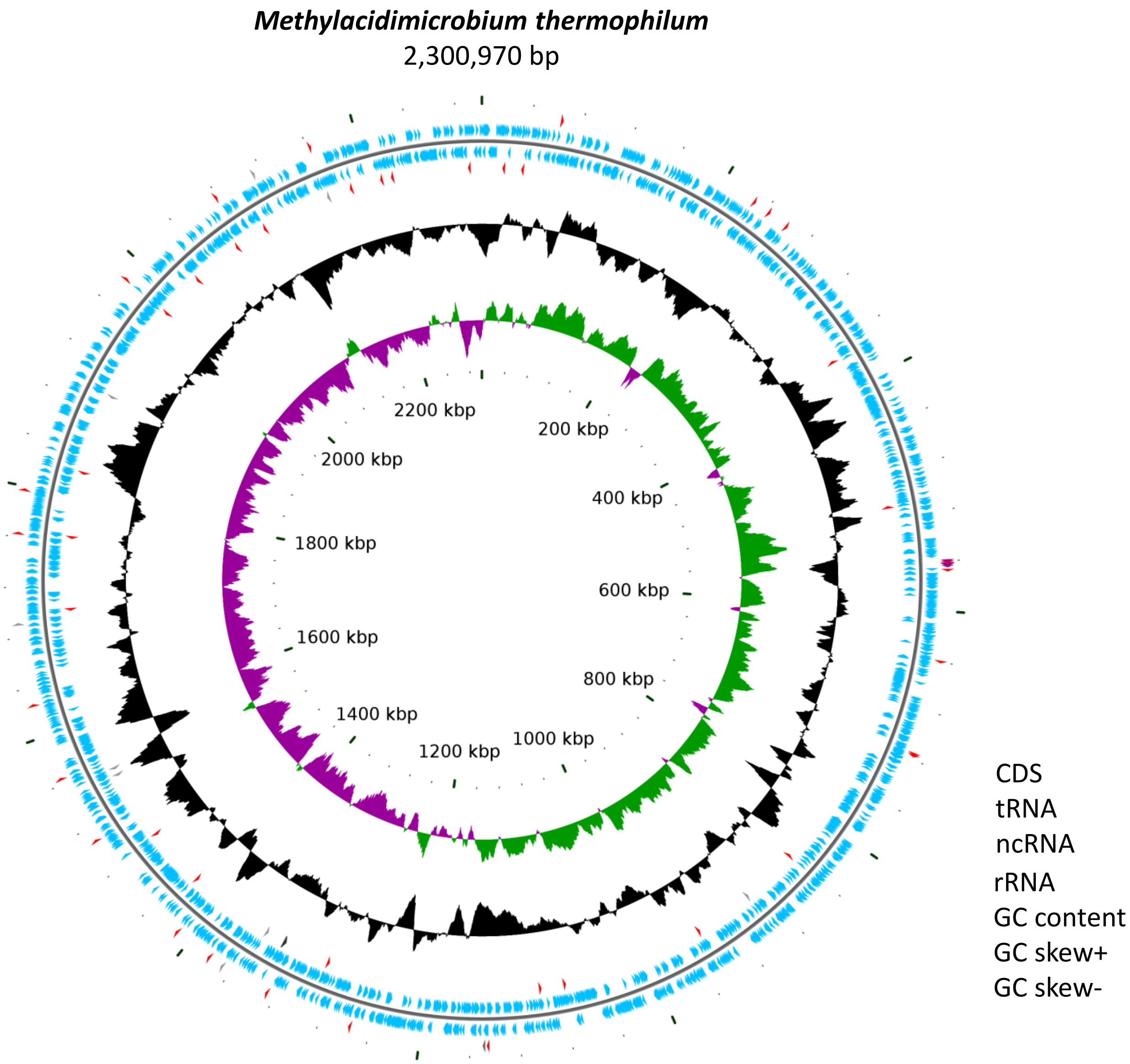

Supplementary Figure S1. Circular genome map of the full genome of *Methylacidmicrobium thermophilum* AP8.

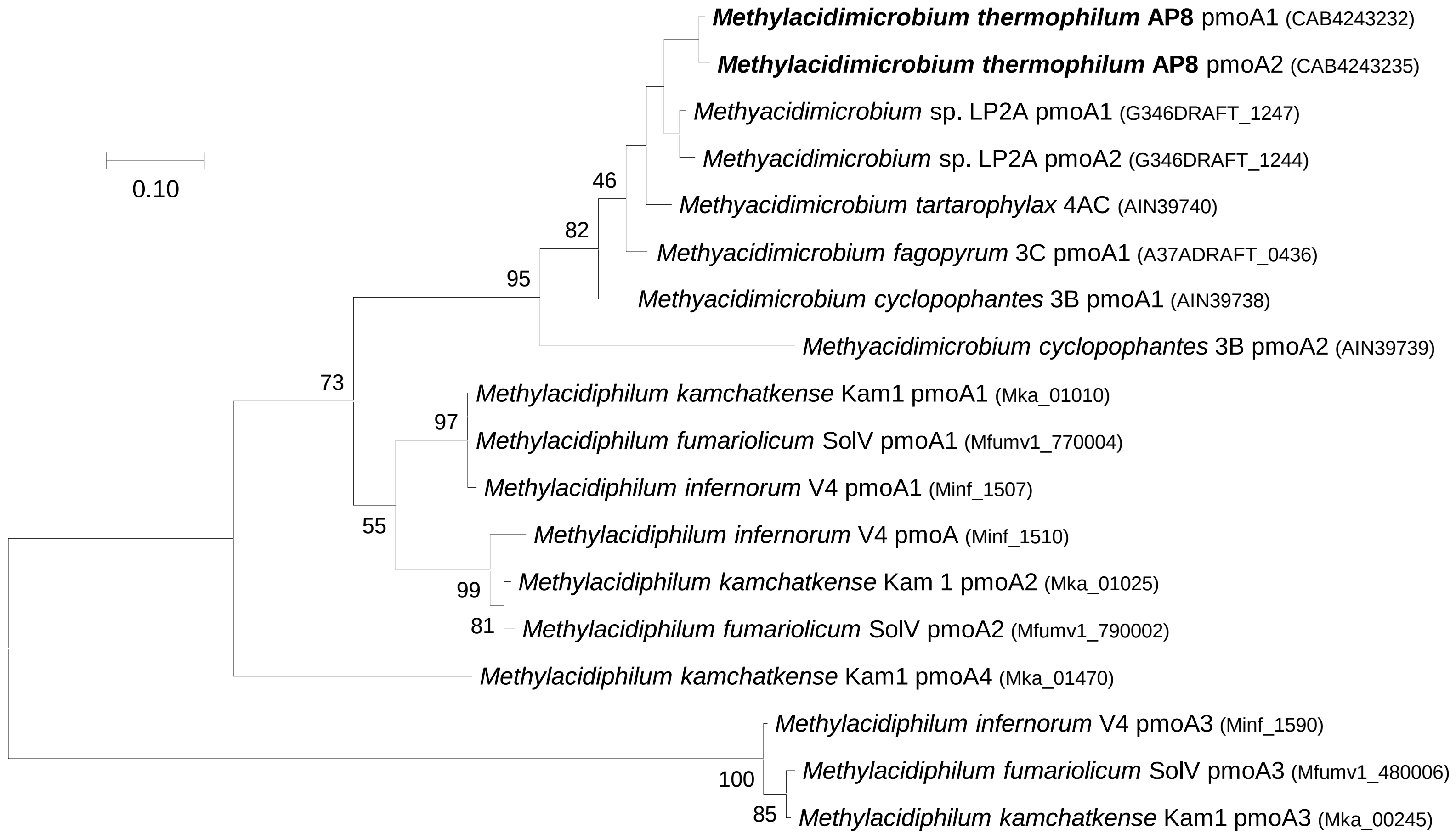

Supplementary Figure S2. Phylogenetic analysis of pMMOA subunits of the methane monoxygenase of cultured *Methylacidmicrobium* and *Methylacidiohilum* species.

The evolutionary history was inferred by using the Maximum Likelihood method based on the JTT matrix-based model. The tree with the highest log likelihood (-2842.88) is shown. The percentage of trees in which the associated taxa clustered together is shown next to the branches. Initial tree(s) for the heuristic search were obtained automatically by applying Neighbor-Join and BioNJ algorithms to a matrix of pairwise distances estimated using a JTT model, and then selecting the topology with superior log likelihood value. The tree is drawn to scale, with branch lengths measured in the number of substitutions per site. The analysis involved 18 amino acid sequences. All positions containing gaps and missing data were eliminated. There were a total of 243 positions in the final dataset. Evolutionary analyses were conducted in MEGA7 (Kumar et al., 2016). The deviating pMMOA3 sequences of the *Methylacidiphilum* species were used as an outgroup.

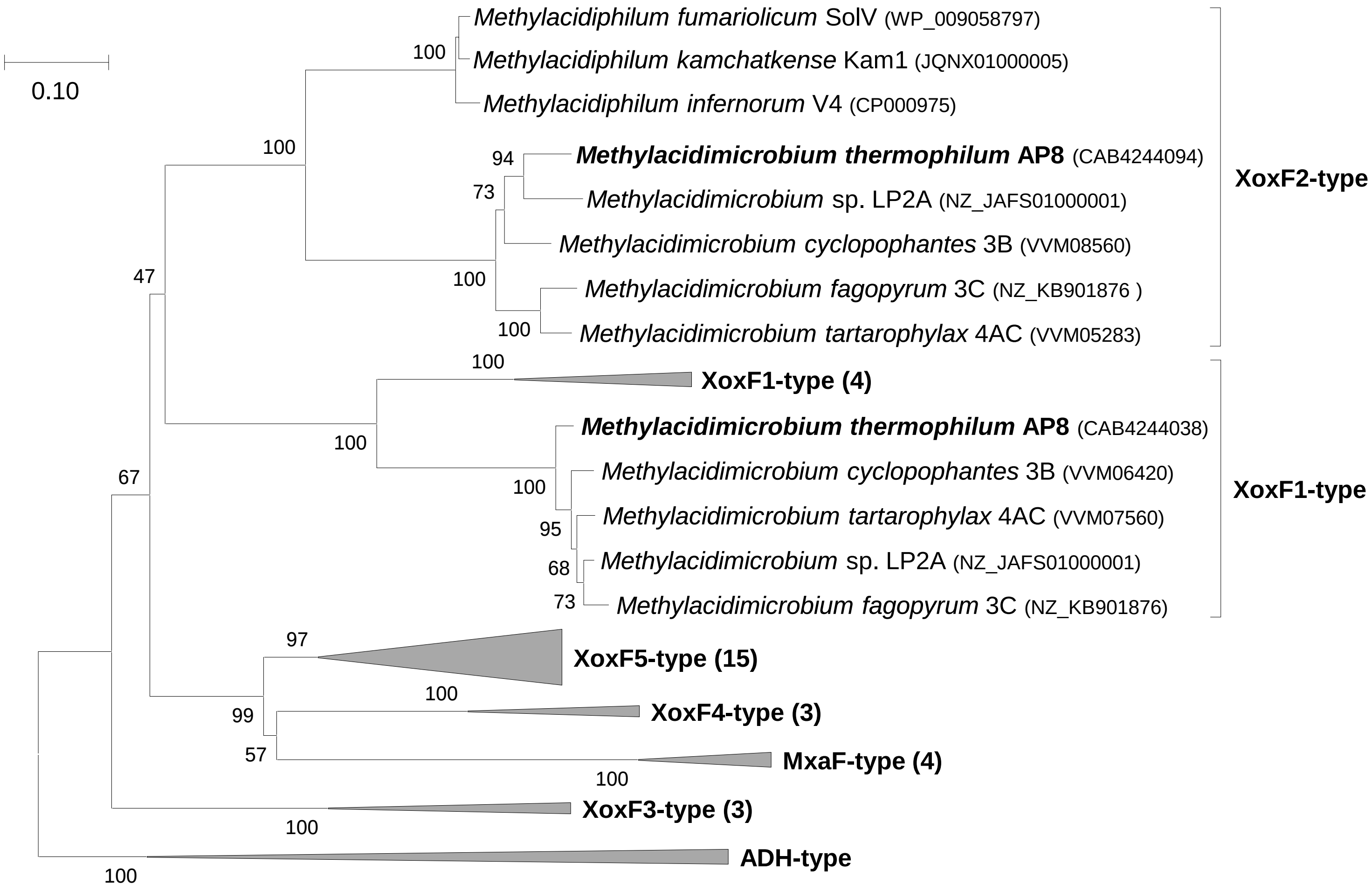

Supplementary Figure S3. Phylogenetic tree of methanol dehydrogenase.
The evolutionary history was inferred using the Neighbor-Joining method . The optimal tree with the sum of branch length = 7.82098608 is shown. The percentage of replicate trees in which the associated taxa clustered together in the bootstrap test (500 replicates) are shown next to the branches. The tree is drawn to scale, with branch lengths in the same units as those of the evolutionary distances used to infer the phylogenetic tree. The evolutionary distances were computed using the Dayhoff matrix based method and are in the units of the number of amino acid substitutions per site. The analysis involved 47 amino acid sequences. All positions containing gaps and missing data were eliminated. There were a total of 487 positions in the final dataset. Alcohol dehydrogenases of the ADH-type were used an an outgroup. Evolutionary analyses were conducted in MEGA7 (Kumar et al., 2016).

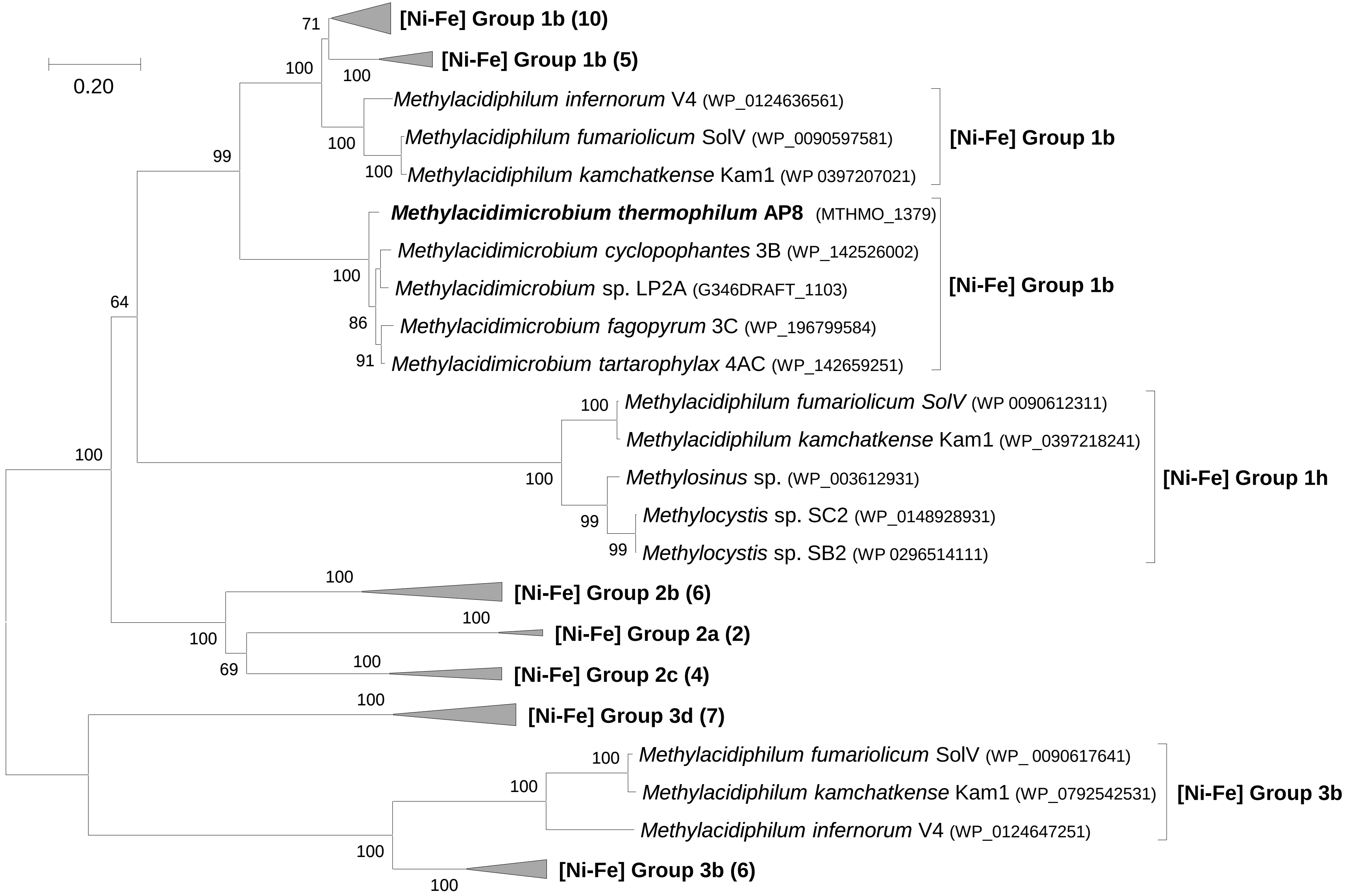

Supplementary Figure S4. Phylogenetic analysis of the large subunits of [Ni-Fe] hydrogenase.
The evolutionary history was inferred using the Neighbor-Joining method The optimal tree with the sum of branch length = 10.46552257 is shown. The percentage of replicate trees in which the associated taxa clustered together in the bootstrap test (500 replicates) are shown next to the branches. The tree is drawn to scale, with branch lengths in the same units as those of the evolutionary distances used to infer the phylogenetic tree. The evolutionary distances were computed using the Dayhoff matrix based method and are in the units of the number of amino acid substitutions per site. The analysis involved 56 amino acid sequences. All positions containing gaps and missing data were eliminated. There were a total of 347 positions in the final dataset. Classification was according to the hydDB database (https://services.birc.au.dk/hyddb/). Evolutionary analyses were conducted in MEGA7 (Kumar et al., 2016).

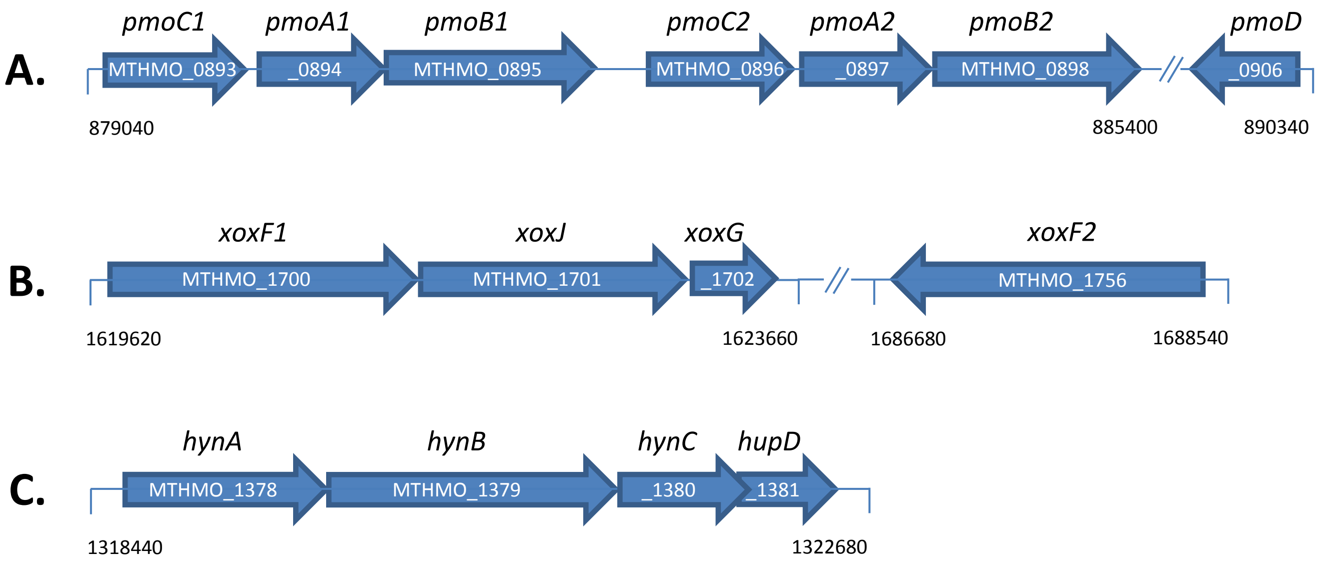

Supplementary Figure S5. Genetic organization of the key enzymes involved in methane, methanol and hydrogen consumption in *Methylacidmicrobium thermophilum* AP8.
A. Genes encoding methane monooxygenase; **B.** Genes involved in methanol conversion; **C.** [Ni-Fe[group 1b hydrogenase gene cluster. Numbers indicate the position in the genome.

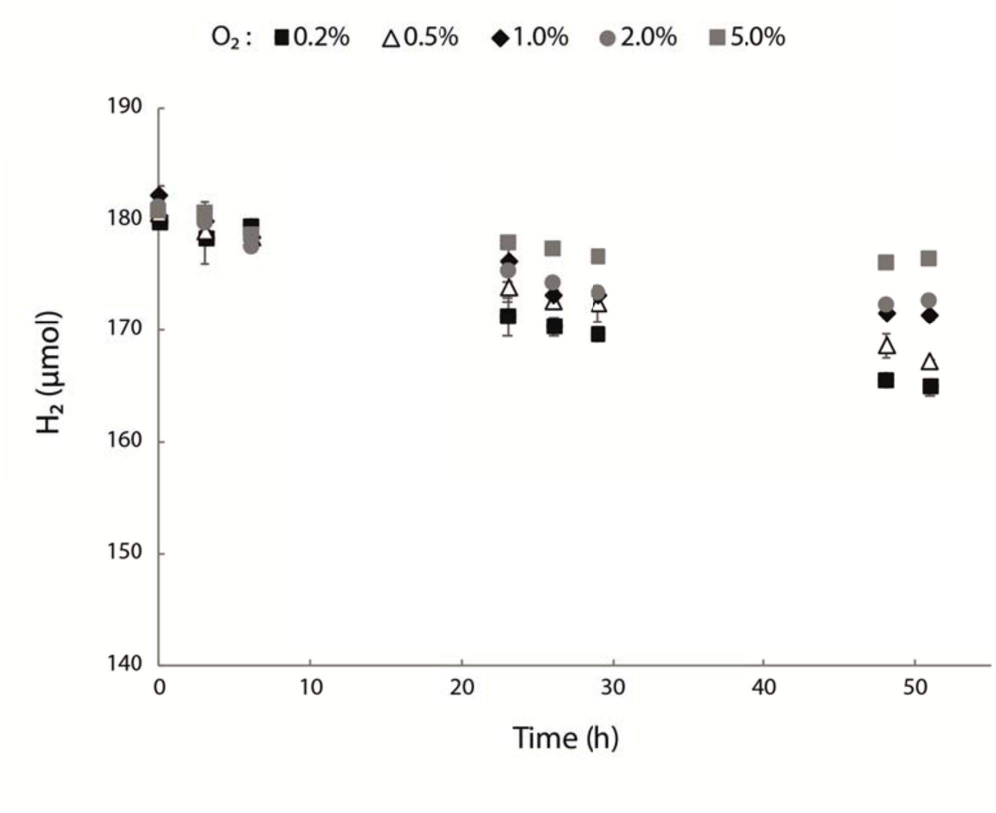

Supplementary Figure S6 | Hydrogen consumption in *M. thermophilum* AP8 with different O_2 concentrations. Cultures were incubated with H_2 and concentrations of O_2 ranging from 0.2 to 5%. The consumption of H_2 was monitored over 50 h. No activity was detected above 1% O_2. Error bars represent the standard deviation over the average from three biological replicates.

Supplementary Table S1. Average Nucleotide Identity (ANI) values express in % between *Methylacidimicrobium thermophilum* AP8 (1), *Methylacidimicrobium fagopyrum* 3C (2), *Methylacidimicrobium tartarophylax* 4AC (3), *Methylacidimicrobium cyclopophantes* 3B (4), *Methylacidimicrobium* sp. L2PA (5).

		**1**	**2**	**3**	**4**	**5**
**1**	*Methylacidimicrobium thermophilum* AP8					
**2**	*Methylacidimicrobium fagopyrum* 3C	80.6				
**3**	*Methylacidimicrobium tartarophylax* 4AC	79.3	87.1			
**4**	*Methylacidimicrobium cyclopophantes* 3B	77.7	78.7	78.9		
**5**	*Methylacidimicrobium* sp. LP2A	80.6	80.1	79.7	78.6	

Table S2. Proteins encoded in the genome of *Methylacidimicrobium thermophilum* AP8 with predicted functions.

**Methane metabolism**						
	**Methane oxidation**					
**Gene**	**Product**	**EC no.**	**CDS[a]**	**Best BLAST hit in SwissProt[b]**	**Best BLAST hit in TrEMBL[c]**	**Notes**
pmoC1	Particulate methane monooxygenase subunit C	1.14.13.25	MTHMO_v1_0893	Mus musculus (Q8BG16, 25.83%)	Methylacidiphilum sp. RTK17.1 (A0A1W5LCU6, 70.55%)	
pmoA1	Particulate methane monooxygenase subunit A	1.14.18.3	MTHMO_v1_0894	Methylococcus capsulatus strain ATCC 33009 / NCIMB 11132 / Bath (Q607G3, 52.54%)	Methylacidimicrobium tartarophylax (A0A088MKB6, 90.91%)	beta subunit
pmoB1	Particulate methane monooxygenase subunit B	1.14.18.3	MTHMO_v1_0895	Methylococcus capsulatus strain ATCC 33009 / NCIMB 11132 / Bath (G1UBD1, 39.05%)	Methylacidiphilum sp. RTK17.1 (A0A1W5LDQ9, 62.19%)	alpha subunit
pmoC2	Particulate methane monooxygenase subunit C	1.14.13.25	MTHMO_v1_0896	Mus musculus (Q8BG16, 25.83%)	Methylacidiphilum sp. RTK17.1 (A0A1W5LCU6, 70.18%)	
pmoA2	Particulate methane monooxygenase subunit A	1.14.18.3	MTHMO_v1_0897	Methylococcus capsulatus strain ATCC 33009 / NCIMB 11132 / Bath (Q607G3, 52.12%)	Methylacidimicrobium tartarophylax (A0A088MKB6, 90.51%)	beta subunit
pmoB2	Particulate methane monooxygenase subunit B	1.14.18.3	MTHMO_v1_0898	Methylococcus capsulatus strain ATCC 33009 / NCIMB 11132 / Bath (G1UBD1, 39.05%)	Methylacidiphilum sp. RTK17.1 (A0A1W5LDQ9, 62.19%)	alpha subunit
pmoD	Conserved exported protein of unknown function, putative particulate methane monooxygenase subunit D		MTHMO_v1_0906	Mycobacterium tuberculosis strain ATCC 25618 / H37Rv (Q79FW5, 32.84%)	Methylacidiphilum kamchatkense Kam1 (A0A0C1V6J1, 45.25%)	
xoxF2	Methanol dehydrogenase	1.1.2.7	MTHMO_v1_1756	Paracoccus denitrificans (P29968, 46.76%)	Methylacidiphilum fumariolicum (D2D568, 66.78%)	

xoxF1	Methanol dehydrogenase	1.1.2.7	MTHMO_v1_1700	Paracoccus denitrificans (P29968, 45.98%)	Xanthomonas sp. NCPPB 1128 (A0A0J6G0G8, 62.6%)	
xoxJ	Periplasmic substrate binding protein		MTHMO_v1_1701	Mus musculus (Q8BZ25, 37.25%)	Pseudoxanthomonas sp. GM95 (A0A1H7QLX7, 47.95%)	Sec signal peptide predicted. Most likely cleavage site: 1 - 30 [ALS-GQ]
xoxG	Cytochrome c-L		MTHMO_v1_1702	Paracoccus denitrificans (P29899, 28.7%)	Bacterium HR13 (A0A2H5WGC5, 68.81%)	
pqqE	Coenzyme PQQ synthesis protein E		MTHMO_v1_1061	Granulibacter bethesdensis strain ATCC BAA-1260 / CGDNIH1 (Q0BQS8, 56.66%)	Methylacidiphilum fumariolicum strain SolV (I0JZV2, 62.57%)	
pqqD	Coenzyme PQQ synthesis protein D		MTHMO_v1_1062	Klebsiella pneumoniae (P27506, 47.69%)	Methylacidiphilum sp. RTK17.1 (A0A1W5LCV4, 54.22%)	
pqqC	Pyrroloquinoline-quinone (PQQ) synthase	1.3.3.11	MTHMO_v1_1063	Pseudomonas aeruginosa strain LESB58 (B7VAB8, 63.51%)	Methylacidiphilum infernorum (A9QPG2, 67.89%)	
pqqB	Coenzyme PQQ synthesis protein B		MTHMO_v1_1064	Methylococcus capsulatus strain ATCC 33009 / NCIMB 11132 / Bath (Q608P3, 42.16%)	Methylacidiphilum fumariolicum strain SolV (I0JZV5, 50.51%%)	
pqqG	putative coenzyme PQQ synthesis protein G		MTHMO_v1_1964	Porphyromonas gingivalis strain ATCC BAA-308 / W83 (Q7MUY1, 37.31%)	Methylacidiphilum infernorum (A9QPC2, 58.37%)	
cirA	Putative TonB-dependent receptor		MTHMO_v1_1697	Escherichia coli (Q93SE0, 21.8%)	Methylacidiphilum fumariolicum SolV (I0JVH7, 73.46%)	
fdh	Formate dehydrogenase	1.17.1.9	MTHMO_v1_0868	Pseudomonas sp. strain 101 (P33160, 77.69%)	Chloroflexi bacterium CSP1-4 (A0A0T5ZJ23, 83.46%)	
fdsA	Formate dehydrogenase alpha subunit	1.2.1.2	MTHMO_v1_1067	Bacillus subtilis strain 168 (Q795Y4, 34.56%)	Methylacidiphilum kamchatkense Kam1 (A0A0C1UQ75, 73.43%)	molybdenum containing
fdsB	Formate dehydrogenase beta subunit	1.2.1.2	MTHMO_v1_1066	Desulfovibrio fructosivorans (Q46507, 47.68%)	Methylacidiphilum sp. RTK17.1 (A0A1W5LDT2, 69.53%)	
fdsG	Formate dehydrogenase	1.2.1.2	MTHMO_v1_1065	Desulfovibrio	Methylacidiphilum	NAD-dependent

	gamma subunit			fructosivorans (Q46505, 33.8%)	fumariolicum strain SolV (I0JZV7, 58.39%)	
fdsD	Formate dehydrogenase delta subunit	1.2.1.2	MTHMO_v1_1068	no hits	Methylacidiphilum kamchatkense Kam1 (A0A0C1RIW5, 55.22%)	
dhaS	Putative aldehyde dehydrogenase DhaS	1.2.1.3	MTHMO_v1_0462	Bacillus subtilis strain 168 (O34660, 59.55%)	Methylacidiphilum fumariolicum strain SolV (I0K090, 72.02%)	
fabG	Alcohol dehydrogenase	1.1.1.100	MTHMO_v1_2344	Staphylococcus aureus strain MW2 (P0A0I0, 36.1%)	Methylacidiphilum kamchatkense Kam1 (A0A0C1URM8, 62.34%)	
adhA	Putative alcohol dehydrogenase AdhA	1.1.1.1	MTHMO_v1_0360	Mycobacterium tuberculosis strain ATCC 25618 / H37Rv (P9WQC1, 50.4%)	Bordetella sp. H567 (A0A1B2R9L8, 69.05%)	
fabG	Short-chain alcohol dehydrogenase	1.1.1.100	MTHMO_v1_0512	Bacillus subtilis strain 168 (P14802, 34.84%)	Methylacidiphilum infernorum (B3E0L6, 56.96%)	
ccpA	Cytochrome c551 peroxidase (fragment)	1.11.1.5	MTHMO_v1_2002	Pseudomonas aeruginosa strain ATCC 15692 / DSM 22644 / CIP 104116 / JCM 14847 / LMG 12228 / 1C / PRS 101 / PAO1 (P14532, 52.94%)	Methylacidiphilum kamchatkense Kam1 (A0A0C1RIZ2, 71.09%)	Putative TTQ cofactor maturation protein for MtoX protein (methanethiol oxidase)
ccpA	Cytochrome c551 peroxidase (fragment)	1.11.1.5	MTHMO_v1_2003	Pseudomonas aeruginosa strain ATCC 15692 / DSM 22644 / CIP 104116 / JCM 14847 / LMG 12228 / 1C / PRS 101 / PAO1 (P14532, 54.26%)	Methylacidiphilum kamchatkense Kam1 (A0A0C1RIZ2, 72.82%)	Putative TTQ cofactor maturation protein for MtoX protein (methanethiol oxidase)
mtoX	Methanethiol oxidase	1.8.3.4	MTHMO_v1_1990	Xenopus tropicalis (Q569D5, 40.54%)	Chthonomonas calidirosea strain DSM 23976 / ICMP 18418 / T49 (S0EZ77, 65.52%)	
	**Tetrahydrofolate pathway**					
folD	Methylenetetrahydrofolate dehydrogenase / Methenyltetrahydrofolate cyclohydrolase	1.5.1.5, 3.5.4.9	MTHMO_v1_0721	Moorella thermoacetica strain ATCC 39073 / JCM 9320 (Q2RIB4, 51.9%)	Methylacidiphilum infernorum (A9QPI2, 58.64%)	

fhs	Formate--tetrahydrofolate ligase	6.3.4.3	MTHMO_v1_2088	Rhodobacter sphaeroides strain ATCC 17029 / ATH 2.4.9 (A3PM52, 58.41%)	Methylacidiphilum infernorum (A9QPK0, 60.54%)	

Energy metabolism						
	**Complex I**					
Gene	**Product**	**EC no.**	**CDS[a]**	**Best BLAST hit in SwissProt[b]**	**Best BLAST hit in TrEMBL[c]**	**Notes**
nuoA	NADH-quinone oxidoreductase subunit A	1.6.5.11	MTHMO_v1_2243	Raphanus sativus (P68159, 39.67%)	Methylacidiphilum kamchatkense Kam1 (A0A0C1RIM0, 66.67%)	
nuoB	NADH-quinone oxidoreductase subunit B	1.6.5.11	MTHMO_v1_1942	Methylacidiphilum infernorum (B3DXN5, 81.66%)	Methylacidiphilum sp. RTK17.1 (A0A1W5LD16, 81.66%)	
nuoC	NADH-quinone oxidoreductase subunit C	1.6.5.11	MTHMO_v1_1941	Geobacter uraniireducens strain Rf4 (A5G9B7, 48.61%)	Methylacidiphilum kamchatkense Kam1 (A0A0C1V5F4, 61.33%)	
nuoD	NADH-quinone oxidoreductase subunit D	1.6.5.11	MTHMO_v1_1940	Methylacidiphilum infernorum (B3DXN7, 77.31%)	Methylacidiphilum sp. RTK17.1 (A0A1W5LD33, 77.81%)	
nuoE	NADH-quinone oxidoreductase subunit E	1.6.5.11	MTHMO_v1_1939	Rattus norvegicus (P19234, 32.78%)	Methylacidiphilum sp. RTK17.1 (A0A1W5LD12, 58.71%)	
nuoF	NADH-quinone oxidoreductase subunit F	1.6.5.11	MTHMO_v1_1938	Rickettsia bellii strain RML369-C (Q1RHA0, 47.02%)	Methylacidiphilum kamchatkense Kam1 (A0A0C1RLP0, 79.56%)	
nuoG	NADH-quinone oxidoreductase subunit G	1.6.5.11	MTHMO_v1_1937	Rickettsia felis strain ATCC VR-1525 / URRWXCal2 (Q4UK22, 41.36%)	Methylacidiphilum kamchatkense Kam1 (A0A0C1UT41, 64.74%)	
nuoH	NADH-quinone oxidoreductase subunit H	1.6.5.11	MTHMO_v1_1936	Koribacter versatilis strain Ellin345 (Q1IS40, 45.6%)	Methylacidiphilum sp. RTK17.1 (A0A1W5LD25, 70.49%)	
nuoI	NADH-quinone oxidoreductase subunit I	1.6.5.11	MTHMO_v1_2283	Rhodothermus marinus (Q4QSC5, 44.97%)	Methylacidiphilum infernorum (B3DZT5, 72.25%)	
nuoJ	NADH-quinone oxidoreductase subunit J	1.6.5.11	MTHMO_v1_2282	Paracoccus denitrificans (P29922, 40.12%)	Methylacidiphilum kamchatkense Kam1 (A0A0C1RK43, 59.04%)	

nuoK	NADH-quinone oxidoreductase subunit K	1.6.5.11	MTHMO_v1_2281	Methylacidiphilum infernorum (B3DZT3, 74.23%)	Methylacidiphilum sp. RTK17.1 (A0A1W5LFG5, 74.23%)	
nuoL	NADH-quinone oxidoreductase subunit L	1.6.5.11	MTHMO_v1_2280	Zygnema circumcarinatum (Q32RH9, 38.57%)	Methylacidiphilum infernorum (B3DZT2, 65.71%)	
nuoM	NADH-quinone oxidoreductase subunit M	1.6.5.11	MTHMO_v1_2279	Rickettsia felis strain ATCC VR-1525 / URRWXCal2 (Q4UK26, 32.83%)	Methylacidiphilum fumariolicum strain SolV (I0K0W3, 67.36%)	
nuoN	NADH-quinone oxidoreductase subunit N	1.6.5.11	MTHMO_v1_2278	Methylacidiphilum infernorum (B3DZT0, 59.53%)	Methylacidiphilum sp. RTK17.1 (A0A1W5LCQ0, 59.75%)	
	**Complex II**					
sdhC	Succinate dehydrogenase cytochrome b subunit		MTHMO_v1_0309	no hits	Methylacidiphilum sp. RTK17.1 (A0A1W5LFS3, 49.54%)	
sdhB	Succinate dehydrogenase catalytic subunit	1.3.5.1	MTHMO_v1_0311	Wolinella succinogenes strain ATCC 29543 / DSM 1740 / LMG 7466 / NCTC 11488 / FDC 602W (P17596, 31.78%)	Methylacidiphilum infernorum (A9QPJ6, 65.61%)	
	**Complex III**					
	Quinol:cytochrome c oxidoreductase	1.10.2.2	MTHMO_v1_2053	Wolinella succinogenes strain ATCC 29543 / DSM 1740 / LMG 7466 / NCTC 11488 / FDC 602W (Q7MAR3, 41.67%)	Methylacidiphilum sp. RTK17.1 (A0A1W5LCR4, 57.66%0	quinone-binding subunit 2
	**Complex IV**					
cyoA1	Heme/copper-type cyto-chrome oxidase, subunit 2	1.9.3.1	MTHMO_v1_0682	Thermus thermophilus strain HB8 / ATCC 27634 / DSM 579 (Q5SJ80, 39.62%)	Methylacidiphilum infernorum (B3DUV7, 65.41%)	
cyoB1	Heme/copper-type cyto-chrome oxidase, subunit 1	1.9.3.1	MTHMO_v1_0683	Thermus thermophilus strain HB8 / ATCC 27634 /	Methylacidiphilum infernorum (B3DUV8,	

				DSM 579 (Q5SJ79, 42.7%)	72.12%)	
cyoB	Heme/copper-type cyto-chrome oxidase, subunit 1	1.9.3.1	MTHMO_v1_1892	Methylacidiphilum fumariolicum strain SolV (I0K0R8, 68.04%)	2.2.1.11, 4.1.2.13	
cyoA	Heme/copper-type cyto-chrome oxidase, subunit 2	1.9.3.1	MTHMO_v1_2042	Bradyrhizobium diazoefficiens strain JCM 10833 / IAM 13628 / NBRC 14792 / USDA 110 (P98053, 34.8%)	MTHMO_v1_2215	
cyoB	Heme/copper-type cyto-chrome oxidase, subunit 1	1.9.3.1	MTHMO_v1_2048	Bradyrhizobium diazoefficiens strain JCM 10833 / IAM 13628 / NBRC 14792 / USDA 110 (P98000, 42.88%)	Ignavibacterium album strain DSM 19864 / JCM 16511 / NBRC 101810 / Mat9-16 (I0ANS2, 62.75%)	
ccoO	Cbb3-type cytochrome oxidase, cytochrome c subunit		MTHMO_v1_2050	no hits	Methylacidiphilum kamchatkense Kam1 (A0A0C1UQE9, 49.73%)	
ccoN	putative Cytochrome c oxidase cbb3-type subunit 1		MTHMO_v1_2051	Bradyrhizobium diazoefficiens strain JCM 10833 / IAM 13628 / NBRC 14792 / USDA 110 (Q03073, 23.31%)	Methylacidiphilum kamchatkense Kam1 (A0A0C1UNA1, 37.81%)	
ctaB	Protoheme IX farnesyltransferase	2.5.1.-	MTHMO_v1_0591	Solibacter usitatus strain Ellin6076 (Q01YC2, 41.84%)	Methylacidiphilum sp. RTK17.1 (A0A1W5LCU1, 53.05%)	
ctaA	Heme A synthase	1.3.-.-	MTHMO_v1_0592	Bacillus halodurans strain ATCC BAA-125 / DSM 18197 / FERM 7344 / JCM 9153 / C-125 (Q9K9M8, 25.54%)	Methylacidiphilum fumariolicum strain SolV (I0JW24, 51.98%)	
cydA	Cytochrome bd oxidase, subunit I	1.10.3.-	MTHMO_v1_1464	Bacillus subtilis strain 168 (P94364, 38.53%)	Nitrospira sp. SG-bin2 (A0A1W9G997, 62.61%)	
	**Complex V**					
atpC	F1 sector of membrane-bound ATP synthase, epsilon subunit	3.6.3.14	MTHMO_v1_2318	Chloroflexus aggregans strain MD-66 / DSM 9485 (B8G6G5, 37.12%)	Methylacidiphilum sp. RTK17.1 (A0A1W5LD22, 62.79%)	
atpD	F1 sector of membrane-	3.6.3.14	MTHMO_v1_2319	Carboxydothermus	Methylacidiphilum	

	bound ATP synthase, beta subunit			hydrogenoformans strain ATCC BAA-161 / DSM 6008 / Z-2901 (Q3A946, 72.77%)	fumariolicum strain SolV (I0K177, 86.02%)	
atpG	F1 sector of membrane-bound ATP synthase, gamma subunit	3.6.3.14	MTHMO_v1_2320	Methylacidiphilum infernorum (B3E0Z9, 63.01%)	Methylacidiphilum sp. RTK17.1 (A0A1W5LD38, 63.01%)	
atpA	F1 sector of membrane-bound ATP synthase, alpha subunit	3.6.3.14	MTHMO_v1_2321	Carboxydothermus hydrogenoformans strain ATCC BAA-161 / DSM 6008 / Z-2901 (Q3A944, 59.76%)	Methylacidiphilum infernorum (B3E0Z8, 76.47%)	
atpH	F1 sector of membrane-bound ATP synthase, delta subunit	3.6.3.14	MTHMO_v1_2322	Laribacter hongkongensis strain HLHK9 (C1D5G5, 37.86%)	Methylacidiphilum infernorum (B3E0Z7, 48.06%)	
atpF	F0 sector of membrane-bound ATP synthase, subunit b	3.6.3.14	MTHMO_v1_2323	Methylacidiphilum infernorum (B3E0Z6, 60.38%)	Methylacidiphilum kamchatkense Kam1 (A0A0C1RU46, 60%)	
atpE	F0 sector of membrane-bound ATP synthase, subunit c	3.6.3.14	MTHMO_v1_2324	Syntrophus aciditrophicus strain SB (Q2LRB9, 46.88%)	Methylacidiphilum kamchatkense Kam1 (A0A0C1V465, 85.71%)	
atpB	F0 sector of membrane-bound ATP synthase, subunit a	3.6.3.14	MTHMO_v1_2325	Geobacter lovleyi strain ATCC BAA-1151 / DSM 17278 / SZ (B3E9X3, 32.24%)	Methylacidiphilum infernorum (B3E0Z4, 68.61%)	
	**Cytochromes c**					
	Cytochrome c		MTHMO_v1_0540	Drosophila melanogaster (Q8IRW8, 23.6%)	Methylacidiphilum infernorum (B3DUM9, 44%)	
	Cytochrome c		MTHMO_v1_0877	Rhizobium meliloti strain 1021 (Q05577, 42.42%)	Acidobacteriales bacterium 13_1_40CM_3_55_5 (A0A1Q7N4E8, 57.22%)	
	Cytochrome c		MTHMO_v1_0960	Azorhizobium caulinodans strain ATCC 43989 / DSM 5975 / JCM 20966 / NBRC 14845 / NCIMB 13405 / ORS 571 (AP8HZ17, 30.23%)	Methylacidiphilum kamchatkense Kam1 (A0A0C1UMT3, 59.48%)	

	Cytochrome c		MTHMO_v1_1568	Thermus thermophilus strain HB8 / ATCC 27634 / DSM 579 (Q5SME3, 44.53%)	Methylacidiphilum kamchatkense Kam1 (A0A0C1UQ30, 73.88%)	
	Cytochrome c		MTHMO_v1_2049	Thermus thermophilus (P04164, 42.06%)	Methylacidiphilum kamchatkense Kam1 (A0A0C1RJ15, 46.97%)	
cccA	Cytochrome c family protein		MTHMO_v1_2054	Thiomonas intermedia strain K12 (D5WYQ6, 34.695)	Methylacidiphilum infernorum (B3DYG4, 67.66%)	
	Cytochrome c3		MTHMO_v1_2057	Desulfovibrio vulgaris strain Hildenborough / ATCC 29579 / DSM 644 / NCIMB 8303 (P0DOV3, 32.65%)	Methylacidiphilum infernorum (B3DYG8, 68.81%)	

**Nitrogen metabolism**						
**Gene**	**Product**	**EC no.**	**CDS[a]**	**Best BLAST hit in SwissProt[b]**	**Best BLAST hit in TrEMBL[c]**	**Notes**
nifH	Nitrogenase iron protein	1.18.6.1	MTHMO_v1_1014	Acidithiobacillus ferrooxidans strain ATCC 53993 (B5ER76, 88.89%)	Methylacidiphilum fumariolicum (D3XDH4, 90.54%)	
nifD	Nitrogenase alpha chain	1.18.6.1	MTHMO_v1_1015	Bradyrhizobium sp. strain ANU 289 (P06120 , 82.72%)	Methylacidiphilum kamchatkense Kam1 (A0A0C1USX0, 89.16%)	molybdenum-iron protein
nifK	Nitrogenase beta chain	1.18.6.1	MTHMO_v1_1016	Nucleotidyl transferase	Methylacidiphilum sp. RTK17.1 (A0A1W5LDP0, 83.46%)	molybdenum-iron protein
nifE	Nitrogenase iron-molybdenum cofactor, biosynthesis protein NifE		MTHMO_v1_1017	Bradyrhizobium diazoefficiens strain JCM 10833 / IAM 13628 / NBRC 14792 / USDA 110 (P26506, 71.9%)	putative UDP-glucose/GDP-mannose dehydrogenase	
nifN	Nitrogenase iron-molybdenum cofactor, biosynthesis protein NifN		MTHMO_v1_1018	Bradyrhizobium diazoefficiens strain JCM 10833 / IAM 13628 / NBRC 14792 / USDA 110 (P26507, 59.21%)	Sulfuricella sp. T08 (A0A0E9MDH3, 65.62%)	

nifX	Nitrogenase molybdenum-cofactor, protein NifX		MTHMO_v1_1019	Herbaspirillum seropedicae (O87628, 62.68%)	Methylacidiphilum fumariolicum strain SolV (I0JYS0, 72.73%)	
nifA	Nif-specific regulatory protein		MTHMO_v1_1042	Herbaspirillum seropedicae (P27713, 54.03)	Betaproteobacteria bacterium HGW-Betaproteobacteria-1 (A0A2N2VHX2, 55.97%)	
nifB	Nitrogenase FeMo cofactor biosynthesis protein NifB		MTHMO_v1_1045	Herbaspirillum seropedicae (P27714, 76.32%)	Rhizobiales bacterium 32-66-11 (A0A258IKV7, 78.53%)	
nifW	Nitrogenase-stabilizing/protective protein NifW		MTHMO_v1_1055	Methylacidiphilum infernorum (B3DZB3, 49.47%)	Telmatospirillum siberiense (A0A2N3PNB7, 55.47%)	
nifZ	Nitrogen fixation protein NifZ		MTHMO_v1_1049	Sinorhizobium fredii strain NBRC 101917 / NGR234 (Q53203, 50.88%)	Methylacidiphilum infernorum (B3DYZ4, 73.33%)	
nifV	Homocitrate synthase	2.3.3.14	MTHMO_v1_1013	Azotobacter vinelandii (P05342, 54.02%)	Hydrogenophilales bacterium CG18_big_fil_WC_8_21_14_2_50_58_12 (A0A2H0FTE4, 59.24%)	
amtB	fragment of ammonium transporter (part 1)		MTHMO_v1_0690	Escherichia coli strain K12 (P69681, 47.75%)	Methylacidiphilum infernorum (B3DUX7, 70.24%)	
amtB	fragment of ammonium transporter (part 2)		MTHMO_v1_0691	Corynebacterium glutamicum (train ATCC 13032 / DSM 20300 / JCM 1318 / LMG 3730 / NCIMB 10025 (P54146, 28.89%)	Methylacidiphilum infernorum (B3DUX7, 51.06%)	
nasA	Nitrate transporter		MTHMO_v1_1025	Bacillus subtilis strain 168 (P42432, 54.33%)	Kyrpidia tusciae strain DSM 2912 / NBRC 15312 / T2 (D5WXH3, 63.23%)	
	Nitrate ABC transporter, ATP-binding protein		MTHMO_v1_1031	Synechococcus elongatus strain PCC 7942 (Q55107, 37.34%)	Chthoniobacter flavus Ellin428 (B4D7K7, 45.74%)	
	Assimilatory nitrate reductase/Nitrite reductase		MTHMO_v1_1029	Bacillus subtilis strain 168 (P42434, 43.98%)	Cystobacter fuscus (A0A250JEI2, 59.54%)	Putative assimilatory nitrate reductase (nasC)
nirB	Nitrite reductase, large subunit	1.7.1.4	MTHMO_v1_1028	Bacillus subtilis strain 168 (P42435, 42.33%)	Deltaproteobacteria bacterium 13_1_20CM_2_69_21	NAD(P)H

					(A0A1Q7Z3D7, 60.45%)	
nirK	Copper-containing nitrite reductase	1.7.2.1	MTHMO_v1_0885	Neisseria meningitidis serogroup A / serotype 4A strain Z2491 (Q9JTB8, 46.91%)	Methylacidiphilum fumariolicum strain SolV (I0JXK4, 71.38%)	
norB	Nitric oxide reductase subunit B	1.7.2.5	MTHMO_v1_1795	Pseudomonas aeruginosa strain ATCC 15692 / DSM 22644 / CIP 104116 / JCM 14847 / LMG 12228 / 1C / PRS 101 / PAO1 (Q59647, 37.44%)	Thiomonas sp. CB3 (A0A1J5R4U9, 64.03%)	
cynS	Cyanate hydratase	4.2.1.104	MTHMO_v1_1705	Ralstonia pickettii strain 12J (B2UK94, 66.67%)	Thiomonas sp. 15-66-11 (A0A259P4A1, 70.07%)	
glnA	Glutamine synthetase	6.3.1.2	MTHMO_v1_1185	Microchaete diplosiphon (P33035, 62.2%)	Methylacidiphilum kamchatkense Kam1 (A0A0C1UQ57, 83.02%)	
gltS	Glutamate synthase large chain	1.4.1.14, 1.4.7.1, 1.4.1.13	MTHMO_v1_1987	Synechocystis sp. strain PCC 6803 / Kazusa (P55038, 48.8%)	Methylacidiphilum infernorum V4 (B3E0A6, 65.9%)	
gdhA	Glutamate dehydrogenase	1.4.1.3	MTHMO_v1_2125	Thermotoga maritima strain ATCC 43589 / MSB8 / DSM 3109 / JCM 10099 (P96110, 50.61%)	Methylacidiphilum fumariolicum SolV (I0K030, 65.07%)	

**Carbon metabolism**						
	**Carbonate uptake**					
**Gene**	**Product**	**EC no.**	**CDS[a]**	**Best BLAST hit in SwissProt[b]**	**Best BLAST hit in TrEMBL[c]**	**Notes**
can	Carbonic anhydrase	4.2.1.1	MTHMO_v1_0262	Mycobacterium tuberculosis strain CDC 1551 / Oshkosh (P9WPJ6, 39.23%)	Raphidiopsis curvata NIES-932 (A0A1Z4VI12, 72.63%)	
	**Calvin cycle**					
cbbS	Ribulose bisphosphate carboxylase small chain (RuBisCO)	4.1.1.39	MTHMO_v1_1810	Cyanidioschyzon merolae strain 10D (O22024, 59.42%)	Methylacidiphilum fumariolicum (G1BWI0, 69.57%)	

cbbL	Ribulose bisphosphate carboxylase large chain (RuBisCO)	4.1.1.39	MTHMO_v1_1811	Methylacidiphilum infernorum (B3DVG5, 89.26%)	Methylacidimicrobium fagopyrum (G1BWH0, 94.65%)	
pgk	Phosphoglycerate kinase	2.7.2.3	MTHMO_v1_1141	Koribacter versatilis strain Ellin345 (Q1INK7, 55.85%)	Verrucomicrobium sp. GAS474 (A0A1H2GQ13, 63.21%)	
fba	Fructose-bisphosphate aldolase	4.1.2.13	MTHMO_v1_2265	Treponema pallidum strain Nichols (O83668, 43.6%)	Methylacidiphilum sp. RTK17.1 (A0A1W5LD07, 83.7%)	
fbp	Fructose-1,6-bisphosphatase class 1	3.1.3.11	MTHMO_v1_1633	Methylacidiphilum infernorum (B3DWS5, 69.41%)	Methylacidiphilum fumariolicum strain SolV (I0JXL8, 69.41%)	
tktA	Transketolase	2.2.1.1	MTHMO_v1_1807	Geobacillus stearothermophilus (A0A0I9QGZ2, 53.56%)	Methylacidiphilum kamchatkense Kam1 (A0A0C1RIE3, 66.72%)	
prkB	Phosphoribulokinase	2.7.1.19	MTHMO_v1_1632	Cupriavidus necator strain ATCC 17699 / H16 / DSM 428 / Stanier 337 (P19923, 69.2%)	Methylacidiphilum infernorum (A9QPD4, 82.29%)	
tal	Transaldolase	2.2.1.2	MTHMO_v1_2276	Saccharopolyspora erythraea strain ATCC 11635 / DSM 40517 / JCM 4748 / NBRC 13426 / NCIMB 8594 / NRRL 2338 (A4FBP0, 46.07%)	Methylacidiphilum fumariolicum strain SolV (I0JWB7, 65.66%)	
gapA	Glyceraldehyde-3-phosphate dehydrogenase	1.2.1.12	MTHMO_v1_1142	Nostoc sp. strain PCC 7120 / SAG 25.82 / UTEX 2576 (P80506, 59.42%)	Methylacidiphilum sp. RTK17.1 (A0A1W5LCW4, 75.66%)	
tpiA	Triosephosphate isomerase	5.3.1.1	MTHMO_v1_1140	Chloroflexus aurantiacus strain ATCC 29366 / DSM 635 / J-10-fl (P96744, 55.47%)	Methylacidiphilum fumariolicum strain SolV (I0JWK1, 54.37%)	
rpe	D-ribulose-5-phosphate 3-epimerase	5.1.3.1	MTHMO_v1_0671	Synechocystis sp. strain PCC 6803 / Kazusa (P74061, 57.4%)	Methylacidiphilum infernorum (A9QPI5, 76.82%)	
	**Pentose Phosphate pathway**					

zwf	Glucose-6-phosphate 1-dehydrogenase	1.1.1.49	MTHMO_v1_1416	Synechocystis sp. strain PCC 6803 / Kazusa (P73411, 49.59%)	Methylacidiphilum kamchatkense Kam1 (A0A0C1URT3, 64.36%)	
zwf	Glucose-6-phosphate dehydrogenase	1.1.1.49	MTHMO_v1_2095	Synechocystis sp. strain PCC 6803 / Kazusa (P73411, 46.53%)	Methylacidiphilum kamchatkense Kam1 (A0A0C1USR6, 75%)	
pgl	6-phosphogluconolactonase	3.1.1.31	MTHMO_v1_2094	Nostoc sp. strain PCC 7120 / SAG 25.82 / UTEX 2576 (P46016, 37.83%)	Methylacidiphilum fumariolicum strain SolV (I0JY31, 51.14%)	Putative Glucosamine-6-phosphate isomerase/deaminase
gnd	6-phosphogluconate dehydrogenase (decarboxylating)	1.1.1.44	MTHMO_v1_1674	Gluconobacter oxydans strain 621H (G5EBD7, 49.86%)	Lysobacter silvestris (A0A2K1Q219, 69.91%)	NAD(+)-dependent
tktA	Transketolase	2.2.1.1	MTHMO_v1_1807	Geobacillus stearothermophilus (A0A0I9QGZ2, 53.56%)	Methylacidiphilum kamchatkense Kam1 (A0A0C1RIE3, 66.72%)	
tal	Transaldolase	2.2.1.2	MTHMO_v1_2276	Saccharopolyspora erythraea strain ATCC 11635 / DSM 40517 / JCM 4748 / NBRC 13426 / NCIMB 8594 / NRRL 2338 (A4FBP0, 46.07%)	Methylacidiphilum fumariolicum strain SolV (I0JWB7, 65.66%)	
prs	Ribose-phosphate pyrophosphokinase	2.7.6.1	MTHMO_v1_1542	Pseudomonas syringae pv. tomato strain ATCC BAA-871 / DC3000 (Q888C6, 50%)	Methylacidiphilum fumariolicum strain SolV (I0K0F9, 79.05%)	
	**TCA cycle**					
pdhA	Pyruvate dehydrogenase E1 component subunit alpha	1.2.4.1	MTHMO_v1_1745	Rhizobium meliloti strain 1021 (Q9R9N5, 51.13%)	Verrucomicrobium sp. GAS474 (A0A1H2EW72, 62.31%)	
gltA	Citrate synthase	2.3.3.16	MTHMO_v1_1904	Salmonella typhimurium strain LT2 / SGSC1412 / ATCC 700720 (Q56063, 52.72%)	Methylacidiphilum kamchatkense Kam1 (A0A0C1V5N0, 64.95%)	
acnA	Aconitase	4.2.1.3	MTHMO_v1_1134	Thermus thermophilus strain HB8 / ATCC 27634 / DSM 579 (Q5SMF6, 53.75%)	Verrucomicrobia bacterium ADurb.Bin006 (A0A1V6JSJ2, 60.89%)	

icd	Isocitrate dehydrogenase (NADP)	1.1.1.42	MTHMO_v1_0571	Rickettsia bellii strain RML369-C (Q1RJU4, 51.46%)	Methylacidiphilum kamchatkense Kam1 (A0A0C1RJZ6, 69.65%)	
acoB	Pyruvate/2-oxoglutarate dehydrogenase complex, beta subunit	1.2.4.1	MTHMO_v1_1746	Oryza sativa subsp. Japonica (Q0J0H4, 52.01%)	Methylacidiphilum infernorum (B3DUQ7, 75.31%)	
sucC	Succinyl-CoA synthetase subunit beta	6.2.1.5	MTHMO_v1_1152	Akkermansia muciniphila strain ATCC BAA-835 / Muc (B2UMH5, 52.42%)	Verrucomicrobiales bacterium (A0A2D9K1Q8, 51.78%)	ADP forming
sucD	Succinyl-CoA synthetase subunit alpha	6.2.1.5	MTHMO_v1_1153	Aquifex aeolicus strain VF5 (O67547, 60.96%)	Methylacidiphilum kamchatkense Kam1 (A0A0C1RTL7, 68.51%)	NAD(P)-binding
sdhC	Succinate dehydrogenase cytochrome b subunit		MTHMO_v1_0309	no hits	Methylacidiphilum sp. RTK17.1 (A0A1W5LFS3, 49.54%)	
sdhB	Succinate dehydrogenase catalytic subunit	1.3.5.1	MTHMO_v1_0311	Wolinella succinogenes strain ATCC 29543 / DSM 1740 / LMG 7466 / NCTC 11488 / FDC 602W (P17596, 31.78%)	Methylacidiphilum infernorum (A9QPJ6, 65.61%)	
fumC	Fumarate hydratase class II (Fumarase)	4.2.1.2	MTHMO_v1_0708	Thermosynechococcus elongatus strain BP-1 (Q8DIP7, 63.5%)	Methylacidiphilum infernorum (A9QPI3, 62.36%)	
mdh	Malate dehydrogenase	1.1.1.37	MTHMO_v1_0496	Thermosulfidibacter takaii strain DSM 17441 / JCM 13301 / NBRC 103674 / ABI70S6 (A0A0S3QTC6, 55.78%)	Methylacidiphilum kamchatkense Kam1 (A0A0C1USZ0, 67.65%)	
pycA	Pyruvate carboxylase subunit A	6.4.1.1	MTHMO_v1_1880	Bacillus subtilis strain 168 (P49787, 56.33%)	Methylacidiphilum fumariolicum strain SolV (I0JXW1, 69.2%)	
	**Glycolysis / Gluconeogenesis**					
pgm	Phosphoglucomutase	5.4.2.2	MTHMO_v1_1500	Komagataeibacter xylinus (P38569, 60.99%)	Gluconacetobacter diazotrophicus strain ATCC 49037 / DSM 5601 / PAl5 (A9HSH5, 64.52%)	
pgi	Glucose-6-phosphate isomerase	5.3.1.9	MTHMO_v1_1723	Desulfatibacillum alkenivorans strain AK-01	Chthonomonas calidirosea strain DSM 23976 / ICMP 18418 / T49 (S0ES67, 66.05%)	

				(B8FK51, 62.34%)		
pfkA	ATP-dependent 6-phosphofructokinase	2.7.1.11	MTHMO_v1_0286	Chloroflexus aggregans strain MD-66 / DSM 9485 (B8GAA4, 48.9%)	Methylacidiphilum fumariolicum strain SolV (I0K0R8, 68.04%)	
fba	Fructose-bisphosphate aldolase	2.2.1.11, 4.1.2.13	MTHMO_v1_0280	Methanocaldococcus jannaschii strain ATCC 43067 / DSM 2661 / JAL-1 / JCM 10045 / NBRC 100440 (Q58980, 57.77%)	Methylacidiphilum kamchatkense Kam1 (A0A0C1RSD9, 71.86%)	Putative 6-deoxy-5-ketofructose 1-phosphate synthase
tpiA	Triosephosphate isomerase	5.3.1.1	MTHMO_v1_1140	Chloroflexus aurantiacus strain ATCC 29366 / DSM 635 / J-10-fl (P96744, 55.47%)	Methylacidiphilum fumariolicum strain SolV (I0JWK1, 54.37%)	
gapA	Glyceraldehyde-3-phosphate dehydrogenase	1.2.1.12	MTHMO_v1_1142	Nostoc sp. strain PCC 7120 / SAG 25.82 / UTEX 2576 (P80506, 59.42%)	Methylacidiphilum sp. RTK17.1 (A0A1W5LCW4, 75.66%)	
pgk	Phosphoglycerate kinase	2.7.2.3	MTHMO_v1_1141	Koribacter versatilis strain Ellin345 (Q1INK7, 55.85%)	Verrucomicrobium sp. GAS474 (A0A1H2GQ13, 63.21%)	
pyk	Pyruvate kinase	2.7.1.40	MTHMO_v1_2215	Geobacillus stearothermophilus (Q02499, 37.95%)	Methylacidiphilum kamchatkense Kam1 (A0A0C1RUE5, 68.24%)	
gpmA	Phosphoglyceromutase	5.4.2.11	MTHMO_v1_1713	Caldanaerobacter subterraneus subsp. tengcongensis strain DSM 15242 / JCM 11007 / NBRC 100824 / MB4 (Q8R7C8, 64.78%)	Ignavibacterium album strain DSM 19864 / JCM 16511 / NBRC 101810 / Mat9-16 (I0ANS2, 62.75%)	2,3-bisphosphoglycerate-dependent
eno	Enolase	4.2.1.11	MTHMO_v1_0302	Acholeplasma laidlawii strain PG-8A (A9NF93, 59.66%)	Methylacidiphilum kamchatkense Kam1 (A0A0C1URV1, 65.48%)	
ppc	Phosphoenolpyruvate carboxylase	4.1.1.31	MTHMO_v1_2102	Roseiflexus castenholzii strain DSM 13941 / HLO8 (A7NLR3, 40.67%)	Verrucomicrobia bacterium Tous-C9LFEB (A0A2A2R9J4, 54.1%)	
	**Carbon storage**					
glgA	Glycogen synthase	2.4.1.21	MTHMO_v1_2120	Opitutus terrae strain DSM	Verrucomicrobium sp.	

				11246 / JCM 15787 / PB90-1 (B1ZUX5, 43.27%)	GAS474 (A0A1H2GJT4, 52.51%)	
glgA	Glycogen synthase	2.4.1.21	MTHMO_v1_0055	Geobacter sulfurreducens strain ATCC 51573 / DSM 12127 / PCA (Q74ED9, 42.83%)	Methylacidiphilum kamchatkense Kam1 (A0A0C1V2K6, 54.34%)	
glgP	Glycogen phosphorylase/Starch phosphorylase	2.4.1.1	MTHMO_v1_0481	Mycobacterium bovis strain ATCC BAA-935 / AF2122/97 (Q7U078, 40.26%)	Methylacidiphilum sp. RTK17.1 (A0A1W5LD14, 59.07%)	
	**Acetate metabolism**					
ackA	Acetate kinase	2.7.2.1	MTHMO_v1_0263	Rhodopseudomonas palustris strain ATCC BAA-98 / CGA009 (Q6N143, 44.75%)	candidate division TA06 bacterium SM23_40 (A0A0S8GDY7, 52.82%)	

**Hydrogen metabolism**						
Gene	**Product**	**EC no.**	**CDS[a]**	**Best BLAST hit in SwissProt[b]**	**Best BLAST hit in TrEMBL[c]**	**Notes**
	NADH-quinone oxidoreductase subunit NuoB / Hydrogenase		MTHMO_v1_0659	Escherichia coli strain K12 (P16431, 29.71%)	Betaproteobacteria bacterium RIFCSPLOWO2_12_FULL_67_28 (A0A1F4F0Q6, 75%)	
hypE	Hydrogenase expression protein HypE		MTHMO_v1_0660	Escherichia coli strain K12 (P16433, 42.14%)	Mesorhizobium sp. LNHC221B00 (X6DLR7, 63.56%)	
hyfF	Hydrogenase-4 subunit F		MTHMO_v1_0661	Methylacidiphilum infernorum (A9QPJ1, 61.18%)	Phyllobacterium brassicacearum (A0A2P7B5H6, 65.56%)	
hyfE	Hydrogenase-4 subunit E		MTHMO_v1_0662	Mycobacterium bovis strain ATCC BAA-935 / AF2122/97 (P64682, 27.31%)	Inquilinus limosus (A0A211ZQP8, 74.55%)	
hypE	Hydrogenase expression/formation protein HypE		MTHMO_v1_1364	Azotobacter vinelandii (P40595, 51.2%)	Spirosoma montaniterrae (A0A1P9WU63, 59.06%)	
hypD	Hydrogenase		MTHMO_v1_1365	Rhodobacter capsulatus	Runella slithyformis strain ATCC 29530 / DSM	

	expression/formation protein HypD			(P26411, 47.48%)	19594 / LMG 11500 / NCIMB 11436 / LSU 4 (F8EQR2, 64.27%)	
hypC	Hydrogenase assembly chaperone HypC		MTHMO_v1_1366	Synechocystis sp. strain PCC 6803 / Kazusa (P74095, 46.67%)	Spirosoma linguale strain ATCC 33905 / DSM 74 / LMG 10896 (D2QHS0, 58.82%)	
hypF	Carbamoyltransferase	6.2.-.-	MTHMO_v1_1367	Synechocystis sp. strain PCC 6803 / Kazusa (Q55638, 39.35%)	Methylacidiphilum infernorum (B3DXE2, 53.07%)	
hypB	Hydrogenase maturation factor HypB		MTHMO_v1_1369	Azotobacter chroococcum mcd 1 (Q43949, 49.29%)	Methylacidiphilum sp. RTK17.1 (A0A1W5LCR8, 62.04%)	
hypA	Hydrogenase maturation factor HypA		MTHMO_v1_1370	Acidithiobacillus ferrooxidans strain ATCC 53993 (B5ERM3, 46.9%)	Acidiferrobacter thiooxydans (A0A1C2G411, 48.21%)	
hynA	Ni-Fe hydrogenase, small subunit	1.12.99.6	MTHMO_v1_1378	Wolinella succinogenes strain ATCC 29543 / DSM 1740 / LMG 7466 / NCTC 11488 / FDC 602W (P31884, 50.72%)	Sulfurovum riftiae (A0A151CIW0, 69.67%)	
hynB	Ni-Fe hydrogenase, large subunit	1.12.99.6	MTHMO_v1_1379	Wolinella succinogenes strain ATCC 29543 / DSM 1740 / LMG 7466 / NCTC 11488 / FDC 602W (P31883, 47.39%)	Hydrogenimonas thermophila (A0A1I5P8H0, 65.96%)	O2 sensitive
hynC	Ni-Fe hydrogenase, cytochrome b subunit		MTHMO_v1_1380	Rhodobacter capsulatus (P16145, 26.34%)	Persephonella hydrogeniphila (A0A285NC48, 61.16%)	
hupD	Hydrogenase expression/formation protein HupD		MTHMO_v1_1381	Rhodobacter capsulatus (Q03004, 33.33%)	Sulfurihydrogenibium azorense strain Az-Fu1 / DSM 15241 / OCM 825 (C1DUF5, 38.79%)	
hupQ	Putative Hydrogenase expression/formation protein HupQ		MTHMO_v1_1497	Azotobacter chroococcum mcd 1 (Q43957, 28.57%)	Alteromonas confluentis (A0A1E7ZDF7, 31.3%)	

**Sulfur metabolism**						
	**Assimilatory sulfate reduction**					
**Gene**	**Product**	**EC no.**	**CDS**[a]	**Best BLAST hit in SwissProt**[b]	**Best BLAST hit in TrEMBL**[c]	**Notes**

sul	Sulfate permease		MTHMO_v1_0976	Arabidopsis thaliana (Q9FY46, 25.88%)	Methylacidiphilum kamchatkense Kam1 (A0A0C1RW94, 59.42%)	MFS superfamily
cysH	3'-phosphoadenosine 5'-phosphosulfate reductase	1.8.4.8	MTHMO_v1_1920	Alkalilimnicola ehrlichii strain ATCC BAA-1101 / DSM 17681 / MLHE-1 (Q0A6T1, 59.07%)	Gammaproteobacteria bacterium HGW-Gammaproteobacteria-5 (A0A2N1WZP9, 58.05%)	
cysD	Sulfate adenylyltransferase subunit 2	2.7.7.4	MTHMO_v1_1921	Rhodopseudomonas palustris strain BisB18 (Q21D94, 44.07%)	Verrucomicrobia bacterium 13_2_20CM_2_54_15_9cls (A0A1Q6X161, 77.44%)	
cysC	Adenylyl-sulfate kinase	2.7.1.25	MTHMO_v1_1922	Rhizobium meliloti strain 1021 (P13442, 40.03%)	Verrucomicrobium sp. GAS474 (A0A1H2FPR4, 55.25%)	
cysI	Sulfite reductase beta subunit	1.8.1.2	MTHMO_v1_0862	Staphylococcus saprophyticusstrain ATCC 15305 / DSM 20229 (Q49UL9, 43.12%)	Methylacidiphilum infernorum (B3DWS0, 61.35%)	
cysJ	Sulfite reductase, alpha subunit (Flavoprotein)	1.8.1.2	MTHMO_v1_0812	Bacillus subtilis strain 168 (O32214, 45.79%)	Methylacidiphilum infernorum (B3DXG4, 59.74%)	
	**Sulfite oxidation**					
suox	Sulfite oxidase	1.8.3.1	MTHMO_v1_1534	Mus musculus (Q8R086, 33.01%)	Methylacidiphilum infernorum (B3E067, 55.88%)	
sseA	Sulfurtransferase		MTHMO_v1_1022	Thermus thermophilus strain HB27 / ATCC BAA-163 / DSM 7039 (Q72JV2, 61.4%)	Verrucomicrobia bacterium 13_2_20CM_2_54_15_9cls (A0A1Q6X056, 76.34%)	

**Transporters / Metal related proteins**						
**Gene**	**Product**	**EC no.**	**CDS**[a]	**Best BLAST hit in SwissProt**[b]	**Best BLAST hit in TrEMBL**[c]	**Notes**
	Metal transporter		MTHMO_v1_0904	no hits	Methylacidiphilum kamchatkense Kam1 (A0A0C1UU90, 55.4%)	

pstB	Phosphate import ATP-binding protein PstB	3.6.3.27	MTHMO_v1_0313	Frankia casuarinae strain DSM 45818 / CECT 9043 / CcI3 (Q2J534, 60.87%)	Methylacidiphilum infernorum (B3DYS7, 73.18%)	
pstA	Phosphate transport system permease protein PstA		MTHMO_v1_0314	Yersinia pestis (P58655, 40.44%)	Verrucomicrobia bacterium RIFCSPLOWO2_12_FULL_64_8 (A0A1G3ZMH2, 62.69%)	
pstC	Phosphate transport system permease protein PstC		MTHMO_v1_0315	Mesorhizobium japonicum strain LMG 29417 / CECT 9101 / MAFF 303099 (Q98FL3, 41.28%)	Verrucomicrobia bacterium 13_1_20CM_54_28 (A0A1Q7S5V9, 57.89%)	
pstS	Phosphate-binding protein PstS		MTHMO_v1_0316	Xylella fastidiosa strain Temecula1 / ATCC 700964 (Q87C91, 44.48%)	Verrucomicrobia bacterium 13_1_20CM_3_54_17 (A0A1Q7V1Z0, 57.7%)	

[a] *Methylacidimicrobium thermophilum* AP8 protein identifier
[b] Organism with highest scoring BLAST hit to *M. thermophilum* AP8 protein in SwissProt database. In parentheses: SwissProt accession number of best hit, amino acid identity.
[c] Organism with highest scoring BLAST hit to *M. thermophilum* AP8 protein in TrEMBL database. In parentheses: TrEMBL accession number of best hit, amino acid identity.

Table S4. Transcriptomics analysis in *M. thermophilum* AP8.

Gene	Sample 1 (RPKM)	Sample 2 (RPKM)	Sample 3 (RPKM)	Average RPKM	Standard deviation
pmoC1	71231	72562	71107	71633	807
pmoB2	32741	30546	30259	31182	1358
MTHMO_v1_0031	28400	27412	26777	27530	818
pmoA2	20458	19383	19213	19685	675
MTHMO_v1_0786	15940	14294	17270	15834	1491
xoxF1	15017	13112	13773	13967	967
rbpF	8939	10632	10596	10056	967
MTHMO_v1_1299	6347	7194	7484	7009	591
MTHMO_v1_1572	6013	6220	6606	6279	301
MTHMO_v1_0559	5580	6769	6419	6256	611
groEL	5508	4934	5202	5215	287
MTHMO_v1_0888	4768	5153	4883	4935	197
MTHMO_v1_0197	4119	4786	4506	4470	335
cbbL	4642	4470	4174	4429	237
MTHMO_v1_1353	4003	4580	4058	4214	318
tufB	3595	3634	3914	3714	174
MTHMO_v1_2205	3146	4426	3392	3655	680
MTHMO_v1_1812	4174	2835	3839	3616	697
MTHMO_v1_1889	3136	4022	3434	3531	451
rpmG	3663	3347	3561	3524	161
glnK	2985	3043	3822	3284	467
groS	3243	2780	3747	3257	483
hupA	2870	3101	3524	3165	332
infC	3209	2732	3259	3067	291
rplT	2973	2618	3080	2890	242
dksA	3084	2699	2805	2863	199
MTHMO_v1_1780	2337	3160	2577	2691	423
MTHMO_v1_0456	2393	2568	2426	2462	93
fba_2	2181	2450	2574	2402	201
rpsB	1906	2545	2573	2341	377
acpP	2046	2191	2447	2228	203
MTHMO_v1_0116	1877	2264	2398	2180	270
clpP	2072	2117	2315	2168	129
sseA	2066	2099	2257	2140	102
MTHMO_v1_0175	1861	2489	2064	2138	321
coaE	2168	2037	2095	2100	66
MTHMO_v1_0174	1661	2675	1913	2083	528

clpS	1761	2242	2166	2056	258
rpsO	1879	2120	1927	1975	127
MTHMO_v1_0356	2051	2115	1728	1965	208
MTHMO_v1_1754	1963	2113	1724	1933	196
rpsR	1969	1649	1949	1856	179
MTHMO_v1_2251	1749	1963	1769	1827	118
MTHMO_v1_0131	1635	1938	1838	1804	154
fusA	1610	1927	1862	1800	168
MTHMO_v1_1422	1519	1848	1992	1786	242
rplM	1845	1705	1762	1771	71
MTHMO_v1_0952	1779	1913	1578	1756	169
MTHMO_v1_1103	1976	1556	1730	1754	211
ymdB_2	1929	1588	1630	1716	186
xoxG	1764	1692	1566	1674	100
efp	1630	1818	1452	1634	183
MTHMO_v1_1109	1351	1964	1567	1628	311
MTHMO_v1_2200	1506	1655	1655	1605	86
MTHMO_v1_0906	1393	1894	1402	1563	286
rpsL	1497	1477	1679	1551	112
prkB	1374	1670	1586	1543	152
cbbS	1385	1754	1470	1537	193
nuoB	1325	1961	1312	1532	371
MTHMO_v1_0254	1570	1590	1415	1525	96
bcp_2	1542	1471	1469	1494	42
can	1345	1468	1569	1461	113
MTHMO_v1_1560	1359	1606	1403	1456	132
gapA	1403	1578	1325	1435	130
MTHMO_v1_0473	1422	1541	1334	1432	104
MTHMO_v1_2203	1575	1535	1180	1430	217
rpsA	1400	1356	1519	1425	84
MTHMO_v1_1800	1492	1491	1271	1418	128
clpB_2	1374	1443	1417	1411	35
rpsG	1352	1439	1439	1410	50
rpsK	1636	1074	1510	1407	295
MTHMO_v1_1766	1268	1481	1359	1369	107
rpsM	1554	998	1494	1349	305
MTHMO_v1_1120	1164	1643	1205	1337	266
pnp	1272	1386	1238	1299	77
MTHMO_v1_1395	1007	1595	1291	1298	294
rpoB	1283	1283	1306	1290	13
tsf	1331	1222	1312	1288	58
MTHMO_v1_0177	1410	1357	1086	1284	173
atpC	1389	1290	1165	1281	112

MTHMO_v1_0171	1099	1702	1010	1270	377
MTHMO_v1_1617	1497	1247	1049	1264	225
tas	1115	1329	1324	1256	123
glnA	1287	1209	1251	1249	39
nuoI	1102	1377	1257	1245	138
MTHMO_v1_0110	1072	1254	1357	1228	144
MTHMO_v1_0631	1151	1179	1345	1225	105
dnaK	1190	1199	1274	1221	46
cyoA	1148	1308	1170	1209	87
rpsQ	1410	933	1246	1196	242
MTHMO_v1_0189	943	1536	1100	1193	307
nusG	1301	1045	1221	1189	131
MTHMO_v1_0890	756	1207	1599	1187	422
cyoB_2	1097	1242	1196	1178	74
MTHMO_v1_1520	1174	1057	1299	1177	121
MTHMO_v1_1568	1254	1267	987	1169	158
nusA	1088	1098	1293	1160	115
ssb	1009	1186	1246	1147	123
MTHMO_v1_0973	930	1169	1296	1132	186
sodA	1066	1215	1109	1130	77
cueO	1083	1153	1141	1125	37
MTHMO_v1_1658	1202	887	1274	1121	206
MTHMO_v1_1485	1125	976	1182	1094	107
MTHMO_v1_1420	982	1240	1030	1084	137
MTHMO_v1_2047	849	1098	1296	1081	224
cyoB_1	991	1192	1058	1081	102
frr	1103	1287	810	1067	241
MTHMO_v1_1479	1155	777	1267	1066	257
gmk	1104	970	1032	1035	67
mraZ	1107	1179	813	1033	194
rpoC	1100	977	1013	1030	63
cysK	1212	925	952	1030	158
sufB_1	1018	1079	989	1029	46
secE	1137	1093	841	1024	160
trxA	900	1176	964	1013	144
MTHMO_v1_1173	1208	859	972	1013	178
dxs	1055	1079	902	1012	96
hisI	1050	995	982	1009	36
rplS	1026	924	1044	998	65
rplW	1045	1001	916	987	66
MTHMO_v1_1288	877	1096	955	976	111
MTHMO_v1_2060	942	934	1048	975	64

nuoC	944	926	1050	973	67
MTHMO_v1_0493	1100	902	915	972	111
MTHMO_v1_1893	876	1158	868	967	165
ccpA_1	959	978	948	962	15
rpe	801	1070	998	956	139
MTHMO_v1_1060	985	998	881	955	64
fdsA	914	1064	882	953	97
rplE	866	977	1011	952	76
MTHMO_v1_0805	840	1279	724	948	293
uspA	940	836	1020	932	92
MTHMO_v1_0198	919	866	1006	930	71
MTHMO_v1_0899	966	847	972	929	70
bcp_1	911	977	890	926	46
MTHMO_v1_1799	986	1027	763	925	142
MTHMO_v1_0867	871	1000	900	924	68
erfK	1001	874	895	924	68
MTHMO_v1_0907	775	1102	888	922	166
sqhC	920	892	916	909	16
rplO	994	970	753	906	133
ilvH	858	934	900	897	38
MTHMO_v1_0865	999	900	776	892	112
MTHMO_v1_2328	1021	915	710	882	158
exbD_1	953	851	828	877	67
MTHMO_v1_1665	858	806	952	872	74
pgk	738	879	985	867	124
rpmE	823	932	842	866	58
fdsG	864	934	791	863	72
citB	808	870	908	862	51
MTHMO_v1_0864	889	679	1018	862	171
MTHMO_v1_2266	907	1271	406	861	434
MTHMO_v1_2364	776	928	866	856	76
fbp	948	819	791	853	84
MTHMO_v1_2365	740	1044	771	852	168
tktA	817	838	900	852	43
mreB	786	916	851	851	65
MTHMO_v1_1963	720	1056	771	849	181
atpD_2	890	839	778	836	56
MTHMO_v1_0357	859	848	797	835	34
metK	562	1307	627	832	413
hisG	776	850	842	823	41
eno	719	890	847	818	89
map	900	798	756	818	74
grpE	732	702	1013	816	172

MTHMO_v1_2033	978	689	747	805	153
prc	730	890	788	803	81
hynA	693	942	770	802	127
acrA_2	732	854	816	800	62
fdsD	521	894	975	796	242
MTHMO_v1_0871	611	810	957	793	173
MTHMO_v1_2130	688	887	802	792	100
MTHMO_v1_2045	681	890	804	792	105
MTHMO_v1_1349	764	865	734	788	69
MTHMO_v1_1352	688	909	761	786	113
MTHMO_v1_2175	826	703	821	783	69
paaD	755	869	715	780	80
MTHMO_v1_1431	564	758	1007	776	222
ispH	792	747	784	774	24
MTHMO_v1_0353	742	794	768	768	26
MTHMO_v1_1787	701	866	723	764	90
MTHMO_v1_1832	955	632	696	761	171
rplR	756	724	800	760	38
MTHMO_v1_0119	780	746	742	756	21
MTHMO_v1_0946	870	781	601	751	137
MTHMO_v1_1539	666	786	794	748	72
purS	641	736	863	747	111
clpB_1	708	796	735	746	45
MTHMO_v1_1735	557	890	788	745	170
infB	748	694	790	744	48
hynB	625	814	790	743	103
trmD	886	577	758	740	156
atpA_2	733	726	750	736	13
MTHMO_v1_0321	839	936	433	736	267
rpsH	778	635	781	731	84
rplN	694	797	685	726	62
soj	798	656	717	724	71
MTHMO_v1_0688	523	920	722	722	198
rplC	835	671	639	715	105
MTHMO_v1_0179	662	819	664	715	90
his	721	709	701	710	10
rpsJ	779	622	729	710	80
sufC	624	836	657	706	114
ompW	785	764	561	703	123
guaB	669	770	660	700	61
serA	729	680	681	697	28
MTHMO_v1_0636	650	707	733	696	42

rplB	654	646	788	696	80
ywfI	689	698	694	693	5
leuD	653	747	675	692	49
MTHMO_v1_1351	799	750	526	692	146
MTHMO_v1_1167	712	732	630	691	54
MTHMO_v1_1781	682	799	591	691	104
gyrA	612	720	739	690	69
argB	756	595	720	690	84
pdhA	568	840	662	690	138
rpsD	695	699	669	688	17
MTHMO_v1_1884	627	758	677	687	66
bfrB	601	880	578	686	168
mcsB	592	926	540	686	209
typA	653	708	695	685	29
rplQ	780	593	674	682	94
MTHMO_v1_1174	660	697	688	681	20
MTHMO_v1_0689	623	842	576	681	142
MTHMO_v1_0740	405	818	812	678	237
rpmI	669	627	735	677	54
MTHMO_v1_0293	604	727	698	676	64
MTHMO_v1_1396	549	671	808	676	129
yajC	642	674	709	675	33
rplK	801	602	619	674	110
ftsH_2	645	702	667	672	29
ndk	600	721	682	668	62
xerC	694	664	642	667	26
MTHMO_v1_0873	636	714	636	662	45
MTHMO_v1_0040	616	745	621	661	73
rplD	750	656	567	658	92
rpoA	624	616	725	655	61
MTHMO_v1_2059	771	580	613	655	102
pdhC	651	625	666	648	21
MTHMO_v1_0099	606	718	618	647	62
MTHMO_v1_0004	509	758	647	638	125
MTHMO_v1_0492	675	515	705	632	102
MTHMO_v1_1809	706	561	623	630	73
atpF_2	661	561	660	627	58
ychF	582	764	530	625	123
MTHMO_v1_1663	646	614	609	623	20
MTHMO_v1_1736	540	699	628	623	79
MTHMO_v1_0669	543	621	701	622	79
ybeZ	472	688	704	621	130
MTHMO_v1_1706	646	705	497	616	107

nuoD	629	641	563	611	42
MTHMO_v1_1184	657	441	731	610	151
hcaD	632	570	625	609	34
zraR_2	657	639	527	608	70
MTHMO_v1_0219	513	665	643	607	82
phoE	436	734	628	599	151
dxr	563	593	641	599	39
MTHMO_v1_1974	456	710	625	597	129
MTHMO_v1_2044	582	705	502	597	102
MTHMO_v1_0362	499	656	635	596	85
folE	602	580	603	595	13
MTHMO_v1_1430	698	563	520	594	93
MTHMO_v1_1677	583	621	561	588	30
tig	565	590	608	588	21
ccmA	599	599	563	587	21
MTHMO_v1_2211	448	693	616	585	125
gcd	480	741	525	582	140
metE	581	620	542	581	39
mqnC	586	550	598	578	25
MTHMO_v1_2139	547	634	553	578	49
ilvC	538	617	573	576	39
kdsD	688	560	476	575	107
MTHMO_v1_1928	615	608	495	573	67
rplX	614	708	393	572	161
rpsI	439	539	731	570	148
MTHMO_v1_0991	639	563	498	567	71
MTHMO_v1_1286	500	676	521	566	96
rplF	554	570	569	564	9
MTHMO_v1_0190	545	598	540	561	32
MTHMO_v1_1453	522	631	525	559	62
rfaE	598	482	594	558	66
MTHMO_v1_2171	525	593	555	558	34
fdh	523	581	570	558	31
rplP	471	551	649	557	89
MTHMO_v1_0531	537	583	536	552	26
MTHMO_v1_0876	557	501	591	550	46
MTHMO_v1_0486	417	710	521	549	148
ptsH	495	472	675	548	111
pyrH	584	579	467	543	66
MTHMO_v1_1606	489	666	470	542	108
MTHMO_v1_0213	572	538	515	542	29
nrdB	510	587	527	541	40

MTHMO_v1_1454	583	621	401	535	118
tal	493	629	480	534	83
thiC	516	517	559	531	25
MTHMO_v1_2071	603	496	492	530	63
MTHMO_v1_1264	409	495	686	530	142
MTHMO_v1_0204	483	561	542	529	41
fabF	619	510	451	527	86
MTHMO_v1_1123	531	443	600	525	79
MTHMO_v1_1845	471	612	484	523	78
dop	530	466	568	521	52
yrzS	502	485	574	520	47
secA	483	545	529	519	32
aspC	534	453	569	519	60
rplI	589	553	411	518	94
nrdA	484	503	562	517	41
aat	452	583	512	515	65
ruvC	495	623	410	509	107
MTHMO_v1_1605	409	465	643	506	122
MTHMO_v1_1891	640	555	319	505	166
MTHMO_v1_1517	504	523	485	504	19
MTHMO_v1_1442	445	581	479	501	71
pyk	480	502	517	500	18
MTHMO_v1_0468	543	624	330	499	152
MTHMO_v1_0423	448	526	520	498	44
MTHMO_v1_1419	437	404	641	494	129
rpmA	420	530	530	493	64
MTHMO_v1_2103	426	467	578	490	79
dnaA	436	472	551	486	59
MTHMO_v1_0904	494	589	374	486	108
MTHMO_v1_1803	432	487	532	484	50
rpsP	527	434	484	482	47
purA	429	571	443	481	78
MTHMO_v1_0382	449	574	416	480	83
sufB_2	449	538	452	480	51
MTHMO_v1_2019	330	685	423	479	184
MTHMO_v1_1755	503	431	501	478	42
MTHMO_v1_1888	353	622	458	478	135
MTHMO_v1_0624	473	442	512	476	35
moeZ	615	407	401	474	122
MTHMO_v1_0863	436	600	385	473	112
ccpA_2	442	593	384	473	108
hisJ	449	443	513	469	39
lysR_1	437	568	393	466	91

purE	464	508	421	465	44
MTHMO_v1_0731	324	610	455	463	143
MTHMO_v1_0212	381	485	523	463	74
MTHMO_v1_1321	410	474	505	463	48
MTHMO_v1_0337	531	463	388	461	71
MTHMO_v1_0846	451	529	400	460	65
acyP	367	469	542	460	88
sppA	495	400	482	459	51
MTHMO_v1_2202	408	516	451	458	54
himA	418	477	479	458	34
rplY	590	233	548	457	195
pre	429	447	495	457	34
cda	346	529	490	455	97
MTHMO_v1_1289	419	472	474	455	31
rpoD	480	372	509	454	73
rpsZ	324	517	519	453	112
rho	466	479	413	453	35
exbD_3	290	470	584	448	148
degQ	457	511	374	447	69
cysI	461	422	458	447	21
hisS	380	502	456	446	61
MTHMO_v1_1878	374	556	405	445	97
MTHMO_v1_0225	450	479	401	444	39
MTHMO_v1_2208	416	472	438	442	28
infA	488	433	402	441	43
MTHMO_v1_1095	275	565	479	440	149
MTHMO_v1_1561	438	459	421	440	19
clpX_1	427	522	370	440	77
MTHMO_v1_1179	491	463	363	439	67
xenB_2	488	373	447	436	58
MTHMO_v1_0709	402	413	493	436	50
cmoB	409	405	491	435	49
MTHMO_v1_2343	374	501	430	435	64
fdsB	424	458	420	434	21
MTHMO_v1_0824	455	424	416	431	21
MTHMO_v1_1830	307	542	443	431	118
mcrA	368	516	407	430	77
MTHMO_v1_0761	298	517	474	430	116
MTHMO_v1_0205	393	392	495	427	59
MTHMO_v1_0005	497	319	462	426	94
tpiA	376	465	437	426	46
MTHMO_v1_0528	440	435	403	426	20

MTHMO_v1_2293	435	416	424	425	10
MTHMO_v1_0698	391	478	404	424	47
htrA	377	388	503	423	70
MTHMO_v1_1130	367	522	377	422	86
MTHMO_v1_1619	397	502	366	422	72
gyrB	393	474	398	421	46
MTHMO_v1_1404	407	430	427	421	12
MTHMO_v1_1749	425	484	355	421	65
MTHMO_v1_0334	440	419	405	421	17
MTHMO_v1_0677	311	469	482	421	95
MTHMO_v1_2194	374	554	329	419	119
nuoF	408	455	393	419	32
hppA	430	383	442	419	31
ispA	423	440	388	417	27
rpmB	401	440	410	417	21
zwf_2	331	535	384	417	106
rpmF	432	498	311	413	94
gloB_1	428	407	404	413	13
pepA	399	442	393	411	27
rpsS	308	555	365	410	129
MTHMO_v1_1439	557	297	369	407	135
rpsU	377	402	429	403	26
ptsN	291	481	434	402	99
adh	446	447	312	402	78
yugG	360	433	411	402	37
MTHMO_v1_1548	360	420	418	399	34
glmS	438	414	345	399	48
gltX	382	428	381	397	27
MTHMO_v1_0901	372	374	437	394	37
argD_1	360	395	423	393	32
cirA_6	363	375	438	392	40
argJ	326	358	488	391	86
MTHMO_v1_0639	381	425	366	391	31
atpD_1	390	440	341	390	49
sucB	391	419	360	390	30
slp_2	391	448	331	390	59
MTHMO_v1_1317	344	504	322	390	99
dapF	408	374	385	389	18
MTHMO_v1_0687	514	445	206	389	162
exbD_2	431	335	399	388	48
glyA	406	403	355	388	28
MTHMO_v1_2334	499	357	305	387	101
prs	392	417	351	387	34

MTHMO_v1_0054	312	466	380	386	77
ispG	365	408	384	385	22
secY	398	392	365	385	17
atpG_2	471	378	304	384	83
MTHMO_v1_0469	425	379	342	382	41
secD	337	410	397	381	39
MTHMO_v1_0170	193	659	291	381	246
MTHMO_v1_1589	301	360	482	381	92
mcsA	410	415	316	380	56
MTHMO_v1_0238	376	394	370	380	13
MTHMO_v1_0638	290	429	418	379	77
MTHMO_v1_1508	337	369	423	377	44
lpxD	372	382	376	376	5
MTHMO_v1_0929	365	334	429	376	48
arc	384	363	381	376	11
ribBA	383	353	389	375	19
hisB	430	376	318	375	56
aspS	356	397	368	373	21
thiG	370	346	404	373	29
MTHMO_v1_1701	393	348	378	373	23
MTHMO_v1_1448	458	299	361	373	80
pps	341	392	383	372	27
plsX	357	420	337	371	44
MTHMO_v1_2009	335	412	362	370	39
MTHMO_v1_1862	346	414	349	370	38
ilvB	348	399	359	369	27
tatA	338	443	325	369	65
MTHMO_v1_0206	388	329	387	368	34
MTHMO_v1_2046	375	493	234	367	130
acnA	364	375	363	367	6
MTHMO_v1_0813	377	390	331	366	31
fabG_3	400	329	368	366	36
MTHMO_v1_0182	255	475	368	366	110
MTHMO_v1_1023	368	330	398	365	34
MTHMO_v1_0835	383	403	309	365	50
MTHMO_v1_0554	332	373	390	365	30
cynS	392	393	304	363	51
MTHMO_v1_0083	331	414	337	361	47
leuA	340	349	391	360	27
spo0C	345	367	368	360	13
ywlF	286	313	478	359	104
MTHMO_v1_1083	291	357	426	358	68

MTHMO_v1_1355	330	378	365	358	25
MTHMO_v1_2329	378	402	294	358	57
glyQS	340	339	394	358	31
folC	299	432	342	358	68
MTHMO_v1_0475	372	350	350	357	12
MTHMO_v1_0684	317	390	358	355	37
MTHMO_v1_0301	272	347	436	352	82
MTHMO_v1_1758	256	409	390	352	83
MTHMO_v1_1773	286	346	419	350	67
cafA	349	367	331	349	18
queE	383	329	331	348	31
ahcY	328	381	329	346	30
smpB	388	367	277	344	59
nifS_1	315	348	369	344	27
MTHMO_v1_0764	439	198	390	342	128
MTHMO_v1_0259	343	379	304	342	37
MTHMO_v1_1124	242	368	416	342	90
phoU	280	330	413	341	67
proS	361	288	373	341	46
thrA	372	347	304	341	34
MTHMO_v1_2075	348	345	326	340	12
yrbF	383	275	360	340	57
purF	315	365	338	339	25
MTHMO_v1_2259	288	386	339	338	49
pol	322	382	306	337	40
mqnE	356	349	305	337	28
hynC	330	342	335	336	6
leuC	365	324	318	336	26
MTHMO_v1_1429	279	351	375	335	50
mreC	322	295	389	335	48
hflX	333	316	353	334	18
MTHMO_v1_2058	326	381	293	333	44
gpmA	349	340	311	333	20
MTHMO_v1_1125	487	284	226	332	137
rplL	372	315	309	332	35
MTHMO_v1_1490	507	243	244	331	152
tuaD	285	306	399	330	61
rpoE_1	287	305	393	328	57
MTHMO_v1_1691	413	369	203	328	111
MTHMO_v1_1071	318	380	286	328	48
gltA	325	321	338	328	8
ysh	314	304	361	326	31
MTHMO_v1_1403	294	364	319	326	36

rplA	410	345	220	325	96
htpX	316	360	297	325	32
MTHMO_v1_0136	211	341	421	324	106
MTHMO_v1_1344	345	270	351	322	45
MTHMO_v1_0338	275	298	391	321	61
MTHMO_v1_1402	321	281	361	321	40
MTHMO_v1_1744	257	310	396	321	70
MTHMO_v1_2304	317	312	332	321	11
gatB	313	316	332	320	10
MTHMO_v1_0804	302	344	311	319	22
MTHMO_v1_1833	334	288	330	317	25
pmoC2	317	194	435	316	120
MTHMO_v1_1297	248	395	303	315	75
MTHMO_v1_1401	368	258	317	314	55
MTHMO_v1_0059	248	345	348	314	57
ilvE	295	272	373	313	53
MTHMO_v1_2057	266	386	285	313	65
MTHMO_v1_1168	205	409	320	312	102
MTHMO_v1_1825	328	331	275	312	32
MTHMO_v1_0705	304	348	278	310	35
MTHMO_v1_0186	196	313	418	309	111
pgi	341	331	254	309	48
MTHMO_v1_0634	282	300	336	306	27
MTHMO_v1_0790	355	259	302	305	48
nuoN	293	332	286	304	25
ygcM	288	364	259	304	54
MTHMO_v1_1688	382	228	298	303	77
MTHMO_v1_1657	174	231	501	302	175
MTHMO_v1_1493	180	346	380	302	107
MTHMO_v1_0366	312	260	334	302	38
MTHMO_v1_0593	323	337	245	302	50
MTHMO_v1_0753	287	311	306	301	13
MTHMO_v1_1415	214	352	337	301	76
nuoA	336	311	255	301	41
mdmC	271	283	348	301	41
MTHMO_v1_0843	233	321	345	300	59
cotSA	259	305	332	298	37
MTHMO_v1_2234	340	305	250	298	46
MTHMO_v1_1098	356	229	306	297	64
MTHMO_v1_0858	228	338	325	297	60
MTHMO_v1_1586	258	333	294	295	37
pgl	354	220	312	295	69

MTHMO_v1_0445	317	301	267	295	26
pmoB1	219	330	334	294	65
MTHMO_v1_0047	225	369	288	294	72
MTHMO_v1_0875	299	230	352	294	61
MTHMO_v1_0791	304	282	292	293	11
MTHMO_v1_0670	254	288	336	293	42
MTHMO_v1_1489	185	381	311	292	99
hemC	272	300	302	291	17
pncA_2	263	273	337	291	40
MTHMO_v1_0416	354	277	240	291	58
MTHMO_v1_0533	316	332	221	290	60
MTHMO_v1_0295	336	256	274	289	42
ubiE_1	254	292	315	287	31
MTHMO_v1_2087	272	340	248	287	48
dacC_2	291	363	206	287	78
yugH	287	326	246	286	40
MTHMO_v1_2292	323	287	248	286	37
pyrG	271	291	297	286	13
hypC	135	419	303	286	143
nuoJ	326	282	248	286	39
cysJ	240	295	319	285	40
MTHMO_v1_0752	375	189	288	284	93
MTHMO_v1_0849	261	221	370	284	77
argE_1	259	333	260	284	42
trxB	234	264	354	284	63
fhs	291	314	245	284	35
ftsI	270	296	284	283	13
pfkA	250	322	270	281	37
MTHMO_v1_2063	243	280	318	280	38
tolC_1	283	302	256	280	23
mqnD	267	338	234	280	53
MTHMO_v1_0847	344	305	184	278	84
MTHMO_v1_0046	296	292	243	277	29
MTHMO_v1_1406	268	288	273	276	10
thrS	255	262	312	276	31
MTHMO_v1_0783	257	308	261	275	29
gltS	242	299	286	275	30
nrfG_2	248	310	268	275	32
mnmA	286	211	328	275	59
MTHMO_v1_1823	261	321	242	274	41
MTHMO_v1_1181	210	264	348	274	70
MTHMO_v1_1199	227	306	285	273	41
MTHMO_v1_1646	337	271	207	272	65

mdlB	272	296	248	272	24
MTHMO_v1_1898	238	327	250	272	48
MTHMO_v1_0102	242	318	252	271	41
MTHMO_v1_1097	258	243	312	271	36
nadK	260	285	264	270	13
rplJ	266	263	280	270	9
nadC	305	232	272	270	37
MTHMO_v1_2285	203	304	301	269	58
accD	262	243	302	269	30
MTHMO_v1_0443	280	280	247	269	19
MTHMO_v1_1687	181	344	281	269	82
MTHMO_v1_0949	291	224	290	268	38
tolQ_2	196	351	257	268	78
aroA	292	246	265	268	23
pfs	284	254	265	268	15
gcvT	255	210	337	267	64
MTHMO_v1_1985	348	286	167	267	92
cirA_3	286	273	241	267	23
tolQ_1	228	276	295	266	35
MTHMO_v1_0977	273	284	240	266	23
MTHMO_v1_2255	306	225	266	266	41
rnhB	318	219	258	265	50
cccA	230	302	263	265	37
MTHMO_v1_0361	231	286	277	265	30
clpX_2	286	279	230	265	31
yycJ	250	279	265	265	15
MTHMO_v1_2260	229	276	289	265	32
phoB	248	330	213	263	60
nrdR	166	317	306	263	84
MTHMO_v1_0317	368	245	177	263	97
aroF	232	310	247	263	41
dnaJ	282	263	243	263	19
icd	317	236	235	262	47
MTHMO_v1_1107	379	246	162	262	109
MTHMO_v1_2049	272	252	262	262	10
MTHMO_v1_0765	294	258	233	261	31
treY	254	302	228	261	38
yidC	260	291	232	261	29
ruvB	247	259	275	260	14
MTHMO_v1_1133	183	282	316	260	69
MTHMO_v1_0644	256	447	70	258	189
MTHMO_v1_1012	171	285	310	255	75

srpH	198	309	259	255	55
ccmC	229	279	252	253	25
fba_1	249	272	239	253	17
MTHMO_v1_1035	275	200	284	253	46
accB	268	206	286	253	42
MTHMO_v1_1400	252	236	271	253	17
MTHMO_v1_0264	240	255	259	251	10
fur	232	247	274	251	22
MTHMO_v1_1131	269	220	264	251	27
fabH	265	252	234	250	15
MTHMO_v1_2118	228	312	211	250	54
nuoE	360	230	161	250	101
MTHMO_v1_0561	200	274	275	250	43
MTHMO_v1_0215	268	254	227	249	21
asd	254	238	257	249	10
MTHMO_v1_1727	245	239	262	249	12
zwf_1	151	337	256	248	94
MTHMO_v1_2136	230	306	208	248	51
MTHMO_v1_2097	232	298	213	248	44
MTHMO_v1_0107	242	226	274	247	24
fmt	208	275	258	247	35
hybA	238	261	241	247	13
pycA	278	254	206	246	36
gadA	231	258	250	246	14
ilvD	245	269	224	246	22
MTHMO_v1_0754	265	261	211	246	30
MTHMO_v1_1816	302	161	273	245	75
MTHMO_v1_0162	243	253	239	245	8
MTHMO_v1_1334	193	281	260	245	46
recA	276	290	169	245	66
MTHMO_v1_0117	307	239	187	244	60
udgB	280	271	179	243	56
MTHMO_v1_0227	127	314	289	243	101
MTHMO_v1_0937	251	220	258	243	21
MTHMO_v1_0540	279	285	165	243	68
purN	257	302	165	241	70
MTHMO_v1_0318	242	278	204	241	37
MTHMO_v1_1322	271	240	211	240	30
MTHMO_v1_1298	209	254	258	240	27
sucA	211	275	229	238	33
MTHMO_v1_0444	196	280	239	238	42
mpl	278	219	218	238	34
MTHMO_v1_2043	236	251	227	238	12

MTHMO_v1_2092	225	309	180	238	65
MTHMO_v1_0674	215	214	284	238	40
gatA	283	210	218	237	40
MTHMO_v1_1952	205	230	273	236	35
atpH	272	272	164	236	63
nadB	186	293	227	235	54
rodA	217	246	242	235	16
MTHMO_v1_0296	271	261	174	235	53
katG_1	204	247	250	234	26
MTHMO_v1_1751	224	254	223	234	18
prfB	285	212	204	234	45
nuoL	188	251	261	233	39
pspE	245	230	225	233	11
MTHMO_v1_1903	256	230	212	232	22
ald	253	247	197	232	31
MTHMO_v1_1656	293	187	216	232	55
MTHMO_v1_1970	196	312	188	232	70
MTHMO_v1_1330	212	230	253	232	20
dhaS	272	280	143	231	77
MTHMO_v1_1621	331	232	130	231	101
arcB	222	236	235	231	8
MTHMO_v1_1885	294	293	106	231	108
MTHMO_v1_2041	206	256	231	231	25
slp_1	228	254	209	230	22
MTHMO_v1_0759	107	205	379	230	138
mtoX	195	274	220	230	40
lpd	207	241	241	230	20
MTHMO_v1_0836	170	354	164	229	108
MTHMO_v1_1424	206	322	159	229	84
fabI	255	195	237	229	31
MTHMO_v1_1188	230	226	229	228	2
dcd	258	200	226	228	29
MTHMO_v1_0548	161	183	340	228	97
hemB	266	205	211	228	34
MTHMO_v1_1600	196	274	212	227	41
ftsK	233	262	186	227	38
MTHMO_v1_1804	225	258	199	227	29
MTHMO_v1_2239	167	294	220	227	64
ftsA	211	241	228	227	15
MTHMO_v1_1021	209	230	241	227	16
MTHMO_v1_0007	159	153	368	226	122
MTHMO_v1_0970	206	233	240	226	18

MTHMO_v1_2286	139	251	289	226	78
MTHMO_v1_2158	215	233	228	225	9
MTHMO_v1_2326	308	177	190	225	72
hisD	215	271	187	225	43
MTHMO_v1_0094	248	220	205	224	22
hprK	180	265	226	224	43
MTHMO_v1_1360	242	249	180	223	38
MTHMO_v1_0032	265	238	166	223	51
MTHMO_v1_2124	222	240	207	223	17
thiE	217	205	247	223	22
pyrB	226	201	242	223	21
MTHMO_v1_0860	190	266	211	222	40
MTHMO_v1_1737	247	231	190	222	29
MTHMO_v1_1155	234	242	185	220	31
MTHMO_v1_0637	214	266	180	220	43
trpB	192	199	267	220	41
MTHMO_v1_0889	106	314	238	219	105
sucD	245	192	221	219	27
speA	201	201	255	219	31
resB	214	268	173	218	47
MTHMO_v1_0619	218	241	195	218	23
MTHMO_v1_1094	140	332	180	217	101
rffG	198	214	238	217	20
MTHMO_v1_0485	214	238	199	217	20
ispD	193	206	248	216	29
MTHMO_v1_0050	187	257	201	215	37
ddlB	252	229	164	215	46
ompA_4	208	235	200	214	18
MTHMO_v1_1620	173	259	211	214	43
MTHMO_v1_0826	182	216	243	214	30
rtcB	192	256	193	214	37
MTHMO_v1_0998	200	213	228	214	14
malP	182	248	209	213	33
MTHMO_v1_0149	215	243	181	213	31
purH	166	260	210	212	47
dnaB	207	221	208	212	7
nadA	201	253	180	211	37
rffH	171	237	222	210	35
lysA	214	196	220	210	13
adhA	266	186	176	209	49
MTHMO_v1_2072	170	213	245	209	38
MTHMO_v1_0555	218	183	227	209	23
msrAB	140	274	214	209	67

MTHMO_v1_0725	129	269	228	209	72
mdh	238	200	187	208	26
MTHMO_v1_1316	184	275	166	208	59
serS	237	229	157	208	44
dapB	226	168	226	206	33
MTHMO_v1_0228	152	207	260	206	54
erg_2	179	205	234	206	28
leuS	213	230	172	205	30
MTHMO_v1_1405	227	223	164	205	35
MTHMO_v1_0766	188	184	242	205	32
MTHMO_v1_0672	168	234	212	205	34
MTHMO_v1_0974	192	251	170	205	42
MTHMO_v1_0379	251	150	211	204	51
apt	209	131	272	204	71
MTHMO_v1_0722	144	234	233	204	52
cysD	166	198	247	204	41
MTHMO_v1_1488	197	207	206	203	5
MTHMO_v1_1967	198	228	183	203	23
carB_1	196	233	179	203	28
MTHMO_v1_0510	164	237	203	202	37
MTHMO_v1_0224	234	216	155	202	41
rpoN	188	190	227	201	22
mscL	173	248	183	201	41
atpE_1	173	264	167	201	54
MTHMO_v1_0530	125	277	200	201	76
MTHMO_v1_2173	186	251	165	201	45
MTHMO_v1_1794	105	284	212	200	90
MTHMO_v1_1491	185	186	230	200	26
lpxA_2	236	247	117	200	72
MTHMO_v1_0081	204	199	196	200	4
MTHMO_v1_2305	177	235	187	200	31
MTHMO_v1_1301	206	219	174	200	23
gnd	231	198	170	200	31
MTHMO_v1_1139	215	177	207	200	20
frdA	195	214	188	199	13
MTHMO_v1_2086	192	208	196	199	8
MTHMO_v1_0196	177	282	136	199	76
MTHMO_v1_1834	218	198	179	198	20
MTHMO_v1_0741	237	197	159	198	39
MTHMO_v1_1117	185	204	205	198	11
MTHMO_v1_2040	191	169	233	198	32
MTHMO_v1_0848	178	226	189	198	25

MTHMO_v1_0025	128	250	215	197	63
MTHMO_v1_0997	244	191	157	197	44
ubiX	202	252	137	197	58
MTHMO_v1_2288	173	240	178	197	37
MTHMO_v1_0990	180	218	192	197	19
acoB	220	219	152	197	39
MTHMO_v1_0541	192	228	170	197	29
fumC	182	162	243	196	42
MTHMO_v1_0877	245	148	193	196	49
MTHMO_v1_1914	199	169	217	195	24
argG	190	214	177	194	19
cysG	219	198	164	193	28
MTHMO_v1_1385	580	0	0	193	335
pqqE	220	199	158	192	31
truB	215	189	171	192	22
guaA	191	190	195	192	3
MTHMO_v1_1283	219	192	163	191	28
proB	156	179	237	191	42
cyoA1	243	187	143	191	50
MTHMO_v1_0600	191	195	187	191	4
exo	212	221	138	190	45
MTHMO_v1_1331	194	189	186	190	4
ttg2C	180	192	197	190	9
MTHMO_v1_0599	229	193	147	189	41
pntAA	203	176	189	189	14
MTHMO_v1_0681	46	297	223	189	129
MTHMO_v1_1570	189	258	117	188	71
MTHMO_v1_0919	174	210	179	188	20
MTHMO_v1_0200	258	129	175	187	65
MTHMO_v1_1356	206	146	209	187	35
ispF	152	261	146	186	65
MTHMO_v1_0347	109	224	225	186	67
atpB_2	159	199	199	186	23
lepA	211	161	185	186	25
emrA_2	192	200	164	185	19
MTHMO_v1_0237	195	126	235	185	55
thiH	148	208	199	185	32
MTHMO_v1_1655	205	174	176	185	17
murE	194	161	199	185	21
MTHMO_v1_0706	153	224	176	184	36
sucC	185	170	194	183	12
trpE	172	183	191	182	10
MTHMO_v1_0658	190	174	182	182	8

MTHMO_v1_0477	222	149	174	182	37
acrA_3	200	193	151	181	27
MTHMO_v1_1709	173	210	161	181	25
MTHMO_v1_2272	239	161	141	180	51
MTHMO_v1_1551	179	197	165	180	16
dapA	173	224	144	180	40
hypB	173	203	159	178	23
MTHMO_v1_0737	172	194	168	178	14
ligA	181	190	162	178	14
MTHMO_v1_1573	161	207	161	177	27
MTHMO_v1_1591	87	276	167	177	95
MTHMO_v1_2199	157	181	190	176	17
ftsQ	132	173	223	176	46
glgA_1	186	196	145	176	27
MTHMO_v1_1929	251	184	91	175	81
MTHMO_v1_0641	150	202	173	175	26
rimO	178	208	139	175	34
yhdN	150	178	197	175	23
MTHMO_v1_0463	198	140	186	175	31
cyoB1	219	159	145	174	39
MTHMO_v1_2020	220	186	115	174	53
MTHMO_v1_1908	180	182	160	174	12
MTHMO_v1_0084	176	203	141	173	31
MTHMO_v1_0232	179	207	131	172	38
MTHMO_v1_0748	174	162	178	172	8
ppk	168	176	171	172	4
rpsC	310	115	89	171	120
MTHMO_v1_1372	154	168	192	171	19
MTHMO_v1_1393	178	180	154	171	14
MTHMO_v1_1801	117	163	233	171	59
adk	197	192	123	171	41
nrfG_1	170	141	200	170	30
MTHMO_v1_0756	174	224	113	170	55
MTHMO_v1_0088	198	144	168	170	27
acs	208	153	148	170	33
MTHMO_v1_0216	128	145	236	170	58
nuoM	128	207	172	169	40
MTHMO_v1_0971	194	186	125	168	38
tyrS	195	183	127	168	36
MTHMO_v1_1719	179	170	155	168	12
MTHMO_v1_1326	192	133	179	168	31
pqqC	169	241	94	168	74

ppx	199	183	122	168	41
argD_2	158	163	182	168	13
MTHMO_v1_0751	259	149	94	168	84
fabZ	168	192	142	167	25
hemA	157	133	211	167	40
MTHMO_v1_1593	81	205	214	167	74
MTHMO_v1_0109	155	149	195	167	25
MTHMO_v1_0772	152	176	172	166	13
MTHMO_v1_1302	121	294	83	166	113
nuoG	151	209	138	166	38
glgP	169	183	145	166	19
MTHMO_v1_1854	187	220	90	166	68
folD	136	216	145	166	44
MTHMO_v1_0288	226	135	136	166	52
yhhT	165	173	159	166	7
xoxF2	160	176	160	165	9
pgsA	151	171	174	165	13
MTHMO_v1_1836	195	161	139	165	28
MTHMO_v1_0108	180	228	87	165	72
suhB	153	168	172	164	10
murA	165	133	194	164	31
gppA	153	159	179	164	14
acrB_2	176	180	136	164	25
MTHMO_v1_1598	167	144	180	164	18
MTHMO_v1_0051	159	179	153	163	14
murF	185	191	112	163	44
acrB_1	147	184	157	163	19
bioB	110	165	211	162	51
MTHMO_v1_0782	142	189	152	161	25
dppB	179	190	113	161	42
MTHMO_v1_1927	178	184	118	160	36
MTHMO_v1_0203	129	163	188	160	30
MTHMO_v1_2188	149	197	133	160	33
MTHMO_v1_1078	104	192	182	160	48
lysS	161	176	141	159	17
MTHMO_v1_1383	170	105	202	159	50
obg	161	127	189	159	31
kch	168	165	143	159	13
MTHMO_v1_2310	184	107	185	159	45
pgm	187	171	117	159	37
purU	156	175	143	158	16
MTHMO_v1_0842	149	159	166	158	8
MTHMO_v1_0427	127	141	204	158	41

fabG_2	161	166	146	157	10
MTHMO_v1_1946	181	149	142	157	21
MTHMO_v1_1860	155	177	140	157	18
MTHMO_v1_1638	181	176	114	157	37
argH	135	179	157	157	22
MTHMO_v1_2098	75	187	209	157	72
moaA	157	192	118	156	37
cutA	100	192	174	156	49
MTHMO_v1_0448	156	144	165	155	10
rfaF	117	153	194	155	39
MTHMO_v1_1154	181	159	123	154	29
MTHMO_v1_2031	155	157	152	154	2
MTHMO_v1_0866	120	204	138	154	44
MTHMO_v1_0588	121	210	132	154	49
MTHMO_v1_1293	127	224	110	154	61
MTHMO_v1_0141	162	151	149	154	7
MTHMO_v1_1082	161	172	128	154	23
mqnA	174	156	132	154	21
MTHMO_v1_0552	202	114	145	154	44
MTHMO_v1_0378	141	175	140	152	20
atpA_1	145	152	160	152	8
glgB	128	162	166	152	21
MTHMO_v1_1611	127	156	172	152	22
norB	137	176	141	152	22
mesJ	111	196	147	151	43
MTHMO_v1_1865	163	179	112	151	35
MTHMO_v1_1478	129	165	159	151	19
MTHMO_v1_0351	173	176	104	151	41
MTHMO_v1_2053	160	127	165	151	21
mraY	207	145	99	150	54
MTHMO_v1_2335	170	90	191	150	53
MTHMO_v1_0369	144	147	159	150	8
gdhA	192	136	123	150	36
rfaG_3	177	141	133	150	23
MTHMO_v1_0006	118	151	182	150	32
MTHMO_v1_0132	152	175	122	150	27
trpS	160	122	167	150	24
MTHMO_v1_2306	167	121	161	149	25
fabG_1	151	151	145	149	3
MTHMO_v1_0009	216	127	104	149	59
tldD_1	135	176	135	149	24
apc4_2	178	131	136	149	26

MTHMO_v1_1121	174	156	116	148	30
MTHMO_v1_0345	153	151	142	148	6
MTHMO_v1_0612	114	149	181	148	33
MTHMO_v1_2327	158	101	182	147	42
MTHMO_v1_0038	171	143	126	147	23
MTHMO_v1_1711	171	154	113	146	30
pitA	125	166	148	146	21
MTHMO_v1_1096	136	163	140	146	14
MTHMO_v1_2308	151	132	155	146	12
MTHMO_v1_0938	181	119	137	146	32
ppc	194	158	86	146	55
atpF_1	161	138	138	146	13
MTHMO_v1_0527	161	153	122	146	21
murD	132	156	146	145	12
MTHMO_v1_1320	96	166	173	145	43
MTHMO_v1_1280	165	115	153	144	26
MTHMO_v1_0248	142	141	147	144	3
hypF	179	127	125	143	30
cbpA	132	152	147	143	10
MTHMO_v1_2317	182	129	117	143	35
cas_3	115	159	155	143	24
MTHMO_v1_0928	81	224	124	143	73
pabA	197	93	137	142	52
MTHMO_v1_2055	136	153	137	142	10
MTHMO_v1_0594	152	135	139	142	9
MTHMO_v1_0921	141	153	132	142	10
MTHMO_v1_1341	157	126	143	142	16
hisC_2	130	205	91	142	58
phoD	198	145	81	141	58
lonB	141	153	130	141	11
MTHMO_v1_0787	154	132	136	141	12
panB	136	163	123	141	21
trpA	145	167	110	141	28
MTHMO_v1_0988	140	112	169	140	28
MTHMO_v1_0441	132	129	161	140	18
mgtA	136	151	135	140	9
MTHMO_v1_2298	136	163	122	140	21
accA	123	167	131	140	23
MTHMO_v1_0085	145	114	162	140	24
MTHMO_v1_1191	196	97	128	140	51
MTHMO_v1_1407	146	107	165	140	29
MTHMO_v1_1503	114	163	141	140	24
aroE	160	132	126	139	18

MTHMO_v1_2117	111	178	129	139	35
dapL	124	143	150	139	14
MTHMO_v1_0673	77	174	166	139	54
MTHMO_v1_1000	147	169	102	139	34
mnmG	137	157	122	139	18
MTHMO_v1_1926	173	193	50	139	78
moaC	156	124	135	138	16
ftsH_1	130	159	125	138	19
MTHMO_v1_0534	129	144	140	138	8
MTHMO_v1_0358	161	140	112	138	24
MTHMO_v1_1835	77	133	203	138	63
carB_2	108	179	125	137	37
MTHMO_v1_0627	134	95	182	137	44
hyfE	131	134	146	137	8
MTHMO_v1_1817	135	162	114	137	24
aroB	130	148	131	136	10
imp	132	153	124	136	15
thiS	94	134	181	136	44
hisK	135	161	113	136	24
MTHMO_v1_0732	115	135	157	136	21
carA	148	139	120	136	14
MTHMO_v1_0346	74	119	214	136	72
emrA_1	84	135	188	136	52
MTHMO_v1_0048	160	147	100	136	32
tolC_2	128	176	101	135	38
MTHMO_v1_0596	114	126	164	135	26
MTHMO_v1_0575	0	244	160	135	124
MTHMO_v1_2137	101	108	195	135	52
MTHMO_v1_0833	147	111	146	135	20
MTHMO_v1_1710	126	156	122	135	19
MTHMO_v1_0303	123	120	160	134	22
erpA	73	260	70	134	109
glnD	135	137	130	134	4
cirA_1	136	141	124	134	9
MTHMO_v1_2195	92	86	221	133	76
MTHMO_v1_2052	221	101	76	133	78
glgC	107	142	148	132	22
mazG	107	114	175	132	37
MTHMO_v1_1994	116	161	119	132	25
thrB	107	132	157	132	25
hpt	196	98	100	132	56
valS	122	131	139	131	9

MTHMO_v1_0111	108	164	120	131	29
def	106	138	147	131	22
ileS	116	129	147	131	16
MTHMO_v1_0458	121	167	104	131	32
ompA_2	144	108	139	130	19
pyrC	131	150	110	130	20
glgE	148	122	120	130	16
MTHMO_v1_1118	111	150	128	130	19
MTHMO_v1_0623	135	115	139	130	13
MTHMO_v1_0886	119	91	178	129	44
copA	145	116	127	129	14
MTHMO_v1_1292	120	119	148	129	17
MTHMO_v1_1857	138	88	160	129	37
csd	82	163	142	129	42
sdhB	129	133	124	129	4
nuoH	83	167	136	129	43
purC	148	138	100	129	26
argC	136	131	118	129	9
atpE_2	127	136	123	129	7
bglB	147	106	132	128	20
gcvH	101	107	177	128	43
feoB	133	127	125	128	4
MTHMO_v1_0763	105	111	168	128	35
hypD	131	127	126	128	3
MTHMO_v1_0368	175	124	84	128	45
MTHMO_v1_0168	152	85	146	128	37
MTHMO_v1_1327	90	157	136	127	34
nuoK	87	150	146	127	35
MTHMO_v1_0777	94	155	133	127	30
mlaF	102	140	139	127	22
MTHMO_v1_2307	169	96	116	127	37
proC	141	125	113	127	14
purQ	146	92	141	126	30
tatC	133	133	112	126	12
MTHMO_v1_1992	129	138	111	126	14
uvrB	126	136	115	126	10
MTHMO_v1_1076	150	102	125	126	24
ygfA	135	103	139	126	20
cirA_5	127	125	125	126	1
prfA	133	110	134	126	14
yitJ	154	135	88	126	34
MTHMO_v1_1303	130	160	87	125	37
MTHMO_v1_2356	127	158	88	125	35

MTHMO_v1_0034	100	156	117	125	29
uvrC	101	154	118	124	27
murB_1	119	116	134	123	10
iscS_2	161	80	127	123	41
xerD	168	109	91	123	40
MTHMO_v1_0755	49	144	174	123	65
sua	163	104	100	122	35
MTHMO_v1_1532	129	127	110	122	11
dapb	117	141	109	122	17
nadD	143	106	117	122	19
ompA_1	129	131	105	122	15
MTHMO_v1_0036	110	117	138	122	14
MTHMO_v1_0781	82	145	139	122	35
MTHMO_v1_1647	181	112	72	122	55
MTHMO_v1_2004	130	149	86	122	32
MTHMO_v1_2026	124	124	116	121	5
MTHMO_v1_1340	97	112	155	121	30
proA	117	130	117	121	7
MTHMO_v1_0944	162	120	81	121	40
MTHMO_v1_1190	110	122	130	121	10
MTHMO_v1_2001	148	113	102	121	24
MTHMO_v1_2214	129	138	95	121	23
thrC	126	134	103	121	16
MTHMO_v1_1413	176	108	78	120	50
uppS	112	104	144	120	21
cysS	120	125	115	120	5
MTHMO_v1_1492	186	62	112	120	62
MTHMO_v1_1594	136	190	33	120	80
MTHMO_v1_0549	127	146	86	120	31
ppa	162	113	84	119	39
MTHMO_v1_0727	104	129	125	119	13
MTHMO_v1_2227	156	96	104	119	33
MTHMO_v1_1381	111	125	118	118	7
mog	134	129	90	118	24
kdpD	92	129	131	117	22
MTHMO_v1_1919	99	101	152	117	30
MTHMO_v1_0495	137	133	79	117	32
MTHMO_v1_1274	156	69	125	116	44
MTHMO_v1_0061	71	123	154	116	42
pth	145	77	127	116	35
MTHMO_v1_0960	117	118	113	116	3
ackA	131	143	74	116	37

pheT	107	107	134	116	16
galE	66	161	121	116	48
atoC	112	114	122	116	5
ribE	111	130	107	116	12
MTHMO_v1_2114	109	116	122	116	7
ribF	105	115	126	115	11
pqqB	133	117	96	115	19
fdhD	99	145	102	115	26
MTHMO_v1_1662	121	101	124	115	13
MTHMO_v1_1151	131	108	105	115	14
MTHMO_v1_0195	103	102	139	115	21
MTHMO_v1_0680	0	148	195	114	102
MTHMO_v1_1848	95	106	142	114	24
MTHMO_v1_0151	139	82	121	114	29
MTHMO_v1_1358	97	125	119	114	15
MTHMO_v1_0272	101	136	104	114	19
trpC_2	109	127	105	114	12
MTHMO_v1_1645	153	88	98	113	35
apc3	141	97	101	113	25
MTHMO_v1_2100	100	143	96	113	26
ffh	116	96	126	113	15
MTHMO_v1_0292	55	153	130	113	51
MTHMO_v1_1073	81	129	127	113	27
MTHMO_v1_1604	100	147	90	112	30
lolE	134	113	90	112	22
MTHMO_v1_1452	104	132	100	112	18
MTHMO_v1_1738	150	103	82	112	35
MTHMO_v1_0447	138	89	107	111	25
MTHMO_v1_1748	66	141	127	111	40
MTHMO_v1_1277	0	0	333	111	192
dnaX	81	147	104	111	34
araC	100	99	131	110	18
MTHMO_v1_1092	112	131	86	110	23
MTHMO_v1_1861	163	99	67	109	49
MTHMO_v1_1616	107	113	107	109	3
MTHMO_v1_1659	124	121	80	109	25
dnaG	111	108	107	108	2
MTHMO_v1_0611	144	114	67	108	39
oppC	105	139	79	108	30
MTHMO_v1_0300	86	116	121	108	19
MTHMO_v1_1601	141	75	106	107	33
xseA	131	92	99	107	21
MTHMO_v1_1750	106	121	94	107	14

rlmN	95	82	143	107	32
MTHMO_v1_0439	58	129	133	107	42
kdsA	83	124	112	106	21
MTHMO_v1_0757	129	122	69	106	33
MTHMO_v1_0210	136	69	111	106	34
MTHMO_v1_0245	110	115	92	106	12
MTHMO_v1_1913	92	109	114	105	11
recD	91	122	102	105	16
MTHMO_v1_0229	154	129	32	105	64
MTHMO_v1_2366	126	90	98	105	19
MTHMO_v1_0861	106	119	89	105	15
MTHMO_v1_2010	96	132	85	104	25
glgX	77	111	124	104	24
hypA	98	83	132	104	25
MTHMO_v1_1104	99	90	123	104	17
MTHMO_v1_0139	75	143	93	104	35
MTHMO_v1_1595	99	105	107	104	4
MTHMO_v1_0415	71	126	114	104	29
MTHMO_v1_0535	112	91	108	103	11
tadA	120	99	90	103	15
MTHMO_v1_0986	108	133	68	103	33
purB	107	128	74	103	27
ptsI	118	110	81	103	20
wcaA_2	115	114	79	103	20
sdhC	106	118	84	103	18
MTHMO_v1_2341	86	128	92	102	22
hyfF	111	81	115	102	19
MTHMO_v1_0648	86	91	130	102	24
MTHMO_v1_0664	116	113	77	102	22
mfd	106	113	87	102	14
MTHMO_v1_0199	107	65	134	102	35
MTHMO_v1_0375	97	110	99	102	7
gph	51	126	128	102	44
truA	122	81	102	102	20
MTHMO_v1_1315	64	119	123	102	33
MTHMO_v1_1550	157	92	56	102	51
MTHMO_v1_0082	86	119	99	102	17
MTHMO_v1_2330	118	98	88	101	15
MTHMO_v1_2032	105	85	114	101	14
MTHMO_v1_0912	156	83	64	101	48
MTHMO_v1_1084	120	99	84	101	18
MTHMO_v1_0767	96	133	74	101	30

MTHMO_v1_0126	124	79	100	101	22
MTHMO_v1_1090	57	135	110	101	40
MTHMO_v1_1291	78	109	115	101	20
pstS	130	103	69	101	31
MTHMO_v1_0900	135	36	130	100	56
MTHMO_v1_0312	114	106	81	100	17
ctaB	77	94	129	100	26
MTHMO_v1_1135	110	84	105	100	14
pmoA	123	98	79	100	22
purK	124	107	68	100	29
psd	73	105	120	99	24
MTHMO_v1_0984	139	71	87	99	36
MTHMO_v1_1248	74	79	143	99	38
MTHMO_v1_2301	105	111	79	98	17
MTHMO_v1_1765	105	96	94	98	6
ubiA	78	103	112	98	18
ttg2B	65	112	117	98	29
MTHMO_v1_1408	97	111	85	97	13
MTHMO_v1_0942	95	139	58	97	41
rhlE	102	95	93	97	5
gcvP	79	115	96	97	18
MTHMO_v1_0070	87	102	101	97	8
erg_1	70	128	91	96	29
mrp	77	88	123	96	24
MTHMO_v1_2342	95	84	109	96	12
putP	118	102	67	96	26
gntK	83	116	87	95	18
MTHMO_v1_1034	115	88	83	95	17
MTHMO_v1_1873	123	83	79	95	24
MTHMO_v1_1630	51	136	98	95	42
glnE	106	90	89	95	9
MTHMO_v1_1991	38	101	145	95	54
trpC_1	132	96	55	94	38
paeR7IM	76	100	107	94	16
upp	122	102	58	94	32
MTHMO_v1_0570	117	87	79	94	20
pstB	98	82	102	94	10
ribH	131	87	63	94	35
MTHMO_v1_1565	122	83	75	94	25
MTHMO_v1_1209	94	67	120	94	27
lexA	88	129	63	93	33
MTHMO_v1_0801	116	112	50	93	37
cimA	109	92	77	93	16

MTHMO_v1_0959	96	89	92	93	3
aroC	92	133	53	93	40
ctaA	68	121	89	92	27
MTHMO_v1_2152	121	97	58	92	32
MTHMO_v1_0697	49	156	71	92	57
MTHMO_v1_0620	77	99	100	92	13
MTHMO_v1_0544	102	82	91	92	10
MTHMO_v1_0470	61	168	47	92	66
MTHMO_v1_2073	86	103	85	91	10
oppA	100	73	100	91	16
MTHMO_v1_0135	101	81	89	91	10
MTHMO_v1_1851	69	110	93	90	21
der	99	93	77	90	11
MTHMO_v1_0707	114	94	61	90	26
MTHMO_v1_0371	121	68	80	90	28
ccoN	110	93	66	89	22
MTHMO_v1_0426	41	88	139	89	49
MTHMO_v1_1020	80	85	103	89	12
ebrB	102	87	79	89	12
mobA	122	87	59	89	32
MTHMO_v1_2141	99	84	84	89	9
MTHMO_v1_0967	43	168	55	89	69
MTHMO_v1_0370	58	107	102	89	27
MTHMO_v1_1208	94	75	97	89	12
MTHMO_v1_0542	76	80	109	88	18
dnaE_1	97	88	80	88	8
MTHMO_v1_0572	62	120	83	88	30
dppC	87	86	90	88	2
MTHMO_v1_0628	95	106	62	88	23
atpG_1	98	93	73	88	13
hypE	90	93	81	88	6
MTHMO_v1_2174	89	80	94	88	7
MTHMO_v1_2029	66	102	95	88	19
hisA	110	83	70	88	20
MTHMO_v1_1343	68	85	109	87	21
leuB	80	105	77	87	15
MTHMO_v1_0983	94	83	84	87	6
MTHMO_v1_1088	108	83	70	87	20
MTHMO_v1_0488	93	124	45	87	40
cirA_8	91	91	79	87	7
galT	72	108	82	87	19
degT	116	86	59	87	29

MTHMO_v1_1440	73	82	105	87	17
MTHMO_v1_0414	90	98	71	87	14
MTHMO_v1_0992	124	61	74	86	34
tonB_4	97	69	93	86	15
MTHMO_v1_0176	59	125	75	86	34
dacC_1	69	86	104	86	17
MTHMO_v1_2240	88	84	84	85	2
mltE	95	101	61	85	22
MTHMO_v1_2193	76	83	97	85	11
xenB_1	25	40	191	85	92
purM	73	81	101	85	15
ftsW	75	123	56	85	35
MTHMO_v1_0287	80	73	99	84	13
MTHMO_v1_1872	100	94	59	84	22
MTHMO_v1_1342	152	81	18	84	67
MTHMO_v1_2302	61	73	117	84	30
smtA_1	102	79	71	84	16
MTHMO_v1_0022	97	83	71	84	13
tldD_2	92	102	56	83	25
MTHMO_v1_0834	110	76	64	83	24
thyA	67	94	89	83	14
MTHMO_v1_0950	55	103	93	83	25
pncA_1	49	130	71	83	42
alaS	85	78	87	83	5
MTHMO_v1_0595	100	92	58	83	22
MTHMO_v1_1332	75	80	93	83	9
MTHMO_v1_1265	117	47	85	83	35
MTHMO_v1_1578	120	59	69	83	33
MTHMO_v1_2233	79	69	100	83	16
rfaG_1	99	100	50	83	28
MTHMO_v1_2077	91	82	75	83	8
MTHMO_v1_1008	70	111	67	83	25
MTHMO_v1_1189	73	99	76	83	14
MTHMO_v1_2297	77	80	89	82	6
tonB_1	82	115	49	82	33
MTHMO_v1_2142	71	95	80	82	12
MTHMO_v1_2074	81	86	78	82	4
MTHMO_v1_0024	72	64	108	82	23
MTHMO_v1_0645	93	107	45	82	33
pstC	103	79	62	81	21
MTHMO_v1_1639	93	96	56	81	22
nusB	71	91	82	81	10
murG	79	100	64	81	18

MTHMO_v1_0350	61	117	66	81	31
MTHMO_v1_1703	72	110	61	81	26
pntB	67	74	101	80	18
pqqG	67	93	80	80	13
uppP	100	76	64	80	18
MTHMO_v1_1559	69	97	73	80	15
purD	106	80	53	80	26
folP	78	72	89	80	9
MTHMO_v1_0762	105	67	66	80	22
MTHMO_v1_2021	80	49	110	79	31
argS	89	72	78	79	9
pheS	84	85	68	79	10
MTHMO_v1_1287	90	78	68	79	11
surA	87	64	84	79	12
MTHMO_v1_0663	70	78	87	78	9
MTHMO_v1_2253	54	104	77	78	25
MTHMO_v1_0550	69	0	166	78	83
MTHMO_v1_0821	78	65	92	78	14
MTHMO_v1_1982	53	105	76	78	26
MTHMO_v1_0266	104	87	43	78	32
MTHMO_v1_0646	79	84	71	78	6
MTHMO_v1_1725	91	53	88	78	21
MTHMO_v1_0372	44	70	119	78	38
MTHMO_v1_1699	77	100	55	77	22
MTHMO_v1_2225	63	78	91	77	14
MTHMO_v1_0948	47	124	60	77	42
MTHMO_v1_0739	90	80	58	76	17
caiC	94	77	57	76	18
MTHMO_v1_0668	75	88	65	76	11
MTHMO_v1_0604	91	68	70	76	13
ruvA	66	151	11	76	71
MTHMO_v1_0617	63	83	81	76	11
MTHMO_v1_1382	52	74	101	76	24
MTHMO_v1_1890	82	66	79	76	9
MTHMO_v1_0071	73	71	83	76	7
MTHMO_v1_1362	136	66	24	75	57
MTHMO_v1_1006	66	72	88	75	11
MTHMO_v1_1583	60	64	100	75	22
nifX	76	89	59	75	15
MTHMO_v1_0453	53	103	68	74	26
hisF	86	78	58	74	15
MTHMO_v1_1354	56	59	107	74	29

MTHMO_v1_2264	67	75	80	74	7
MTHMO_v1_0063	89	79	52	73	20
MTHMO_v1_1846	52	60	108	73	30
MTHMO_v1_1805	77	77	65	73	7
MTHMO_v1_1116	63	67	87	73	13
MTHMO_v1_0660	80	77	61	73	10
MTHMO_v1_0831	84	67	65	72	11
MTHMO_v1_1178	104	55	57	72	28
glmM	64	73	80	72	8
MTHMO_v1_0881	79	63	73	72	8
MTHMO_v1_1077	90	75	50	72	21
modB	100	86	29	72	38
ccoO	91	91	33	72	34
kdpA	53	106	55	71	30
MTHMO_v1_0471	73	0	141	71	70
MTHMO_v1_1764	82	60	71	71	11
MTHMO_v1_0305	84	65	63	71	12
MTHMO_v1_1634	88	57	66	71	16
kdsB	82	77	53	71	16
MTHMO_v1_0354	72	74	65	70	5
MTHMO_v1_1375	70	74	67	70	4
MTHMO_v1_0127	60	46	103	70	30
MTHMO_v1_2096	47	58	105	70	31
MTHMO_v1_0008	44	94	71	70	25
MTHMO_v1_0405	74	99	36	70	32
miaB	76	67	65	69	6
hisH	76	58	73	69	10
cysH	77	73	58	69	10
MTHMO_v1_0800	66	64	77	69	7
MTHMO_v1_1686	109	58	38	69	37
MTHMO_v1_1676	81	61	65	69	11
MTHMO_v1_1128	74	55	77	69	12
cusR	116	52	37	68	42
MTHMO_v1_2219	84	49	72	68	18
MTHMO_v1_1608	58	77	70	68	10
MTHMO_v1_0246	72	52	80	68	14
MTHMO_v1_1011	58	56	90	68	19
MTHMO_v1_0455	27	63	114	68	43
MTHMO_v1_1392	88	31	85	68	32
hemL	67	91	45	68	23
hupQ	72	85	46	68	20
MTHMO_v1_1977	71	80	51	68	15
MTHMO_v1_0587	91	86	25	67	37

MTHMO_v1_0410	114	51	37	67	41
MTHMO_v1_0830	58	101	42	67	30
MTHMO_v1_0207	83	53	64	67	15
MTHMO_v1_2134	52	65	84	67	16
MTHMO_v1_0086	63	69	68	67	3
curA	80	75	45	67	19
lipA	77	49	74	66	15
MTHMO_v1_0267	66	70	63	66	3
MTHMO_v1_0173	70	89	40	66	25
MTHMO_v1_0616	60	77	62	66	10
MTHMO_v1_0010	83	70	46	66	19
oppB	58	62	77	66	10
rnhC	43	99	54	65	30
MTHMO_v1_1039	75	77	44	65	19
nirK	38	72	86	65	24
murQ	79	79	38	65	24
MTHMO_v1_1660	70	37	89	65	26
MTHMO_v1_1704	43	71	82	65	20
merR	93	64	39	65	27
MTHMO_v1_0330	45	107	43	65	37
pmoA1	79	74	42	65	20
rnc	55	87	53	65	19
MTHMO_v1_0306	45	82	67	64	19
sul	78	57	58	64	12
mnmE	47	87	59	64	21
MTHMO_v1_1348	88	70	35	64	27
recR	67	83	43	64	20
MTHMO_v1_2190	46	68	78	64	16
rsmI	84	67	41	64	22
MTHMO_v1_0543	68	82	42	64	20
MTHMO_v1_1024	64	74	53	64	10
pncB	90	43	58	63	24
cas_4	66	64	60	63	3
tolC_5	48	76	66	63	14
MTHMO_v1_1313	51	69	70	63	11
nth	61	70	58	63	6
suox	36	70	83	63	25
MTHMO_v1_2222	76	72	41	63	19
MTHMO_v1_2093	108	62	19	63	44
MTHMO_v1_1195	82	62	45	63	18
MTHMO_v1_0910	22	104	62	63	41
MTHMO_v1_0208	28	60	99	62	36

MTHMO_v1_0446	32	86	68	62	27
MTHMO_v1_0438	37	97	53	62	31
MTHMO_v1_0825	69	51	66	62	9
czcA_1	59	57	71	62	7
MTHMO_v1_0832	80	60	46	62	17
MTHMO_v1_0621	60	62	64	62	2
lpxA_1	8	72	106	62	49
MTHMO_v1_0490	48	57	80	62	17
MTHMO_v1_0144	57	59	69	62	6
MTHMO_v1_1296	77	41	66	62	19
MTHMO_v1_0472	52	96	37	62	31
MTHMO_v1_1777	67	36	81	61	23
MTHMO_v1_1635	58	54	70	61	8
MTHMO_v1_0685	78	58	45	60	17
mntH	64	68	47	60	11
MTHMO_v1_1707	87	40	52	60	24
MTHMO_v1_1741	96	68	15	60	41
MTHMO_v1_1412	78	71	30	59	26
tsaD	57	74	48	59	13
MTHMO_v1_1220	34	80	64	59	24
MTHMO_v1_2273	44	41	94	59	30
MTHMO_v1_2314	52	77	50	59	15
MTHMO_v1_1708	39	62	75	59	18
arnT_2	52	49	75	59	14
MTHMO_v1_0966	42	87	46	58	25
MTHMO_v1_1221	74	53	48	58	14
ymdB_1	49	66	59	58	8
MTHMO_v1_2121	95	32	46	58	33
MTHMO_v1_0192	86	39	47	58	25
MTHMO_v1_1093	20	75	77	57	32
MTHMO_v1_0828	57	66	49	57	8
MTHMO_v1_2135	34	71	66	57	20
MTHMO_v1_0951	53	68	51	57	10
MTHMO_v1_1338	43	46	82	57	22
MTHMO_v1_1269	0	93	77	57	50
MTHMO_v1_0524	55	54	60	57	3
MTHMO_v1_1641	65	56	48	56	8
MTHMO_v1_0539	72	45	52	56	14
MTHMO_v1_1612	70	53	45	56	13
MTHMO_v1_1308	46	69	53	56	12
MTHMO_v1_0956	60	50	58	56	6
MTHMO_v1_1446	62	74	32	56	22
MTHMO_v1_1212	26	41	100	56	39

wcaA_1	54	43	69	56	13
MTHMO_v1_1785	82	65	20	56	32
MTHMO_v1_1010	16	119	31	55	56
MTHMO_v1_1767	50	63	54	55	7
MTHMO_v1_1445	17	100	49	55	42
MTHMO_v1_0234	82	58	26	55	28
MTHMO_v1_2300	50	56	60	55	5
MTHMO_v1_0464	64	40	61	55	13
arnT_1	55	44	65	55	10
MTHMO_v1_1409	47	58	59	55	6
MTHMO_v1_0069	78	62	24	55	28
rbcR	67	72	23	54	27
traJ	54	57	51	54	3
MTHMO_v1_1462	47	56	57	53	5
coaD	53	43	64	53	11
MTHMO_v1_0013	47	58	55	53	6
MTHMO_v1_1357	89	55	14	53	38
proP_2	48	63	46	52	9
MTHMO_v1_0015	45	48	65	52	11
MTHMO_v1_1386	27	130	0	52	69
MTHMO_v1_0696	75	24	58	52	26
MTHMO_v1_1162	26	82	49	52	28
lpxK	48	62	46	52	9
MTHMO_v1_1535	57	30	69	52	20
MTHMO_v1_2159	20	21	115	52	54
pyrF	77	51	28	52	24
MTHMO_v1_0850	40	64	51	51	12
MTHMO_v1_0363	65	43	47	51	12
MTHMO_v1_1468	51	55	49	51	3
nifK	51	54	49	51	3
manB	63	39	51	51	12
iscS_1	61	44	48	51	9
MTHMO_v1_2296	45	57	51	51	6
MTHMO_v1_1760	32	65	56	51	17
MTHMO_v1_1091	40	82	31	51	27
MTHMO_v1_0569	77	23	53	51	27
MTHMO_v1_1466	71	53	27	51	22
MTHMO_v1_0770	54	61	37	51	13
MTHMO_v1_1883	59	63	29	50	19
xanB	56	63	32	50	16
MTHMO_v1_0943	61	24	66	50	23
MTHMO_v1_2132	69	31	50	50	19

MTHMO_v1_0806	30	63	57	50	18
panC	39	66	45	50	14
cfxQ	0	149	0	50	86
MTHMO_v1_1266	51	27	70	50	22
MTHMO_v1_1642	62	39	47	49	11
glcD_1	61	47	40	49	10
cysC	77	33	37	49	24
purL	41	57	48	49	8
MTHMO_v1_0775	66	59	21	49	24
MTHMO_v1_0880	69	32	45	49	19
MTHMO_v1_0810	52	56	38	49	10
MTHMO_v1_0191	25	74	47	49	25
pdxJ	54	48	43	49	5
MTHMO_v1_0467	54	14	77	49	32
MTHMO_v1_0878	37	37	71	48	20
MTHMO_v1_2262	52	47	45	48	4
udg	34	67	44	48	17
MTHMO_v1_1582	32	74	38	48	23
MTHMO_v1_1295	25	72	47	48	24
nifD	50	51	44	48	4
katG_2	31	56	56	48	14
MTHMO_v1_2039	79	34	30	48	27
MTHMO_v1_1206	96	0	46	47	48
MTHMO_v1_0799	46	70	25	47	22
silA	50	47	44	47	3
mutL	37	50	54	47	9
MTHMO_v1_0459	46	29	67	47	19
folK	54	87	0	47	44
pstA	22	55	64	47	22
opgG	57	46	38	47	9
MTHMO_v1_1962	39	48	54	47	7
MTHMO_v1_1122	61	33	46	47	14
MTHMO_v1_2140	36	41	64	47	15
MTHMO_v1_1373	18	122	0	47	66
MTHMO_v1_2303	45	34	61	47	14
merP	22	35	83	46	32
alr	54	29	57	46	15
MTHMO_v1_0172	34	49	55	46	11
MTHMO_v1_1335	66	35	36	46	18
kdpB	36	45	57	46	10
MTHMO_v1_0516	70	0	68	46	40
MTHMO_v1_0476	45	58	35	46	12
MTHMO_v1_0367	42	56	40	46	9

MTHMO_v1_0852	67	29	41	46	19
MTHMO_v1_0270	70	34	34	46	21
MTHMO_v1_1268	53	85	0	46	43
MTHMO_v1_1698	28	61	49	46	17
lnt	53	37	47	46	8
MTHMO_v1_1607	37	55	45	46	9
MTHMO_v1_2338	48	22	66	45	22
MTHMO_v1_0404	0	85	51	45	43
arnT_3	51	36	49	45	8
MTHMO_v1_1475	40	64	32	45	16
MTHMO_v1_1166	50	45	40	45	5
MTHMO_v1_0352	46	58	31	45	14
trmB	73	22	40	45	26
MTHMO_v1_1648	0	78	56	45	40
MTHMO_v1_1319	46	39	49	45	5
MTHMO_v1_0422	39	69	25	45	23
gloB_2	73	50	10	44	32
MTHMO_v1_0827	38	20	74	44	27
acrA_1	48	47	38	44	5
MTHMO_v1_1285	69	64	0	44	38
MTHMO_v1_0434	48	38	46	44	5
MTHMO_v1_1126	43	19	70	44	25
MTHMO_v1_1282	66	35	32	44	19
MTHMO_v1_0239	111	20	0	44	59
MTHMO_v1_1210	0	76	55	44	39
MTHMO_v1_2331	48	60	23	44	19
MTHMO_v1_2133	43	46	42	44	2
ybhR	36	42	52	43	8
MTHMO_v1_1564	29	44	56	43	14
treZ	55	49	25	43	16
MTHMO_v1_0771	36	48	44	43	6
MTHMO_v1_1728	36	66	26	42	21
glpK	31	57	39	42	13
MTHMO_v1_1032	49	37	41	42	6
ompA_3	98	15	13	42	48
MTHMO_v1_1070	41	52	33	42	10
lgt	50	44	32	42	9
MTHMO_v1_0586	46	46	34	42	7
MTHMO_v1_1531	31	50	45	42	10
MTHMO_v1_1389	57	30	38	42	14
MTHMO_v1_0271	40	38	46	42	4
MTHMO_v1_1230	27	46	52	42	13

MTHMO_v1_1054	47	25	53	42	15
MTHMO_v1_0275	55	40	29	41	13
MTHMO_v1_1557	46	58	20	41	19
glcD_2	30	39	54	41	12
MTHMO_v1_0332	38	56	29	41	14
rpoE_2	47	31	45	41	9
glgA_2	33	40	50	41	8
queH	44	35	43	41	5
MTHMO_v1_0502	33	26	63	41	20
nifV	38	56	28	41	14
MTHMO_v1_0323	33	89	0	41	45
uvrA_2	21	54	47	41	17
MTHMO_v1_1664	0	26	95	41	49
cdaA	36	33	52	40	10
MTHMO_v1_1644	0	58	63	40	35
mutS	44	29	48	40	10
MTHMO_v1_1993	35	44	42	40	5
MTHMO_v1_2123	19	46	55	40	19
MTHMO_v1_1956	43	60	17	40	22
MTHMO_v1_1533	49	71	0	40	36
MTHMO_v1_1394	0	120	0	40	69
priA	49	34	36	40	8
MTHMO_v1_1324	42	62	16	40	23
MTHMO_v1_0479	35	28	56	40	15
pdxA	60	52	7	40	28
MTHMO_v1_2181	0	32	87	40	44
MTHMO_v1_0080	56	39	24	40	16
MTHMO_v1_1649	37	43	38	40	3
MTHMO_v1_1955	57	61	0	39	34
coaX	48	38	31	39	9
MTHMO_v1_1410	41	19	57	39	19
MTHMO_v1_0743	45	72	0	39	36
MTHMO_v1_1449	8	39	70	39	31
fixC	36	36	45	39	5
MTHMO_v1_2083	40	42	34	39	4
MTHMO_v1_0742	54	20	41	38	17
MTHMO_v1_0166	38	40	36	38	2
MTHMO_v1_0538	45	33	36	38	7
MTHMO_v1_1005	51	14	49	38	21
MTHMO_v1_0659	40	49	25	38	12
MTHMO_v1_2254	31	44	40	38	7
MTHMO_v1_1576	76	13	24	38	33
MTHMO_v1_0726	54	32	29	38	14

MTHMO_v1_0002	0	52	62	38	33
MTHMO_v1_1467	36	52	26	38	13
MTHMO_v1_0240	46	0	67	38	34
nifH	60	24	29	38	20
MTHMO_v1_0506	57	10	46	38	25
MTHMO_v1_1552	34	44	35	38	5
MTHMO_v1_1377	37	40	36	37	2
MTHMO_v1_0965	33	55	24	37	16
MTHMO_v1_0433	0	47	64	37	33
MTHMO_v1_0012	39	36	37	37	1
MTHMO_v1_2168	56	22	34	37	17
MTHMO_v1_0100	35	37	40	37	3
MTHMO_v1_0640	77	23	11	37	35
MTHMO_v1_2084	29	27	56	37	16
MTHMO_v1_0158	37	39	35	37	2
tmk	51	49	10	37	23
MTHMO_v1_0614	39	43	28	37	8
MTHMO_v1_0072	7	78	26	37	37
MTHMO_v1_0749	52	41	17	36	18
lysR_2	37	30	42	36	6
MTHMO_v1_1361	16	47	46	36	18
MTHMO_v1_1547	46	36	28	36	9
MTHMO_v1_0925	0	17	92	36	49
MTHMO_v1_0551	65	23	21	36	25
MTHMO_v1_1193	57	31	21	36	19
MTHMO_v1_1346	34	30	44	36	7
MTHMO_v1_1689	14	38	55	36	21
MTHMO_v1_1916	59	10	38	36	24
MTHMO_v1_1968	30	40	38	36	5
MTHMO_v1_0230	36	32	40	36	4
pyrE	37	59	12	36	23
MTHMO_v1_2155	42	59	7	36	27
MTHMO_v1_1363	37	16	53	36	19
MTHMO_v1_0030	29	44	34	36	8
MTHMO_v1_1909	13	42	51	35	20
ybhF	54	31	22	35	16
MTHMO_v1_0574	0	73	33	35	37
MTHMO_v1_1033	12	52	41	35	21
MTHMO_v1_1840	76	30	0	35	38
MTHMO_v1_0579	45	33	27	35	9
moaE	63	42	0	35	32
znuB	46	29	30	35	10

MTHMO_v1_0093	25	37	43	35	9
MTHMO_v1_1414	27	40	38	35	7
MTHMO_v1_0091	35	30	40	35	5
MTHMO_v1_0026	44	23	37	35	10
recO	0	45	58	34	31
deoC	29	46	28	34	10
queA	45	34	25	34	10
MTHMO_v1_2018	20	21	62	34	24
MTHMO_v1_0484	46	16	40	34	16
MTHMO_v1_1307	46	38	18	34	14
cas2	68	12	22	34	30
modA	34	36	32	34	2
spr	26	42	34	34	8
MTHMO_v1_0686	49	16	38	34	17
uvrA_1	14	48	40	34	18
amtB_1	16	50	35	34	17
MTHMO_v1_1244	34	34	33	34	1
MTHMO_v1_1672	33	38	31	34	3
MTHMO_v1_1796	44	35	21	34	12
MTHMO_v1_1730	37	43	21	34	11
gmhA	32	28	41	33	6
MTHMO_v1_1579	55	18	27	33	19
MTHMO_v1_0418	40	27	32	33	7
MTHMO_v1_2248	44	23	32	33	10
MTHMO_v1_0884	22	35	42	33	10
glcF	30	29	39	33	5
MTHMO_v1_0218	36	32	31	33	3
MTHMO_v1_1494	24	26	47	33	13
MTHMO_v1_0449	37	26	35	33	6
MTHMO_v1_1842	19	54	24	32	19
MTHMO_v1_1538	19	72	6	32	35
MTHMO_v1_1558	53	22	23	32	18
MTHMO_v1_1640	45	34	18	32	14
MTHMO_v1_1930	31	21	45	32	12
ispE	38	36	22	32	9
MTHMO_v1_1444	40	40	16	32	13
MTHMO_v1_1425	37	15	44	32	15
kdpC	22	30	43	32	10
MTHMO_v1_0630	30	34	32	32	2
MTHMO_v1_0961	39	47	9	32	20
MTHMO_v1_0465	44	20	30	31	12
MTHMO_v1_1694	26	48	21	31	15
MTHMO_v1_0220	41	33	20	31	11

MTHMO_v1_0776	54	5	35	31	25
MTHMO_v1_0515	59	27	8	31	26
nifE	29	46	19	31	14
frxA	61	33	0	31	31
bioD	49	16	28	31	17
MTHMO_v1_1312	39	31	23	31	8
MTHMO_v1_1323	21	51	21	31	18
MTHMO_v1_1729	26	31	35	31	4
MTHMO_v1_0451	26	26	40	31	8
MTHMO_v1_1724	0	33	59	31	30
MTHMO_v1_2358	36	41	15	31	14
MTHMO_v1_0818	36	29	26	31	5
MTHMO_v1_1127	25	24	43	31	11
MTHMO_v1_2149	46	0	45	30	26
MTHMO_v1_0429	59	31	0	30	30
MTHMO_v1_0066	35	56	0	30	28
cas4-cas	27	37	26	30	6
rsmA	52	14	25	30	19
MTHMO_v1_0503	33	30	27	30	3
MTHMO_v1_1214	33	26	31	30	3
MTHMO_v1_1731	66	23	0	30	34
MTHMO_v1_1850	31	28	30	30	2
MTHMO_v1_1310	42	35	12	30	16
MTHMO_v1_1978	34	14	41	30	14
MTHMO_v1_0633	25	40	24	30	9
bioF	41	25	23	30	10
kdpE	37	25	27	30	7
tolC_3	23	27	39	30	9
MTHMO_v1_0483	8	31	49	30	20
MTHMO_v1_0424	35	19	34	29	9
plsC	44	12	32	29	16
MTHMO_v1_1907	40	48	0	29	26
MTHMO_v1_0164	29	31	28	29	1
MTHMO_v1_0536	24	27	36	29	6
kup	25	35	27	29	6
MTHMO_v1_2161	49	26	12	29	19
MTHMO_v1_1859	30	28	29	29	1
MTHMO_v1_0915	29	30	27	29	1
iorA	43	30	14	29	15
MTHMO_v1_0364	12	45	29	29	17
MTHMO_v1_1740	39	10	37	29	16
MTHMO_v1_1915	28	30	27	29	1

MTHMO_v1_0134	17	36	32	28	10
rluA	9	42	34	28	17
MTHMO_v1_0808	34	18	33	28	9
MTHMO_v1_0817	29	27	28	28	1
MTHMO_v1_1597	24	38	23	28	8
MTHMO_v1_1636	24	13	47	28	17
MTHMO_v1_0409	49	35	0	28	25
MTHMO_v1_2295	23	33	27	28	5
MTHMO_v1_0809	21	33	30	28	6
MTHMO_v1_1476	17	12	54	28	23
MTHMO_v1_1667	16	35	32	28	10
czcA_2	29	32	22	28	5
MTHMO_v1_1798	33	23	26	28	5
MTHMO_v1_1211	70	12	0	27	37
MTHMO_v1_1623	0	40	42	27	24
thiD	33	17	32	27	9
pyrD	6	35	40	27	19
tsaE	16	50	15	27	20
MTHMO_v1_1769	53	28	0	27	27
MTHMO_v1_0798	40	21	19	27	11
MTHMO_v1_0257	36	19	26	27	8
davD	14	35	31	27	11
MTHMO_v1_1610	22	15	43	27	14
MTHMO_v1_1390	24	32	23	27	5
MTHMO_v1_1450	20	21	38	26	10
MTHMO_v1_1271	22	35	21	26	8
MTHMO_v1_0097	24	32	23	26	5
rsfS	0	60	18	26	31
MTHMO_v1_0137	0	49	29	26	25
MTHMO_v1_0269	41	27	10	26	16
MTHMO_v1_1411	51	27	0	26	25
MTHMO_v1_2359	31	23	24	26	4
MTHMO_v1_0883	35	22	20	26	8
MTHMO_v1_1569	33	13	32	26	11
MTHMO_v1_0796	33	27	16	25	9
bioA	44	13	19	25	16
fixU	30	16	29	25	8
MTHMO_v1_0497	9	30	36	25	14
MTHMO_v1_1270	25	26	24	25	1
MTHMO_v1_2179	27	32	16	25	8
MTHMO_v1_1613	18	39	18	25	12
MTHMO_v1_1944	23	29	22	25	4
MTHMO_v1_0377	45	18	11	25	18

MTHMO_v1_1227	25	25	24	25	0
MTHMO_v1_1207	12	26	36	25	12
salY	21	25	28	25	3
MTHMO_v1_0922	36	29	9	25	14
MTHMO_v1_0231	10	33	30	25	12
yqxC	48	17	8	24	21
MTHMO_v1_0747	0	16	57	24	30
MTHMO_v1_0532	0	26	47	24	23
MTHMO_v1_0413	0	45	27	24	23
MTHMO_v1_1498	24	31	17	24	7
MTHMO_v1_1979	6	33	33	24	16
MTHMO_v1_0500	20	32	19	24	7
MTHMO_v1_1685	51	11	10	24	24
MTHMO_v1_1989	43	29	0	24	22
mraW	42	23	7	24	18
dfp	10	32	29	24	12
dauA	12	31	28	24	10
MTHMO_v1_0095	21	39	10	23	15
MTHMO_v1_0955	0	21	49	23	24
MTHMO_v1_0466	13	33	24	23	10
urh	12	35	23	23	11
yfnA	18	29	22	23	6
MTHMO_v1_1924	54	14	0	23	28
MTHMO_v1_1228	38	8	22	23	15
MTHMO_v1_0103	24	25	20	23	3
MTHMO_v1_0507	0	69	0	23	40
MTHMO_v1_1499	25	31	12	23	10
MTHMO_v1_1158	35	0	34	23	20
MTHMO_v1_1036	41	16	11	23	16
pqqD	23	24	22	23	1
MTHMO_v1_1784	11	31	26	23	11
MTHMO_v1_1876	58	10	0	23	31
MTHMO_v1_1654	0	68	0	23	39
nasA	28	29	11	23	10
MTHMO_v1_0437	0	17	51	22	26
MTHMO_v1_1186	17	27	24	22	5
MTHMO_v1_0851	9	50	8	22	24
MTHMO_v1_0995	44	23	0	22	22
MTHMO_v1_0882	57	10	0	22	30
MTHMO_v1_0417	16	23	26	22	5
wrbA	11	35	21	22	12
MTHMO_v1_0386	24	19	23	22	3

znuC	26	23	17	22	5
MTHMO_v1_1129	52	14	0	22	27
MTHMO_v1_0745	36	23	7	22	15
MTHMO_v1_0247	19	23	23	22	2
MTHMO_v1_1483	0	34	31	22	19
MTHMO_v1_1965	26	32	8	22	12
plsY	10	36	19	22	13
MTHMO_v1_1580	36	15	14	22	12
rbbA	25	31	8	22	12
MTHMO_v1_0079	30	22	12	21	9
nifW	33	0	31	21	18
MTHMO_v1_0613	22	21	21	21	0
MTHMO_v1_2263	23	30	11	21	10
MTHMO_v1_1278	0	63	0	21	36
MTHMO_v1_1194	14	14	35	21	12
cirA_2	23	15	25	21	5
nsrR	46	16	0	21	24
tolC_4	18	28	17	21	6
MTHMO_v1_1549	23	17	22	21	4
MTHMO_v1_0491	28	18	16	21	6
MTHMO_v1_1675	21	41	0	21	21
MTHMO_v1_0816	36	19	7	21	14
cas_1	0	28	33	20	18
birA	27	14	19	20	6
cirA_4	16	22	22	20	3
MTHMO_v1_1262	0	0	60	20	35
MTHMO_v1_1368	20	21	19	20	1
paeR7IR	27	24	9	20	10
MTHMO_v1_2131	17	9	33	20	12
MTHMO_v1_1958	26	21	13	20	7
nifN	19	31	9	20	11
MTHMO_v1_1496	20	25	14	20	5
MTHMO_v1_1481	29	30	0	20	17
cirA_7	26	10	23	20	9
atpB_1	10	10	38	19	16
MTHMO_v1_1260	20	25	13	19	6
murB_2	6	27	24	19	11
MTHMO_v1_1896	13	38	6	19	17
MTHMO_v1_0460	16	26	16	19	6
MTHMO_v1_2035	22	8	28	19	10
MTHMO_v1_1204	19	31	6	19	12
MTHMO_v1_0917	16	28	12	19	9
MTHMO_v1_1247	18	16	23	19	4

MTHMO_v1_1714	30	16	10	19	10
MTHMO_v1_1622	42	14	0	19	21
MTHMO_v1_0373	36	19	0	19	18
MTHMO_v1_1852	0	0	56	19	32
gpsA	15	12	29	19	9
MTHMO_v1_1009	27	29	0	18	16
MTHMO_v1_0914	0	55	0	18	32
MTHMO_v1_1387	10	26	19	18	8
MTHMO_v1_1726	11	23	21	18	7
MTHMO_v1_0994	23	16	15	18	4
MTHMO_v1_1461	15	39	0	18	20
MTHMO_v1_1935	16	22	16	18	3
glcE	19	17	18	18	1
MTHMO_v1_0073	5	30	18	18	13
MTHMO_v1_1670	32	11	10	18	12
nifA	21	13	20	18	4
MTHMO_v1_1495	34	18	0	18	17
MTHMO_v1_2238	23	6	22	17	10
MTHMO_v1_0794	20	0	32	17	16
MTHMO_v1_0773	23	6	22	17	10
MTHMO_v1_1614	36	16	0	17	18
MTHMO_v1_0872	0	52	0	17	30
MTHMO_v1_1996	0	52	0	17	30
MTHMO_v1_1376	0	23	28	17	15
ribD	19	20	12	17	4
MTHMO_v1_1027	14	23	14	17	5
smtA_2	6	14	31	17	13
MTHMO_v1_0792	51	0	0	17	29
MTHMO_v1_1457	24	26	0	17	15
MTHMO_v1_1236	20	11	19	17	5
MTHMO_v1_1203	50	0	0	17	29
tonB_2	18	14	17	17	2
MTHMO_v1_0308	30	11	10	17	11
MTHMO_v1_1222	0	31	19	17	16
MTHMO_v1_1980	10	19	20	17	5
MTHMO_v1_1592	32	17	0	17	16
MTHMO_v1_1226	0	35	14	16	18
MTHMO_v1_2079	0	33	15	16	17
MTHMO_v1_2034	0	35	13	16	18
MTHMO_v1_2150	31	16	0	16	15
MTHMO_v1_0157	0	47	0	16	27
MTHMO_v1_1682	0	17	30	16	15

MTHMO_v1_1627	6	23	18	16	9
MTHMO_v1_2082	31	16	0	16	15
arsC	0	21	26	16	14
MTHMO_v1_0841	0	32	14	16	16
MTHMO_v1_2180	13	14	19	15	3
MTHMO_v1_1459	19	18	9	15	5
MTHMO_v1_2146	11	25	10	15	9
MTHMO_v1_1681	4	24	17	15	10
MTHMO_v1_1427	0	37	8	15	20
MTHMO_v1_0845	15	16	14	15	1
MTHMO_v1_0819	15	0	30	15	15
merT	0	28	17	15	14
MTHMO_v1_1543	0	45	0	15	26
MTHMO_v1_0440	18	13	14	15	2
MTHMO_v1_1451	5	19	20	15	8
MTHMO_v1_0581	0	16	28	15	14
MTHMO_v1_1905	23	14	6	15	8
prmC	16	4	23	15	10
MTHMO_v1_0585	24	19	0	15	13
MTHMO_v1_0939	18	13	12	14	4
yadR	17	27	0	14	13
MTHMO_v1_1502	8	23	12	14	8
MTHMO_v1_1474	16	22	5	14	9
ddpA	15	24	4	14	10
nfo	13	17	12	14	3
ybhS	6	19	17	14	7
arsM	0	33	9	14	17
MTHMO_v1_2036	11	3	27	14	12
smtA_3	9	15	18	14	4
MTHMO_v1_1261	0	27	14	14	14
MTHMO_v1_1170	0	0	41	14	24
MTHMO_v1_0430	6	17	18	14	6
MTHMO_v1_0017	0	19	22	14	12
MTHMO_v1_0584	0	41	0	14	24
MTHMO_v1_1643	0	0	41	14	23
iscA	21	0	20	13	12
MTHMO_v1_1165	22	18	0	13	12
MTHMO_v1_0578	0	12	28	13	14
MTHMO_v1_0814	19	20	0	13	11
MTHMO_v1_0576	0	14	26	13	13
MTHMO_v1_0235	26	0	13	13	13
MTHMO_v1_0064	0	20	18	13	11
MTHMO_v1_2037	0	28	10	13	14

MTHMO_v1_0625	15	16	7	13	5
ubiE_2	8	22	8	13	8
MTHMO_v1_1899	25	4	8	13	11
MTHMO_v1_1311	37	0	0	12	22
MTHMO_v1_0428	11	21	5	12	8
nifB	16	17	4	12	7
MTHMO_v1_0401	0	0	37	12	21
thiL	14	15	7	12	5
MTHMO_v1_1743	15	8	14	12	4
xdhA	19	8	9	12	6
MTHMO_v1_1791	13	5	17	12	6
MTHMO_v1_2017	0	0	35	12	20
MTHMO_v1_2183	16	4	15	12	7
bglX	3	17	14	11	8
MTHMO_v1_0029	11	23	0	11	12
MTHMO_v1_1596	11	3	21	11	9
MTHMO_v1_1243	12	11	11	11	1
MTHMO_v1_0903	0	34	0	11	20
murJ	4	15	15	11	6
MTHMO_v1_1469	0	0	34	11	19
hisC_1	6	6	22	11	9
MTHMO_v1_1679	8	17	8	11	5
MTHMO_v1_0802	33	0	0	11	19
MTHMO_v1_0797	33	0	0	11	19
MTHMO_v1_0068	0	33	0	11	19
MTHMO_v1_2353	0	33	0	11	19
MTHMO_v1_1263	22	4	7	11	10
MTHMO_v1_1739	0	12	21	11	11
MTHMO_v1_1609	0	0	32	11	19
MTHMO_v1_1757	11	6	16	11	5
MTHMO_v1_0750	12	20	0	11	10
MTHMO_v1_0647	32	0	0	11	18
MTHMO_v1_0509	0	11	20	11	10
MTHMO_v1_1252	0	0	32	11	18
MTHMO_v1_1048	0	7	25	10	13
MTHMO_v1_1626	16	0	15	10	9
MTHMO_v1_1783	0	11	20	10	10
rfaG_2	11	9	11	10	1
MTHMO_v1_1473	10	11	10	10	1
MTHMO_v1_1629	11	14	5	10	5
MTHMO_v1_0421	0	0	30	10	17
MTHMO_v1_1041	8	14	8	10	3

MTHMO_v1_0795	0	14	16	10	9
xdhB	8	13	8	10	3
MTHMO_v1_0161	0	30	0	10	17
MTHMO_v1_1683	9	12	9	10	2
MTHMO_v1_1026	15	0	14	10	8
MTHMO_v1_0779	11	18	0	10	9
miaA	0	15	14	10	8
dbh	19	10	0	10	9
MTHMO_v1_1246	6	12	11	9	3
MTHMO_v1_1484	0	28	0	9	16
dtd	0	0	28	9	16
virD	12	10	6	9	3
MTHMO_v1_2160	8	8	11	9	2
MTHMO_v1_2038	27	0	0	9	16
MTHMO_v1_1241	0	27	0	9	16
MTHMO_v1_0160	0	0	27	9	16
fixB	6	10	11	9	3
MTHMO_v1_0436	0	12	15	9	8
MTHMO_v1_0918	27	0	0	9	15
MTHMO_v1_1213	27	0	0	9	15
MTHMO_v1_0258	9	9	8	9	0
fixA	8	4	15	9	5
MTHMO_v1_0778	10	6	10	9	3
MTHMO_v1_0241	0	26	0	9	15
MTHMO_v1_0916	0	26	0	9	15
MTHMO_v1_0887	0	25	0	8	15
MTHMO_v1_0989	0	25	0	8	15
MTHMO_v1_0394	0	0	25	8	15
merA	8	9	8	8	0
MTHMO_v1_1628	0	14	10	8	7
MTHMO_v1_1668	6	7	12	8	3
MTHMO_v1_0387	10	5	10	8	3
MTHMO_v1_1696	12	13	0	8	7
repA	0	24	0	8	14
MTHMO_v1_1603	0	9	16	8	8
virB	5	9	10	8	3
mxaR	7	11	6	8	2
MTHMO_v1_2244	24	0	0	8	14
MTHMO_v1_0326	0	24	0	8	14
MTHMO_v1_0067	7	11	6	8	2
fixX	23	0	0	8	13
MTHMO_v1_1917	15	8	0	8	7
capI	13	3	6	8	5

MTHMO_v1_1391	0	23	0	8	13
MTHMO_v1_1217	0	0	23	8	13
MTHMO_v1_0582	23	0	0	8	13
ade	7	2	14	8	6
MTHMO_v1_0392	10	8	5	8	3
MTHMO_v1_2156	0	0	21	7	12
MTHMO_v1_1742	0	20	0	7	12
MTHMO_v1_1256	10	0	10	7	6
MTHMO_v1_1637	0	20	0	7	12
nifS_2	11	3	5	7	4
MTHMO_v1_1470	8	4	8	7	2
MTHMO_v1_1040	0	20	0	7	11
MTHMO_v1_1428	0	20	0	7	11
MTHMO_v1_0577	13	7	0	7	6
MTHMO_v1_0844	0	0	20	7	11
MTHMO_v1_2080	10	0	10	6	6
MTHMO_v1_0820	7	12	0	6	6
MTHMO_v1_1218	5	9	5	6	2
MTHMO_v1_1192	0	10	9	6	6
nirB	5	6	8	6	1
MTHMO_v1_1460	0	4	15	6	8
MTHMO_v1_0508	0	19	0	6	11
MTHMO_v1_2220	0	19	0	6	11
MTHMO_v1_1200	19	0	0	6	11
MTHMO_v1_1240	0	18	0	6	10
MTHMO_v1_0964	0	18	0	6	10
MTHMO_v1_0793	0	0	18	6	10
MTHMO_v1_0580	0	18	0	6	10
MTHMO_v1_2187	0	17	0	6	10
MTHMO_v1_0854	0	17	0	6	10
MTHMO_v1_0859	0	17	0	6	10
MTHMO_v1_1231	9	0	8	6	5
MTHMO_v1_1553	0	17	0	6	10
MTHMO_v1_0774	0	4	13	6	7
cas_2	0	17	0	6	10
MTHMO_v1_0853	0	6	10	5	5
MTHMO_v1_0780	0	16	0	5	9
MTHMO_v1_1463	0	6	10	5	5
MTHMO_v1_0520	16	0	0	5	9
MTHMO_v1_1480	0	15	0	5	9
MTHMO_v1_0902	0	15	0	5	9
MTHMO_v1_1581	0	8	7	5	4

MTHMO_v1_2186	0	15	0	5	9
MTHMO_v1_0400	0	10	5	5	5
MTHMO_v1_1906	10	5	0	5	5
MTHMO_v1_1471	0	14	0	5	8
MTHMO_v1_0499	4	2	8	5	3
MTHMO_v1_1666	0	14	0	5	8
MTHMO_v1_2178	0	13	0	4	8
MTHMO_v1_1934	0	13	0	4	8
menG	0	5	8	4	4
guaD	13	0	0	4	7
pulE	0	3	9	4	5
MTHMO_v1_1029	3	6	3	4	2
zraR_1	0	3	10	4	5
MTHMO_v1_1242	0	12	0	4	7
proP_1	5	3	5	4	1
MTHMO_v1_0156	0	12	0	4	7
MTHMO_v1_1196	0	2	9	4	5
MTHMO_v1_0505	0	11	0	4	7
MTHMO_v1_1671	0	4	7	4	4
MTHMO_v1_1038	0	4	7	4	4
MTHMO_v1_1257	7	4	0	4	4
MTHMO_v1_1249	0	4	7	4	4
dedA	0	11	0	4	6
MTHMO_v1_1237	11	0	0	4	6
allB	5	5	0	3	3
MTHMO_v1_1588	0	10	0	3	6
MTHMO_v1_0837	0	2	7	3	4
MTHMO_v1_0519	10	0	0	3	6
sufA	0	10	0	3	6
tonB_3	0	0	10	3	6
MTHMO_v1_1031	0	3	6	3	3
MTHMO_v1_0963	0	9	0	3	5
MTHMO_v1_1224	0	0	9	3	5
MTHMO_v1_1223	9	0	0	3	5
MTHMO_v1_1275	6	3	0	3	3
MTHMO_v1_1052	0	3	5	3	3
argE_2	5	3	0	3	3
dnaE_2	0	8	0	3	5
MTHMO_v1_1789	0	8	0	3	5
MTHMO_v1_1788	7	0	0	2	4
glxB	7	0	0	2	4
MTHMO_v1_1932	0	0	7	2	4
MTHMO_v1_1651	0	6	0	2	4

MTHMO_v1_1235	0	6	0	2	4
MTHMO_v1_1254	0	5	0	2	3
MTHMO_v1_1690	0	5	0	2	3
MTHMO_v1_0803	0	4	0	1	2
MTHMO_v1_1652	0	4	0	1	2
hcaE	0	4	0	1	2
MTHMO_v1_1465	0	3	0	1	2
MTHMO_v1_1229	0	3	0	1	2
MTHMO_v1_0003	0	0	0	0	0
MTHMO_v1_0011	0	0	0	0	0
MTHMO_v1_0014	0	0	0	0	0
MTHMO_v1_0062	0	0	0	0	0
MTHMO_v1_0065	0	0	0	0	0
MTHMO_v1_0074	0	0	0	0	0
MTHMO_v1_0075	0	0	0	0	0
MTHMO_v1_0104	0	0	0	0	0
MTHMO_v1_0105	0	0	0	0	0
MTHMO_v1_0125	0	0	0	0	0
MTHMO_v1_0152	0	0	0	0	0
MTHMO_v1_0153	0	0	0	0	0
MTHMO_v1_0154	0	0	0	0	0
MTHMO_v1_0155	0	0	0	0	0
MTHMO_v1_0159	0	0	0	0	0
MTHMO_v1_0163	0	0	0	0	0
MTHMO_v1_0165	0	0	0	0	0
MTHMO_v1_0221	0	0	0	0	0
MTHMO_v1_0222	0	0	0	0	0
MTHMO_v1_0223	0	0	0	0	0
MTHMO_v1_0226	0	0	0	0	0
MTHMO_v1_0233	0	0	0	0	0
MTHMO_v1_0236	0	0	0	0	0
MTHMO_v1_0242	0	0	0	0	0
MTHMO_v1_0276	0	0	0	0	0
MTHMO_v1_0297	0	0	0	0	0
MTHMO_v1_0304	0	0	0	0	0
MTHMO_v1_0324	0	0	0	0	0
MTHMO_v1_0325	0	0	0	0	0
MTHMO_v1_0341	0	0	0	0	0
MTHMO_v1_0389	0	0	0	0	0
MTHMO_v1_0395	0	0	0	0	0
argE_3	0	0	0	0	0
MTHMO_v1_0403	0	0	0	0	0

MTHMO_v1_0411	0	0	0	0	0
MTHMO_v1_0419	0	0	0	0	0
MTHMO_v1_0420	0	0	0	0	0
MTHMO_v1_0435	0	0	0	0	0
MTHMO_v1_0450	0	0	0	0	0
MTHMO_v1_0452	0	0	0	0	0
MTHMO_v1_0454	0	0	0	0	0
MTHMO_v1_0457	0	0	0	0	0
MTHMO_v1_0487	0	0	0	0	0
MTHMO_v1_0489	0	0	0	0	0
MTHMO_v1_0501	0	0	0	0	0
MTHMO_v1_0504	0	0	0	0	0
MTHMO_v1_0573	0	0	0	0	0
MTHMO_v1_0583	0	0	0	0	0
MTHMO_v1_0622	0	0	0	0	0
MTHMO_v1_0657	0	0	0	0	0
amtB_2	0	0	0	0	0
MTHMO_v1_0758	0	0	0	0	0
MTHMO_v1_0760	0	0	0	0	0
MTHMO_v1_0807	0	0	0	0	0
MTHMO_v1_0811	0	0	0	0	0
MTHMO_v1_0838	0	0	0	0	0
MTHMO_v1_0840	0	0	0	0	0
MTHMO_v1_0855	0	0	0	0	0
MTHMO_v1_0892	0	0	0	0	0
MTHMO_v1_0936	0	0	0	0	0
MTHMO_v1_0962	0	0	0	0	0
MTHMO_v1_0968	0	0	0	0	0
MTHMO_v1_0969	0	0	0	0	0
MTHMO_v1_0993	0	0	0	0	0
MTHMO_v1_1030	0	0	0	0	0
MTHMO_v1_1037	0	0	0	0	0
nifZ	0	0	0	0	0
MTHMO_v1_1053	0	0	0	0	0
MTHMO_v1_1182	0	0	0	0	0
MTHMO_v1_1197	0	0	0	0	0
MTHMO_v1_1198	0	0	0	0	0
MTHMO_v1_1201	0	0	0	0	0
MTHMO_v1_1202	0	0	0	0	0
MTHMO_v1_1205	0	0	0	0	0
MTHMO_v1_1216	0	0	0	0	0
MTHMO_v1_1225	0	0	0	0	0
MTHMO_v1_1233	0	0	0	0	0

MTHMO_v1_1234	0	0	0	0	0
MTHMO_v1_1238	0	0	0	0	0
MTHMO_v1_1250	0	0	0	0	0
MTHMO_v1_1251	0	0	0	0	0
MTHMO_v1_1253	0	0	0	0	0
MTHMO_v1_1255	0	0	0	0	0
MTHMO_v1_1258	0	0	0	0	0
MTHMO_v1_1259	0	0	0	0	0
MTHMO_v1_1267	0	0	0	0	0
MTHMO_v1_1272	0	0	0	0	0
MTHMO_v1_1273	0	0	0	0	0
MTHMO_v1_1276	0	0	0	0	0
MTHMO_v1_1279	0	0	0	0	0
MTHMO_v1_1281	0	0	0	0	0
MTHMO_v1_1305	0	0	0	0	0
MTHMO_v1_1314	0	0	0	0	0
MTHMO_v1_1318	0	0	0	0	0
MTHMO_v1_1329	0	0	0	0	0
MTHMO_v1_1333	0	0	0	0	0
MTHMO_v1_1339	0	0	0	0	0
MTHMO_v1_1350	0	0	0	0	0
MTHMO_v1_1374	0	0	0	0	0
MTHMO_v1_1384	0	0	0	0	0
MTHMO_v1_1399	0	0	0	0	0
MTHMO_v1_1426	0	0	0	0	0
MTHMO_v1_1437	0	0	0	0	0
MTHMO_v1_1447	0	0	0	0	0
MTHMO_v1_1458	0	0	0	0	0
cydA	0	0	0	0	0
MTHMO_v1_1482	0	0	0	0	0
rplV	0	0	0	0	0
MTHMO_v1_1554	0	0	0	0	0
MTHMO_v1_1584	0	0	0	0	0
MTHMO_v1_1590	0	0	0	0	0
MTHMO_v1_1599	0	0	0	0	0
MTHMO_v1_1615	0	0	0	0	0
MTHMO_v1_1624	0	0	0	0	0
MTHMO_v1_1650	0	0	0	0	0
MTHMO_v1_1653	0	0	0	0	0
MTHMO_v1_1661	0	0	0	0	0
MTHMO_v1_1669	0	0	0	0	0
MTHMO_v1_1678	0	0	0	0	0

MTHMO_v1_1684	0	0	0	0	0
MTHMO_v1_1759	0	0	0	0	0
MTHMO_v1_1782	0	0	0	0	0
MTHMO_v1_1786	0	0	0	0	0
MTHMO_v1_1790	0	0	0	0	0
MTHMO_v1_1797	0	0	0	0	0
rplU	0	0	0	0	0
MTHMO_v1_1856	0	0	0	0	0
MTHMO_v1_1877	0	0	0	0	0
MTHMO_v1_1969	0	0	0	0	0
MTHMO_v1_1971	0	0	0	0	0
MTHMO_v1_1975	0	0	0	0	0
glpF	0	0	0	0	0
MTHMO_v1_2011	0	0	0	0	0
MTHMO_v1_2078	0	0	0	0	0
merE	0	0	0	0	0
MTHMO_v1_2154	0	0	0	0	0
MTHMO_v1_2157	0	0	0	0	0
MTHMO_v1_2166	0	0	0	0	0
MTHMO_v1_2167	0	0	0	0	0
MTHMO_v1_2169	0	0	0	0	0
MTHMO_v1_2184	0	0	0	0	0
MTHMO_v1_2185	0	0	0	0	0
MTHMO_v1_2261	0	0	0	0	0
MTHMO_v1_2274	0	0	0	0	0
MTHMO_v1_2289	0	0	0	0	0
MTHMO_v1_2309	0	0	0	0	0
MTHMO_v1_2315	0	0	0	0	0
MTHMO_v1_2354	0	0	0	0	0

Table S5. Genes involved in H_2 metabolism in strain AP8 and their transcriptome profile. The annotation was done on MicrosCope platform using protein-BLAST and HydDB web tool for hydrogenase classification (http://services.birc.au.dk/hyddb/). The mRNA expression level is shown in RPKM.

**Gene ID**	**Gene name**	**Protein annotation**	**RPKM**
MTHMO_v1_1378	*hynA*	Group 1b [NiFe] hydrogenase small subunit	802
MTHMO_v1_1379	*hynB*	Group 1b [NiFe] hydrogenase large subunit	743
MTHMO_v1_1380	*hynC*	[NiFe] hydrogenase cytochrome b subunit	336
MTHMO_v1_1366	*hypC*	Hydrogenase assembly chaperone	286
MTHMO_v1_1369	*hypB*	Hydrogenase maturation factor	178
MTHMO_v1_1367	*hypF*	Hydrogenase expression/formation protein	144
MTHMO_v1_1365	*hypD*	Assembly hydrogenase maturation factor	128
MTHMO_v1_1381	*hupD*	Putative hydrogenase maturation protease	118
MTHMO_v1_1370	*hypA*	Hydrogenase maturation factor	104
MTHMO_v1_1364	*hypE*	Hydrogenase expression/formation protein	88
MTHMO_v1_1497	*hupQ*	Hydrogenase expression/formation protein HupQ	68
